# Effects of hypoxic training interventions on cardiometabolic health of adults with overweight and obesity: A systematic review and meta‐analysis

**DOI:** 10.1111/dom.70303

**Published:** 2025-11-18

**Authors:** Alessandro Gatti, Caterina Cavallo, Matteo Giuriato, Agnese Pirazzi, Vittoria Carnevale Pellino, Nicola Lovecchio, Stefano Lazzer, Virginia Rossi, Valeria Calcaterra, Gianvincenzo Zuccotti, Anna Odone, Alba Camacho‐Cardenosa, Matteo Vandoni

**Affiliations:** ^1^ Laboratory of Adapted Motor Activity (LAMA), Department of Public Health, Experimental Medicine and Forensic Science University of Pavia Pavia Italy; ^2^ National PhD Programme in One Health Approaches to Infectious Diseases and Life Science Research, Department of Public Health, Experimental and Forensic Medicine University of Pavia Pavia Italy; ^3^ Department of Sport LUNEX University of Applied Sciences Differdange Luxembourg; ^4^ Department of Human and Social Science University of Bergamo Bergamo Italy; ^5^ School of Sport Sciences University of Udine Udine Italy; ^6^ Department of Medicine University of Udine Udine Italy; ^7^ Pediatric Department “Vittore Buzzi” Children's Hospital Milan Italy; ^8^ Pediatric and Adolescent Unit, Department of Internal Medicine University of Pavia Pavia Italy; ^9^ Department of Biomedical and Clinical Science University of Milano Milan Italy; ^10^ Department of Public Health, Experimental and Forensic Medicine University of Pavia Pavia Italy; ^11^ Department of Physical Education and Sports Faculty of Sport Sciences, Sport and Health University Research Institute (iMUDS), University of Granada Granada Spain; ^12^ Instituto de Investigación Biosanitaria ibs GRANADA Granada Spain

**Keywords:** cardiovascular disease, meta‐analysis, systematic review, weight management

## Abstract

Obesity rates have surpassed underweight globally, increasing the burden of cardiometabolic complications on healthcare systems. Hypoxic training has emerged as a potential intervention to improve cardiometabolic health in adults with obesity, but evidence remains inconclusive. This systematic review and meta‐analysis evaluated whether hypoxic training is more effective than normoxic training in this context. A systematic search of PubMed, Web of Science, and Cochrane Library (up to June 2025) identified randomised controlled trials comparing hypoxic and normoxic training in adults with overweight or obesity. Outcomes included glucose homeostasis, lipid profile, and blood pressure. Subgroup, moderation, and sensitivity analyses were also conducted to explore sources of heterogeneity and assess the robustness of findings. Of 1815 studies screened, 9 (278 participants) met the criteria. Meta‐analysis results demonstrated no significant differences between hypoxic and normoxic training for fasting glucose (*p* = 0.118) or fasting insulin (*p* = 0.415), with substantial heterogeneity observed across studies (*I* ^2^ = 60%–77%). Similarly, lipid profile markers and blood pressure showed no significant between‐group differences (all *p* > 0.05), also with moderate to high heterogeneity. Subgroup and moderation analyses partially explained this variability, suggesting greater fasting glucose reductions with shorter and lower‐intensity hypoxic interventions. Hypoxic training did not outperform normoxic training in improving cardiometabolic outcomes. However, the considerable variability in intervention duration, hypoxic dose, and exercise intensity across studies limits the certainty of these findings. Well‐designed, adequately powered trials are needed to determine whether specific hypoxic training protocols or participant characteristics may modulate efficacy in adults with overweight or obesity.

## 1 INTRODUCTION

The prevalence of obesity has risen significantly in recent decades (from 6.7% in 1990 to 11.2% in 2021), globally exceeding underweight and reaching a pandemic burden.^1^ In Italy, 22% of women and 20% of men are obese, with a substantial impact on the healthcare system.^1^, ^2^ Indeed, obesity is associated with a wide range of health issues.^3^ However, cardiometabolic conditions are the leading cause of obesity‐related diseases.^4^, ^5^ Cardiometabolic health, encompassing factors such as cardiovascular function and metabolic processes like glucose homeostasis and lipid metabolism, is crucial for overall health and longevity, especially for adults with obesity.^6^, ^7^ Fasting glucose is strongly associated with both metabolic and cardiovascular diseases.^8^, ^9^, ^10^ A fasting glucose level between 5.6 and 6.9 mmol/L is classified as pre‐diabetes^11^ and according to Huang et al.,^9^ levels above 5.6 mmol/L are associated with an increased risk of cardiovascular diseases.

Given these alarming trends, many exercise interventions have been developed to counter the obesity pandemic.^12^, ^13^ Hypoxic training, which involves exercise in a normobaric (breathing air with a reduced oxygen concentration at constant atmospheric pressure) or hypobaric (breathing air at reduced atmospheric pressure, leading to lower oxygen availability) environment with reduced oxygen levels, appears to be a feasible approach for improving body composition and cardiometabolic health in adults with obesity.^14^, ^15^, ^16^, ^17^, ^18^ Several studies have shown promising results for hypoxic training at improving metabolic profiles and reducing fat mass percentages.^19^, ^20^, ^21^ Moreover, hypoxic training allows participants to train with a reduced mechanical load and stress, specifically in the case of relative hypoxic training, which maintains the same physiological load with reduced mechanical strain.^22^, ^23^ Beyond these biomechanical advantages, hypoxia may further enhance cardiometabolic adaptations through specific molecular pathways. Specifically, low‐frequency intermittent hypoxia, or hypoxic conditioning, directly increases reactive oxygen species, activating pathways that upregulate hypoxia‐inducible factors HIF‐1α and HIF‐1β.^24^ This, in turn, stimulates the production of vascular endothelial growth factor (VEGF), which promotes vascular endothelial genesis and has been associated with improved insulin sensitivity in hypoxic training for adults with obesity.^25^ Concomitantly, HIF‐1‐related signalling influences lipid metabolism by promoting fatty acid oxidation and reducing lipogenesis,^26^ while improved endothelial function and vascular remodelling may contribute to reductions in blood pressure.^27^ These adaptations can potentially translate into improved cardiovascular health and metabolic efficiency.^25^, ^27^ Despite these potential benefits, few reviews have rigorously examined the effects of hypoxic training on body composition,^28^, ^29^ with most lacking selective inclusion criteria and a systematic search strategy. Moreover, the effects on cardiometabolic health have received limited attention. Therefore, the primary aim of this systematic review and meta‐analysis was to compare the effects of exercise training performed under normobaric hypoxia versus normoxia on cardiometabolic health in adults with overweight or obesity. Therefore, we hypothesised that exercise training performed under normobaric hypoxia would lead to greater improvements in fasting glucose compared to equivalent training under normoxia. In line with previous evidence suggesting enhanced endothelial and metabolic adaptations to hypoxia, we further expected potential favourable effects on lipid profile and blood pressure, although these were considered secondary outcomes.

## 2 MATERIALS AND METHODS

The protocol for the systematic review and meta‐analysis was registered in the International Prospective Register of Systematic Reviews (PROSPERO; registration number CRD42024604644). The process adhered to the guidelines outlined in the Cochrane Collaboration Handbook^30^ and other key methodological references appropriate for conducting systematic reviews and meta‐analyses.^31^, ^32^ The reporting of findings followed the Preferred Reporting Items for Systematic Reviews and Meta‐Analyses (PRISMA) guidelines.^33^ The detailed checklist is provided in Table S1. The a priori inclusion and exclusion criteria, following the Population (P), Intervention (I), Comparison (C), Outcomes (O), and Study design (S) (PICOS) framework, are detailed in Table 1.

**TABLE 1:** A priori defined inclusion and exclusion criteria according to the Population (P), Intervention (I), Comparison (C), Outcomes (O), and Study design (S), (PICOS) framework.

Search strategy	Details
Inclusion criteria	P: adults aged 18 to 65 years with overweight or obesity (BMI ≥25 kg/m^2^) and without any psychiatric, cognitive, or other non‐communicable and acute diseases
	I: hypoxic training conducted in normobaric or hypobaric environments with reduced oxygen levels (considering both relative and absolute hypoxia)
	C: normoxic training, where the same exercises are performed at normal oxygen levels (sea level)
	O: metabolic biomarkers, cardiovascular biomarkers
	S: original data (randomised controlled trial)
Exclusion criteria	P: adults with overweight and obesity presenting mental or physical health disorders, physical limitations that prevent physical participation, people who underwent recent bariatric surgery
	I: interventions based on combination between exercise and diet, mixed normoxic‐hypoxic training
	S: qualitative studies, non‐original data (opinion papers, review articles, letters, protocols, commentaries)
Language	English
Time filter	Studies published from the 1990
Database	PubMed/Medline, Web of Science, Cochrane

Abbreviation: BMI, body mass index.

### 2.1 Study selection criteria

Inclusion criteria were as follows: (i) Participants: adults aged 18 to 65 years with overweight or obesity (body mass index [BMI] ≥25 kg/m^2^) and without any psychiatric, cognitive, or other non‐communicable and acute diseases; (ii) Intervention: hypoxic training conducted in normobaric or hypobaric environments with reduced oxygen levels. Hypoxic training involves the use of a generator that reduces oxygen concentration in the air and delivers it through a mask or tent at normal atmospheric pressure; (iii) Comparators: normoxic training, where the same exercises are performed at normal oxygen levels (sea level); (iv) Outcomes: metabolic and cardiovascular outcomes; (v) Study design: randomised controlled trials.

### 2.2 Search strategy

A comprehensive literature search was performed in PubMed‐Medline, Web of Science, and the Cochrane Library up to June 2025. The search focused on studies involving adults and young adults (age range: 18–65) exposed to hypoxic training. Keywords included terms related to body weight AND composition (e.g., “BMI,” “obesity,” “adiposity”) AND metabolic OR cardiovascular outcomes (e.g., “insulin resistance,” “blood pressure,” “lipid profile”). The detailed research string can be found in Data S1 (Table S2). The search was restricted only to studies published in English. Following the predefined selection criteria, two independent reviewers (Alessandro Gatti and Matteo Giuriato) initially screened the titles and abstracts of all identified studies to assess their eligibility. Any disagreements between the reviewers were addressed through discussion, and if consensus was not reached, a third reviewer (Caterina Cavallo) provided the final decision. The full texts of the remaining studies were then retrieved and thoroughly reviewed for final inclusion and data extraction, adhering to the same screening protocol. Additional relevant studies were identified by searching references.

### 2.3 Data extraction and study outcomes

A standardised data extraction protocol and codebook were developed specifically for this systematic review. Two independent authors (Alessandro Gatti and Agnese Pirazzi) conducted data extraction from the final selected studies, with guidance from a third reviewer (Caterina Cavallo) on the inclusion criteria. The primary outcomes of interest included changes in cardiometabolic outcomes, focusing on changes in fasting blood glucose following hypoxic training interventions. Secondary outcomes focused on insulin, homeostatic model assessment for insulin resistance (HOMA‐IR), blood pressure, high‐ and low‐ density lipoprotein (respectively HDL and LDL) and triglycerides. Extracted data included extrinsic variables (author names and year of publication), substantive variables (participant characteristics, type of hypoxic exposure, and training protocols), and methodological variables (study design and duration).

### 2.4 Risk of bias and quality assessments

All studies included in the review were evaluated for methodological quality using the Revised Cochrane Risk‐of‐Bias Tool for Randomized Trials (ROB2). The ROB2 tool assesses the risk of bias across five key domains: (i) bias arising from the randomisation process, (ii) bias due to deviations from intended interventions, (iii) bias due to missing outcome data, (iv) bias in measurement of the outcome, and (v) bias in the selection of the reported result. Each domain was rated as “low risk,” “some concerns,” or “high risk” based on predefined criteria. Assessments were conducted independently by two reviewers (Alessandro Gatti and Caterina Cavallo), with differences resolved through discussion or consultation with a third reviewer (Matteo Giuriato) if necessary.

### 2.5 Statistical analysis

Data from randomised controlled studies were primarily analysed using a standardised mean difference (SMD) approach to assess intervention effects between control and intervention groups. Post‐pre intervention changes were calculated for both control and intervention groups by determining the difference in means from post‐intervention to baseline. A pooled baseline standard deviation was used to standardise the effect sizes across studies. Correction factors were applied to adjust for small sample sizes, ensuring unbiased effect‐size estimates (see Table S3). Effect sizes for both within and between groups differences were calculated as the corrected SMD using a random‐effects model to account for between‐study heterogeneity. The inverse variance method was employed to weight each study's contribution to the pooled effect size. For each outcome, a random‐effects model was fitted using the DerSimonian and Laird's method. Complementary subgroup analyses were conducted based on intervention duration and exercise intensity, while moderation analyses explored the effects of altitude and baseline values. Forest plots were generated to visually represent the pooled SMD and corresponding 95% confidence intervals (CIs), along with prediction intervals using Riley's method to illustrate the range of expected effects in future studies. Heterogeneity among the included studies was assessed using Cochran's Q test and the *I* ^2^ statistic, with *I* ^2^ values categorised as follows: might not be important (0–40%), moderate (30%–60%), substantial (50%–90%), and considerable (75%–100%) heterogeneity. Sensitivity analyses were performed using a leave‐one‐out approach to evaluate the influence of individual studies on pooled estimates. Publication bias was assessed visually through funnel plots and statistically using Egger's regression test. Additionally, influence diagnostics (standardised residuals, Cook's distance, difference in fits, covariance ratios, change in between‐study variance, change in the heterogeneity statistic and study weights) were applied to identify studies having disproportionate influence on model fit or heterogeneity.^34^ For all the analyses a *p*‐value of <0.05 was used to determine statistical significance. All analyses were conducted using R software, version 4.4 (R Foundation for Statistical Computing) and the “metafor” package was used to perform the meta‐analysis. Forest plots were generated to visualise the pooled effect sizes and their corresponding weights across studies.

## 3 RESULTS

**FIGURE 1:**
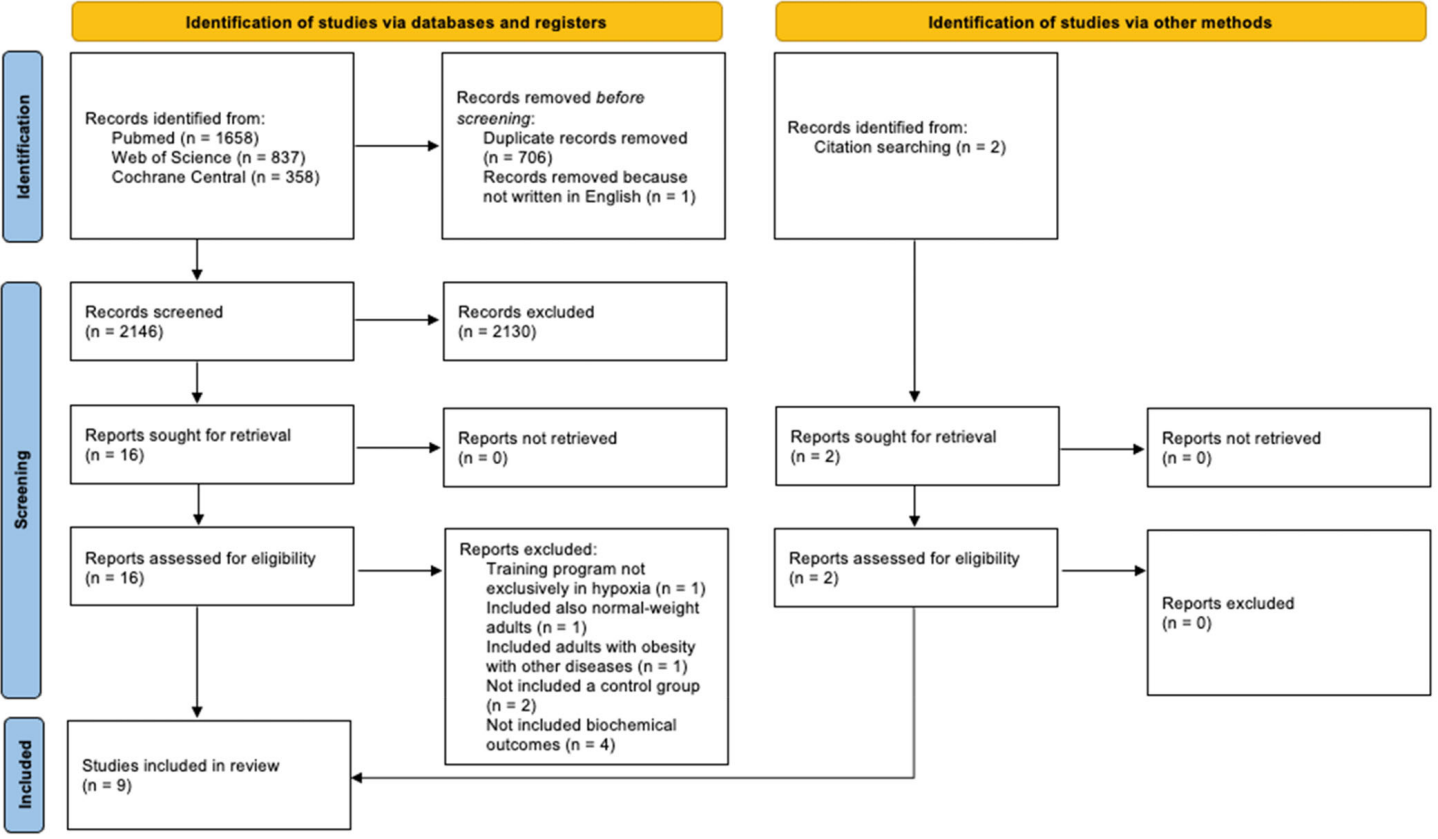
Preferred reporting items for systematic reviews and meta‐analyses (PRISMA) flow diagram.

The systematic search and study selection process is illustrated in the PRISMA flow diagram (Figure 1). Following the removal of duplicate and not in English records, a total of 2146 studies were screened by title and abstract. Of these, 16 full‐text articles were further assessed for eligibility. The primary reasons for excluding studies at this stage were: (i) hypoxic intervention not entirely in a hypoxic environment (one study); (ii) inclusion of adults with obesity and with other known chronic or acute diseases (one study); (iii) interventions solely focused on hypoxic training, without a normoxic control group (two studies); and (iv) not including cardiometabolic outcomes (i.e. fasting glucose, fasting insulin, HOMA‐IR, …) (four studies). Moreover, from checking the reference list of the included studies at this stage, we found two other studies that met the inclusion criteria. Finally, nine studies that met the inclusion criteria were included in the review, and all nine studies were included in the meta‐analyses. One study was considered as two studies for the meta‐analyses because it contained two different protocols both in hypoxia and normoxia (four groups in total).

**FIGURE 2:**
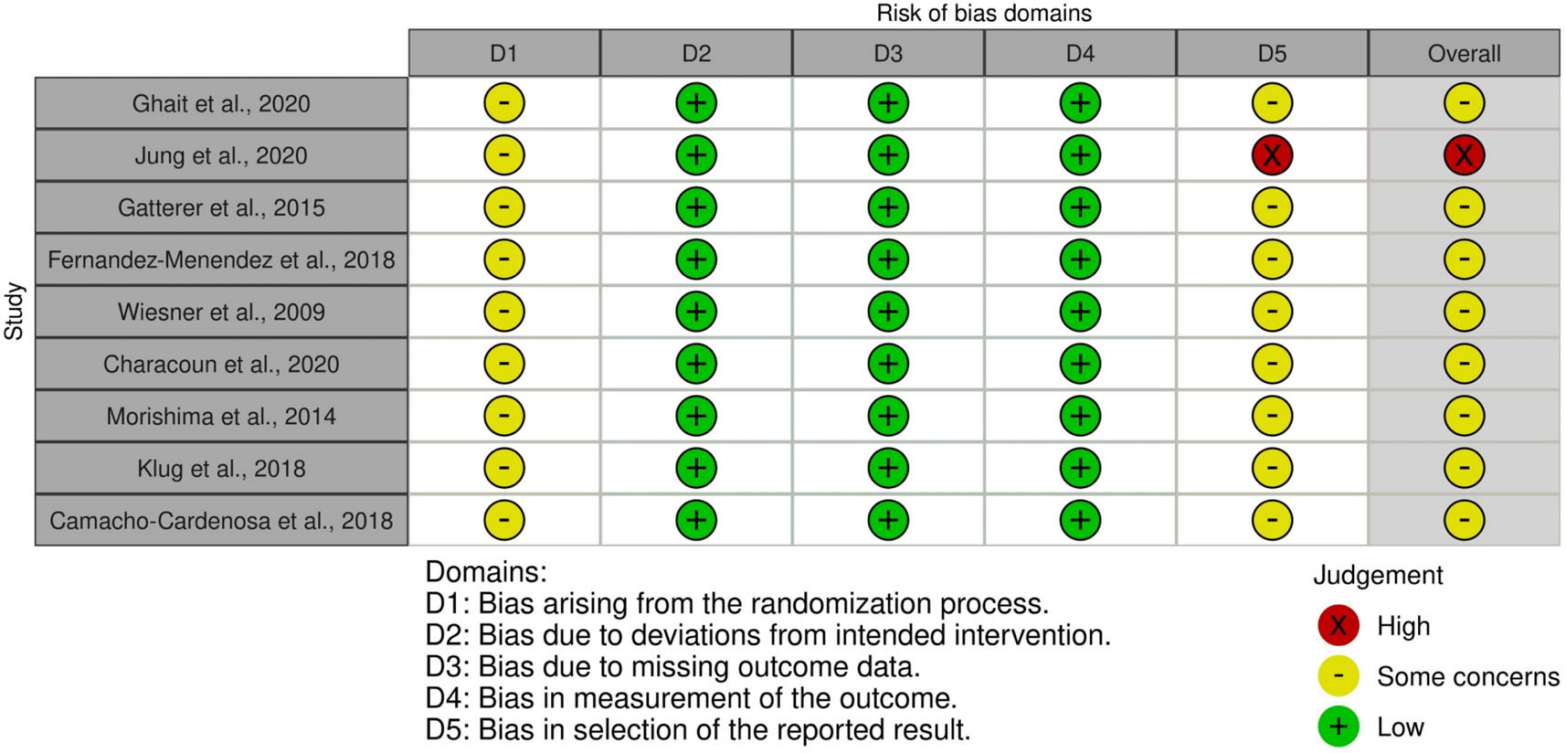
Summary plot of the risk of bias for the included studies, assessed using the Risk of Bias tool 2 (RoB 2).

Risk‐of‐bias assessment for every study is shown in Figure 2, while the overall Risk of Bias is displayed in Figure S1. Overall, the risk of bias has risen “some concerns,” with only one study considered as “high.” Most of the concerns were related to problems in the allocation concealment and in the statistical analyses performed.

A comprehensive summary of the included studies is presented in Table 2 and for the analysed outcomes from Tables S4 to S6. The systematic review and meta‐analysis included a total of 278 participants, with 134 assigned to normoxic training groups and 144 to hypoxic training groups in controlled studies. Over the total systematic review study population 55.9% were women. Included studies' sample sizes ranged from 20 to 45 participants. Mean age was 45 years (SD = 7), with age ranges between 30 and 58 years. Most studies included adults with obesity (*n* = 5) while other studies included both people with overweight and obesity (*n* = 5). Exercise modalities varied across studies, including cycling (4 studies, 136 participants), walking (4 studies, 91 participants), mixed (1 study, 32 participants) and pilates (1 study, 22 participants). In terms of training protocols, studies' interventions considered high‐intensity (3 studies, 90 participants), low intensity (2 studies, 45 participants) and moderate intensity (5 studies, 146 participants). Most of the studies used a moderate altitude stimulus (2000 to 3000 m simulated altitude; 7 studies; 69 intervention group participants), while the remaining studies used a high‐altitude stimulus (3000 to 5500 m simulated altitude; 3 studies; 46 intervention group participants).^41^

**TABLE 2:** Characteristics of included studies.

Study	Group	Participants characteristics				Intervention design						
		Sample size	Age (years)	Weight (kg)	BMI (kg/m^2^)	Type of exposure	Type of training	Protocol	Volume	Intensity	Hypoxic load	Altitude (m)
Ghait et al.^19^	Normoxia	13 (M) 2 (W)	52 ± 7.5	99.9 ± 15.5	32.4 ± 4.8	None	Aerobic	HIIT; cycling at 80% or 100% at maximal workload	3 session per week of 40 min; 8 weeks	High	‐	Sea level
	Hypoxia	10 (M) 6 (W)	51 ± 8.3	95.4 ± 19.4	31.5 ± 4.0	Active, normobaric	Aerobic	HIIT; cycling at 80% or 100% at maximal workload	3 session per week of 40 min; 8 weeks	High	Relative	4200
Jung et al.^35^	Normoxia	10 (W)	43.8 ± 8.6	66.3 ± 11	25.1 ± 3.3	None	Pilates	Pilates training using a tubing band	3 session per week of 50 min; 12 weeks	Low	‐	Sea level
	Hypoxia	12 (W)	47.2 ± 6.4	68.0 ± 10.1	27.1 ± 4.3	Active, normobaric	Pilates	Pilates training using a tubing band	3 session per week of 50 min; 12 weeks	Low	Not specified	3000
Gatterer et al.^36^	Normoxia	6 (M) 10 (W)	52.4 ± 7.9	103.2 ± 15.11	36.3 ± 4.0	None	Aerobic	65%–70% of the maximal heart rate using a treadmill, cycle ergometer or a cross trainer	2 session per week of 90 min; 32 weeks	Moderate	‐	Sea level
	Hypoxia	4 (M) 12 (W)	50.3 ± 10.3	105.5 ± 20.0	37.9 ± 8.1	Active + passive, normobaric	Aerobic	65%–70% of the maximal heart rate using a treadmill, cycle ergometer or a cross trainer	2 session per week of 90 min; 32 weeks	Moderate	Relative	3500
Fernandez‐Menendez et al.^37^	Normoxia	2 (M) 9 (W)	32.2 ± 8.4	96.6 ± 9.6	32.9 ± 2.7	None	Aerobic	Walking on a treadmill at each individual's preferred walking speed	3 session per week of 60 min; 3 weeks	Low	‐	Sea level
	Hypoxia	2 (M) 10 (W)	34.8 ± 4.7	96.8 ± 9.5	34.1 ± 2.6	Active, normobaric	Aerobic	Walking on a treadmill at each individual's preferred walking speed	3 session per week of 60 min; 3 weeks	Low	Not specified	3000
Wiesner et al., 2009^21^	Normoxia	8 (M) 13 (W)	42.1 ± 1.7	87.5 ± 3.6	32.5 ± 0.8	None	Aerobic	Walking on a treadmill at 65% of maximum oxygen consumption	3 session per week of 60 min; 4 weeks	Moderate	‐	Sea level
	Hypoxia	10 (M) 14 (W)	42.2 ± 1.2	93.4 ± 2.6	33.1 ± 0.3	Active, normobaric	Aerobic	Walking on a treadmill at 65% of maximum oxygen consumption	3 session per week of 60 min; 4 weeks	Moderate	Relative	2740
Chacaroun et al.^38^	Normoxia	8 (M) 4 (W)	56 ± 11	Not reported	31.8 ± 3.2	None	Aerobic	Cycling at 75 ± 3% of the maximal heart rate	3 session per week of 45 min; 8 weeks	High	‐	Sea level
	Hypoxia	11 (M) 3 (W)	52 ± 12	Not reported	31.2 ± 2.4	Active, normobaric	Aerobic	Cycling at 75% ± 3% of the maximal heart rate	3 session per week of 45 min; 8 weeks	High	Relative	3700
Morishima et al.^39^	Normoxia	11 (M)	30 ± 2	73.8 ± 4	25.4 ± 0.9	None	Aerobic	Cycling at 55% of the maximal oxygen uptake	3 session per week of 60 min; 4 weeks	Moderate	‐	Sea level
	Hypoxia	9 (M)	32 ± 3	74.4 ± 4.2	25.6 ± 1.2	Active, normobaric	Aerobic	Cycling at 55% of the maximal oxygen uptake	3 session per week of 60 min; 4 weeks	Moderate	Relative	2500
Klug et al.^40^	Normoxia	11 (M)	57.6 ± 2.2	108.5 ± 3	34.1 ± 0.9	None	Aerobic	Walking on a treadmill at 50%–60% of their individual maximal heart rate	3 session per week of 60 min; 6 weeks	Moderate	‐	Sea level
	Hypoxia	12 (M)	55.0 ± 2.1	109.1 ± 5.2	35.5 ± 1.4	Active, normobaric	Aerobic	Walking on a treadmill at 50%–60% of their individual maximal heart rate	3 session per week of 60 min; 6 weeks	Moderate	Relative	2500
Camacho‐Cardenosa et al., 2018a^20^	Normoxia	13 (W)	43.14 ± 7.67	80.41 ± 16.27	29.59 ± 5.25	None	Aerobic	HIIT using a cycle ergometer at 90% Wmax	3 session per week of 60 min; 12 weeks	Moderate	‐	Sea level
	Hypoxia	13 (W)	44.43 ± 7.18	80.1 ± 18.88	30.03 ± 6.37	Active, normobaric	Aerobic	HIIT using a cycle ergometer at 90% Wmax	3 session per week of 60 min; 12 weeks	Moderate	Absolute	2500
Camacho‐Cardenosa et al., 2018b^20^	Normoxia	15 (W)	40.05 ± 8.66	77.94 ± 11.31	28.74 ± 4.77	None	Aerobic	HIIT using a cycle ergometer at 130% Wmax	3 session per week of 60 min; 12 weeks	High	‐	Sea level
	Hypoxia	18 (W)	37.4 ± 10.25	73.73 ± 11.11	27.71 ± 4.55	Active, normobaric	Aerobic	HIIT using a cycle ergometer at 130% Wmax	3 session per week of 60 min; 12 weeks	High	Absolute	2500

*Note*: Camacho‐Cardenosa et al., 2018a and Camacho‐Cardenosa et al., 2018b are data from the same study but using four different groups.

Abbreviations: BMI, body mass index; HIIT: high‐intensity interval training; M, men; W, women; Wmax: maximal power.

**FIGURE 3:**
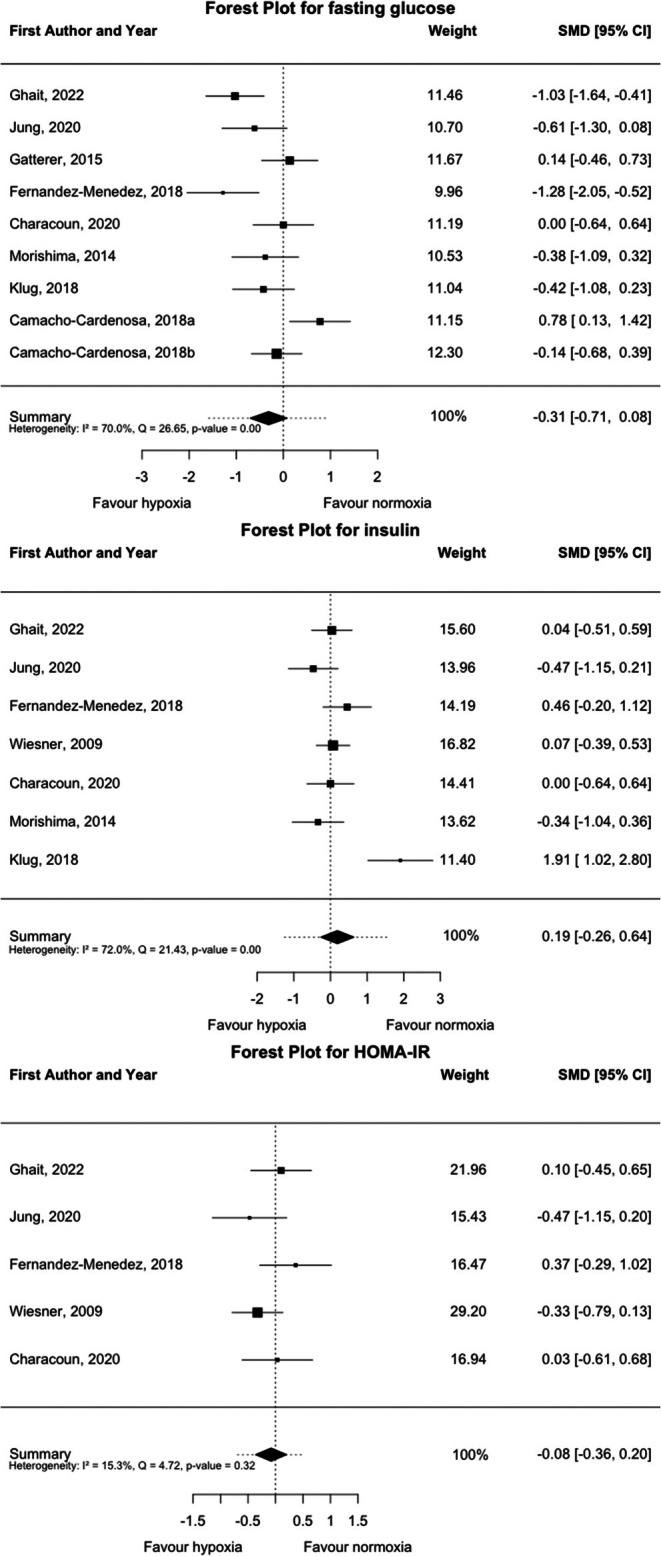
Forest plot of standardised mean differences (estimate) for fasting glucose, fasting insulin and homeostatic model assessment for insulin resistance (HOMA‐IR). A negative value indicates a greater reduction in outcome after hypoxic interventions than after normoxic interventions, whereas a positive value indicates a smaller reduction in outcome after hypoxic interventions than after normoxic interventions. CI, confidence interval; SMD, standardised mean difference.

The effect of the hypoxic training on metabolic biomarkers is shown in Figure 3 (nine studies; 233 participants). Fasting glucose and fasting insulin did not change significantly in the hypoxic compared to the normoxic interventions (fasting glucose; SMD = −0.31, 95% CI = −0.71, 0.08, *p* = 0.118 and fasting insulin; SMD = 0.19, 95% CI = −0.26, 0.64, *p* = 0.415). However, we report high heterogeneity for both fasting glucose and insulin (fasting glucose; Q (df = 8) = 26.65, *p* < 0.001; *I* ^2^ = 69.98%; and fasting insulin; Q (df = 7) = 21.43, *p* = 0.415; *I* ^2^ = 72.00%). Post‐pre changes in HOMA‐IR did not differ significantly between the two interventions (SMD = −0.08, 95% CI = −0.36, 0.20, *p* = 0.579) without significant heterogeneity (Q (df = 4) = 4.72, *p* = 0.317; *I* ^2^ = 15.26%). However, when analysing the post‐pre changes, hypoxic training significantly reduced fasting glucose (SMD = −0.24, 95% CI = −0.46, −0.03, *p* = 0.028), while normoxic significantly reduced insulin levels after the intervention (SMD = −0.59, 95% CI = −1.16, −0.03, *p* = 0.046) (Figure S2). Leave‐one‐out sensitivity analyses for fasting glucose, insulin, and HOMA‐IR (Figures S6–S8) reported stable overall estimates, as the exclusion of individual studies did not affect the pooled effects, indicating that no single study disproportionately influenced the results. Funnel plots (Figures S15–S17) showed a symmetrical distribution of studies, and Egger's tests were nonsignificant (*p* > 0.05), suggesting no evidence of publication bias. Influence diagnostics (Figure S24–S26) showed that the model was generally robust. Standardised residuals, Cook's distances, and leverage values identified that only one study had a moderate influence on the overall estimates for both insulin^40^ and HOMA‐IR.^21^ However, its influence did not alter the pooled effect size, supporting the stability of the meta‐analytic results. Subgroup and moderation analyses (Tables S7–S9) showed that intervention duration, intensity, and baseline levels influenced the between‐group post‐pre differences in metabolic responses to hypoxic training. Programs lasting less than 8 weeks had a significantly greater reduction in fasting glucose in the hypoxia group compared with normoxia (SMD = −0.67, 95% CI = −1.22 to −0.12, *p* = 0.016), whereas interventions exceeding 8 weeks showed no significant between‐group difference (*p* = 0.569). Likewise, low‐intensity protocols resulted in a larger decrease in fasting glucose under hypoxia (SMD = −0.93, 95% CI = −1.58 to −0.27, *p* = 0.006), while moderate‐ and high‐intensity interventions showed no significant effects (*p* > 0.05). Altitude did not significantly moderate any outcome (*p* > 0.05), but baseline levels were significant moderators for fasting glucose (*p* = 0.020) and fasting insulin (*p* < 0.001), indicating that participants with higher initial values experienced greater hypoxia‐induced improvements.

**FIGURE 4:**
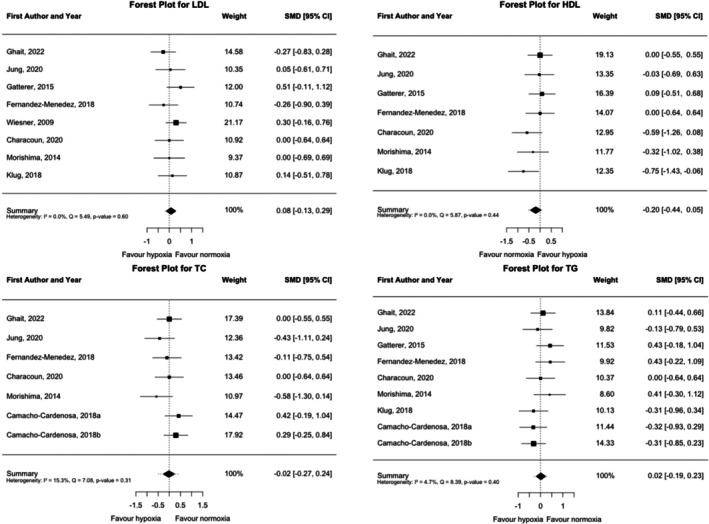
Forest plot of standardised mean differences (estimate) for low‐density and high‐density lipoprotein (HDL and LDL), total cholesterol (TC) and triglycerides (TG). A negative value indicates a greater reduction in outcome after hypoxic interventions than after normoxic interventions, whereas a positive value indicates a smaller reduction in outcome after hypoxic interventions than after normoxic interventions. CI, confidence interval; SMD, standardised mean difference.

The effect of the hypoxic training on lipid profile biomarkers is shown in Figure 4. LDL, HDL, total cholesterol (TC) and triglycerides (TG) did not change significantly in the hypoxic compared to the normoxic interventions (LDL; SMD = −0.20, 95% CI = −0.44, 0.05, *p* = 0.473; HDL; SMD = 0.08, 95% CI = −0.13, 0.29, *p* = 0.112; TC; SMD = −0.02, 95% CI = −0.27, 0.24, *p* = 0.892; and TG; SMD = 0.02, 95% CI = −0.19, 0.23, *p* = 0.874) without having high heterogeneity (LDL; Q (df = 7) = 5.49, *p* = 0.599; *I* ^2^ = 0.00%; HDL; Q (df = 6) = 5.87, *p* = 0.438; *I* ^2^ = 0.00%; TC; Q (df = 6) = 7.08, *p* = 0.313; *I* ^2^ = 15.29%; and TG; Q (df = 8) = 8.29, *p* = 0.396; *I* ^2^ = 4.67%). When analysing the post‐pre differences, both training methods reduced the LDL levels (hypoxia: SMD = −0.26, 95% CI = −0.48, 0.05, *p* = 0.018; normoxia: SMD = −0.31, 95% CI = −0.53, −0.09, *p* = 0.007). However, while hypoxia reduced the HDL levels (SMD = −0.27, 95% CI = −0.53, −0.01, *p* = 0.043), normoxic training reduced the TG levels (SMD = −0.41, 95% CI = −0.76, −0.05, *p* = 0.026) (Figures S3 and S4). Sensitivity, publication bias, and influence diagnostics for lipid profile outcomes (Figures S9–S12, S18–S20, and S27–S30) confirmed the robustness of the between‐group post‐pre differences. Leave‐one‐out sensitivity analyses showed that the exclusion of any single study did not substantially alter the pooled estimates for LDL‐C, HDL‐C, total cholesterol, or triglycerides. Funnel plots appeared symmetrical, and Egger's tests revealed no significant publication bias for LDL‐C (*p* = 0.460), HDL‐C (*p* = 0.102) and triglycerides (*p* = 0.332), though for total cholesterol Egger's value was significant (*p* = 0.0497). Influence diagnostics further supported model stability, as standardised residuals, Cook's distances, and leverage statistics showed no outliers or studies that disproportionately influenced heterogeneity or overall model fit. Subgroup and moderation analyses for lipid outcomes (Tables S7–S9) indicated that neither intervention duration, exercise intensity, nor altitude significantly influenced the between‐group post‐pre differences. Programs lasting less than 8 weeks and those exceeding 8 weeks showed no significant effects on LDL‐C, HDL‐C, total cholesterol, or triglycerides (all *p* > 0.05). Similarly, low‐, moderate‐, and high‐intensity interventions showed no significant between‐group differences (all *p* > 0.05). Moderation analysis revealed no significant effects of altitude (*p* > 0.05) or baseline lipid levels (*p* > 0.05).

**FIGURE 5:**
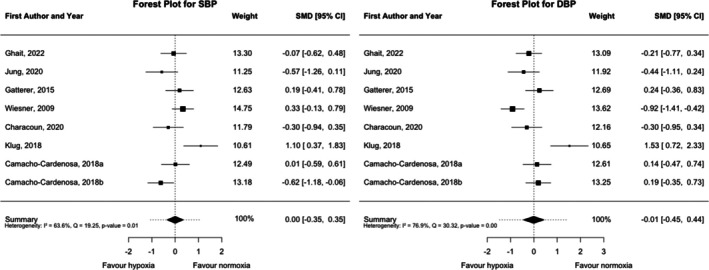
Forest plot of standardised mean differences (estimate) for systolic and diastolic blood pressure (SBP and DBP). A negative value indicates a greater reduction in outcome after hypoxic interventions than after normoxic interventions, whereas a positive value indicates a smaller reduction in outcome after hypoxic interventions than after normoxic interventions. CI, confidence interval; SMD, standardised mean difference.

The effect of the hypoxic training on blood pressure is shown in Figure 5. Post‐pre changes in systolic (SBP) and diastolic blood pressure (DBP) did not differ significantly between the two interventions (SBP; SMD = 0.00, 95% CI = −0.35, 0.35, *p* = 0.995; and DBP SMD = −0.01, 95% CI = −0.45, 0.44, *p* = 0.976) with significant heterogeneity for both outcomes (SBP; Q (df = 7) = 19.25, *p* = 0.007; *I* ^2^ = 63.64%; and DBP; Q (df = 7) = 30.32, *p* < 0.001; *I* ^2^ = 76.92%). However, when analysing the single intervention effect, while normoxic intervention only reduced DBP (SMD = −0.29, 95% CI = −0.51, −0.07, *p* = 0.010), hypoxia reduced both SBP and DBP (SBP; SMD = −0.44, 95% CI = −0.68, −0.20, *p* < 0.001; and DBP SMD = −0.45, 95% CI = −0.78, −0.11, *p* = 0.009) (Figure S5). Sensitivity, publication bias, and influence diagnostics (Figures S13, S14, S22, S23, and S31, S32) confirmed the robustness of the between‐group post‐pre differences in blood pressure outcomes. Leave‐one‐out and influence analyses showed that no individual study greatly affected the pooled estimates for SBP or DBP, while Egger's tests indicated no publication bias for SBP (*p* = 0.723) while it was significant for DBP (*p* = 0.0131). Subgroup and moderation analyses (Tables S7–S9) suggested limited influence of intervention characteristics. Shorter (<8 weeks) and moderate‐ to high‐intensity interventions showed borderline effects on SBP, whereas DBP remained unchanged (*p* > 0.05). Altitude moderated SBP responses (*p* = 0.024), and higher baseline SBP predicted greater reductions (*p* < 0.001).

## 4 DISCUSSION

The primary aim of this study was to review the effects of normobaric hypoxic training on cardiometabolic outcomes, with fasting glucose as the main outcome. Secondary outcomes, including insulin levels, HOMA‐IR, BP, lipid profile, were also analysed. We also analysed within‐group pre–post changes to assess the effect of each intervention.

Overall, our analyses did not reveal significant differences between normobaric hypoxic interventions when compared to those performed under normoxic conditions. Outcome‐specific trends favoured one condition or the other, as explored in subgroup and moderation analyses. Hypoxic training therefore appears to provide comparable cardiometabolic benefits to normoxic training in adults with overweight or obesity.

### 4.1 The effects of normobaric hypoxic training on glucose homeostasis

On fasting glucose, we did not find any differences between the effects of training interventions performed under hypoxic and normoxic conditions. Subgroup analyses suggested that shorter and lower‐intensity hypoxic protocols may be more effective for fasting glucose. This may be due to heterogeneity in baseline glucose and training characteristics. This aligns with our moderation analysis, which identified baseline fasting glucose as a significant predictor of the response to hypoxic training, suggesting greater improvements in participants with higher baseline values. While some studies found a higher reduction in fasting glucose for hypoxic interventions, most of the studies found no differences.^19^, ^37^ Conversely, Camacho‐Cardenosa et al.^20^ found a greater reduction for the normoxic interventions. Even if there were no differences between hypoxia and normoxia, when considering within‐group changes, only the hypoxic training led to a reduced fasting glucose after the intervention. This should be considered a crucial marker for the training interventions, since it has been related to several cardiometabolic diseases as mentioned in the introduction.^42^, ^43^

Our findings regarding insulin levels did not reveal significant differences between normobaric hypoxic and normoxic conditions. Similarly, insulin levels did not differ between conditions. Nonetheless, when comparing pre‐ and post‐interventions results, normoxic training led to a slight significant improvement in insulin levels. Moreover, our moderation results, showed that baseline insulin values significantly moderated the changes, revealing larger improvements among participants with higher initial insulin levels.

No clear differences were observed for HOMA‐IR. Overall, most studies reported that all the training interventions led to improvements in the general health of the targeted population. The greater fasting glucose response under hypoxia may relate to HIF‐1–mediated metabolic effects, as previously mentioned in the introduction.^25^ However, the findings do not clearly indicate which condition was more effective in enhancing these outcomes, especially regarding the HOMA‐IR marker. However, the limited number of HOMA‐IR studies restricts interpretation. In addition, differences in participants' baseline glucose and insulin levels may have influenced the magnitude of the observed effects. Future studies should focus specifically on adults with impaired glucose homeostasis to understand whether hypoxic training could be a valid alternative to normoxic training.

### 4.2 The effects of normobaric hypoxic training on lipid profile

Most studies reported lipid profile improvements, with no significant differences between conditions. Subgroup and moderation analyses did not identify moderators of the between‐group effects. Within‐group analyses showed that both conditions reduced LDL. These within‐group effects suggest that both training modalities were effective in improving lipid metabolism independently of environmental condition. Sensitivity, publication bias, and influence diagnostics further confirmed the stability of these findings, showing no evidence of outlier studies or publication bias across lipid profile outcomes. Some studies, such as those by Jung et al.^35^ and Klug et al.,^40^ reported a greater improvement in lipid profile following hypoxic training. In contrast, Wiesner et al.^21^ and Chacaroun et al.^38^ did not observe a similar trend when comparing the two conditions.

Notably, our findings revealed a significant reduction in HDL levels following normobaric hypoxic training. This might have influenced the results of some studies reporting a greater trend of improvement in TC levels after normobaric hypoxic interventions. The changes in total cholesterol are influenced by variations in both LDL and HDL; a reduction in either parameter contributes to an overall decline in TC. However, despite the decrease observed in HDL levels, the values remained within healthy thresholds following the exposure to normobaric hypoxic training. Our results underline, that even if with a reduced mechanical load, hypoxic training might be able to improve the lipid profile of adults with overweight and obesity, achieving similar benefits on lipid profile as the normoxic training. Future studies should assess whether absolute hypoxic training (same physiological load but higher mechanical load) could further enhance lipid profile improvements compared to normoxic training. Future research should also explore absolute hypoxic training and long‐term effects on lipid metabolism.

### 4.3 The effects of normobaric hypoxic training on blood pressure

Our findings did not reveal significant differences between the two types of training. Subgroup analyses showed no influence of duration, intensity, or baseline levels on between‐group changes. Borderline effects were seen only for systolic pressure in shorter or higher‐intensity protocols. When analysing within‐group changes, hypoxic training reduced both SBP and DBP, while normoxia mainly affected DBP. A decrease in both SBP and DBP can be considered a target for training interventions, as it is associated with a reduced risk of cardiovascular diseases.^44^ However, we should consider, especially for the comparison between the two training protocols, a high heterogeneity of the results. Sensitivity and bias analyses confirmed result stability, indicating that the observed variability was not driven by any single study or publication bias. While Camacho‐Cardenosa et al.^20^ with a training performed in absolute hypoxia, reported a trend towards greater BP improvements following interventions performed under normobaric hypoxia, Klug et al.^40^ observed a greater extent of improvement following the training intervention under normoxic conditions. This variability aligns with our moderation analysis, which revealed that altitude influenced systolic responses, suggesting slightly greater reductions at higher simulated elevations, while higher baseline systolic values strongly predicted larger training‐induced decreases.

This improvement may relate to hypoxia‐induced endothelial adaptations.^24^ Nevertheless, based on our results, it remains unclear whether hypoxic interventions improve BP; future studies should consider training in absolute intensity hypoxia since it was the only study that found improvements in BP. As discussed above for glucose outcomes, part of the variability in BP responses likely stems from differences in participants' baseline blood pressure (Table S7). Indeed, while some studies included participants with normal blood pressure, others included individuals with hypertension. Future studies should focus specifically on adults with high blood pressure to determine whether hypoxic training could be a viable alternative to normoxic training for blood pressure management.

### 4.4 Practical implications

Our systematic review and meta‐analysis found that hypoxic training produced similar benefits to normoxic training in adults with overweight or obesity. However, the current literature lacks standardisation in hypoxic training protocols. Specifically, studies differed in intervention duration, training type, and simulated altitude. Most employed relative‐intensity hypoxic protocols that matched physiological effort to normoxic training conditions.^19^, ^20^, ^21^ Additionally, some studies included in this systematic review and meta‐analysis exhibited minor methodological limitations, likely due to the novelty of the topic, which may have influenced our findings. However, sensitivity and influence diagnostics indicated that no single study disproportionately affected the overall estimates, suggesting that these methodological differences did not substantially bias the pooled results.

We acknowledge that our study has some limitations. Firstly, the lack of stratification based on overweight and obesity status might have provided additional information on hypoxic training. However, a subgroup meta‐analysis could not be performed because the included studies reported combined data for participants with overweight and obesity, without providing separate values for each group. In addition, although we have performed publication bias analyses, we should also consider the bias due to the small number of studies available for some outcomes (e.g., HOMA‐IR). Moreover, since we have considered only studies written in English this could have led to language bias. Finally, the limited literature on this topic prevented us from isolating the effects of relative or absolute hypoxic training.

Our study presents also several strengths. One of the strengths of this systematic review and meta‐analysis is the inclusion of a substantial number of studies, despite the stringent inclusion and exclusion criteria and the novelty of the topic. Moreover, this is the first systematic review and meta‐analysis analysing the impact of hypoxic training on cardiometabolic health in adults with overweight and obesity. In addition, we performed within‐groups comparisons to understand whether the training protocols performed were effective on cardiometabolic health.

## 5 CONCLUSIONS

This meta‐analysis found no significant difference between the effectiveness of hypoxic and normoxic training on cardiometabolic markers in adults with overweight and obesity. However, significant heterogeneity in the results makes a definitive interpretation difficult. Future studies with standardised protocols focusing on specific subgroups are needed to clarify the potential benefits of hypoxic training.

## CONFLICT OF INTEREST STATEMENT

The authors declare no conflicts of interest.

## Supporting information


**Data S1:** Supplementary information.

## Data Availability

The data that support the findings of this study are available from the corresponding author upon reasonable request.
