# Supplementary material for: Effects of hypoxic training interventions on cardiometabolic health of adults with overweight and obesity: A systematic review and meta‐analysis
Source: Diabetes Obes Metab. 2025 Nov 18;28(2):865–77. doi: 10.1111/dom.70303 (PMC12803568; doi:10.1111/dom.70303)
Supplement: Supplementary file 1 — Data S1: Supplementary information. [file DOM-28-865-s001.docx]

**Supplementary materials**

Index

[Figure S1. Summary plot of the risk of bias for the included studies, assessed using the Risk of Bias tool 2 (RoB 2). 5](#_Toc211240170)

[Figure S2. Forest plot of standardized mean differences within groups (post-pre estimates) for fasting glucose, fasting insulin and homeostatic model assessment for insulin resistance (HOMA-IR). A negative value indicates a reduction in outcome after the intervention, whereas a positive value indicates an improvement in outcome after the intervention. Abbreviations: CI, confidence interval; SMD, standardized mean difference. 6](#_Toc211240171)

[Figure S3. Forest plot of standardized mean differences within groups (post-pre estimates) for low-density and high-density lipoprotein (LDL and HDL). A negative value indicates a reduction in outcome after the intervention, whereas a positive value indicates an improvement in outcome after the intervention. Abbreviations: CI, confidence interval; SMD, standardized mean difference. 9](#_Toc211240172)

[Figure S4. Leave-one-out sensitivity analysis on the pooled allometric exponent of height and cardiorespiratory fitness (CRF) in boys. 11](#_Toc211240173)

[Figure S5. Forest plot of standardized mean differences within groups (post-pre estimates) for systolic and diastolic blood pressure (SBP and DBP). A negative value indicates a reduction in outcome after the intervention, whereas a positive value indicates an improvement in outcome after the intervention. Abbreviations: CI, confidence interval; SMD, standardized mean difference. 13](#_Toc211240174)

[Figure S6. Leave-one-out sensitivity analysis on the post-pre changes for fasting glucose. 14](#_Toc211240175)

[Figure S7. Leave-one-out sensitivity analysis on the post-pre changes for fasting insulin. 15](#_Toc211240176)

[Figure S8. Leave-one-out sensitivity analysis on the post-pre changes for homeostatic model assessment of insulin resistance (HOMA-IR). 16](#_Toc211240177)

[Figure S9. Leave-one-out sensitivity analysis on the post-pre changes for low-density lipoprotein (LDL-C). 17](#_Toc211240178)

[Figure S10. Leave-one-out sensitivity analysis on the post-pre changes for high-density lipoprotein (HDL-C). 18](#_Toc211240179)

[Figure S11. Leave-one-out sensitivity analysis on the post-pre changes for total cholesterol. 19](#_Toc211240180)

[Figure S12. Leave-one-out sensitivity analysis on the post-pre changes for triglycerides. 20](#_Toc211240181)

[Figure S13. Leave-one-out sensitivity analysis on the post-pre changes for systolic blood pressure (SBP). 21](#_Toc211240182)

[Figure S14. Leave-one-out sensitivity analysis on the post-pre changes for diastolic blood pressure (DBP). 22](#_Toc211240183)

[Figure S15. Funnel plot of the meta-analysis showing observed change versus standard error across studies of fasting glucose for post-pre changes between the two groups. 23](#_Toc211240184)

[Figure S16. Funnel plot of the meta-analysis showing observed change versus standard error across studies of fasting insulin for post-pre changes between the two groups. 24](#_Toc211240185)

[Figure S17. Funnel plot of the meta-analysis showing observed change versus standard error across studies of homeostatic model assessment of insulin resistance (HOMA-IR) for post-pre changes between the two groups. 25](#_Toc211240186)

[Figure S18. Funnel plot of the meta-analysis showing observed change versus standard error across studies of low-density lipoprotein (LDL-C) for post-pre changes between the two groups. 26](#_Toc211240187)

[Figure S19. Funnel plot of the meta-analysis showing observed change versus standard error across studies of high-density lipoprotein (HDL-C) for post-pre changes between the two groups. 27](#_Toc211240188)

[Figure S20. Funnel plot of the meta-analysis showing observed change versus standard error across studies of total cholesterol for post-pre changes between the two groups. 28](#_Toc211240189)

[Figure S21. Funnel plot of the meta-analysis showing observed change versus standard error across studies of triglycerides for post-pre changes between the two groups. 29](#_Toc211240190)

[Figure S22. Funnel plot of the meta-analysis showing observed change versus standard error across studies of systolic blood pressure for post-pre changes between the two groups. 30](#_Toc211240191)

[Figure S23. Funnel plot of the meta-analysis showing observed change versus standard error across studies of diastolic blood pressure for post-pre changes between the two groups. 31](#_Toc211240192)

[Figure S24. Influence diagnostics for the meta-analysis of post-pre changes between groups for fasting glucose. 32](#_Toc211240193)

[Figure S25. Influence diagnostics for the meta-analysis of post-pre changes between groups for fasting insulin. 33](#_Toc211240194)

[Figure S26. Influence diagnostics for the meta-analysis of post-pre changes between groups for homeostatic model assessment of insulin resistance. 34](#_Toc211240195)

[Figure S27. Influence diagnostics for the meta-analysis of post-pre changes between groups for low-density lipoprotein (LDL-C). 35](#_Toc211240196)

[Figure S28. Influence diagnostics for the meta-analysis of post-pre changes between groups for high-density lipoprotein (HDL-C). 36](#_Toc211240197)

[Figure S29. Influence diagnostics for the meta-analysis of post-pre changes between groups for total cholesterol. 37](#_Toc211240198)

[Figure S30 Influence diagnostics for the meta-analysis of post-pre changes between groups for triglycerides. 38](#_Toc211240199)

[Figure S31. Influence diagnostics for the meta-analysis of weight exponents for post-pre changes between groups for systolic blood pressure (SBP). 39](#_Toc211240200)

[Figure S32. Influence diagnostics for the meta-analysis of weight exponents for post-pre changes between groups for diastolic blood pressure (DBP). 40](#_Toc211240201)

[Table S1. PRISMA checklist. 41](#_Toc211240202)

[Table S2. Search strategy employed for each database. 43](#_Toc211240203)

[Table S3. Equations used for the calculation of effect sizes. 44](#_Toc211240204)

[Table S4. Pre and post interventions values of glucose homeostasis of the studies included in the meta-analysis. 45](#_Toc211240205)

[Table S5. Pre and post interventions values of lipids profile of the studies included in the meta-analysis. 46](#_Toc211240206)

[Table S6. Pre and post interventions values of blood pressure of the studies included in the meta-analysis. 48](#_Toc211240207)

[Table S7. Moderation analysis for all the outcomes considering altitude and the baseline level. 49](#_Toc211240208)

[Table S8. Subgroup analysis for all the outcomes considering intervention duration. 50](#_Toc211240209)

[Table S9. Subgroup analysis for all the outcomes considering intensity of the exercise. 51](#_Toc211240210)

**
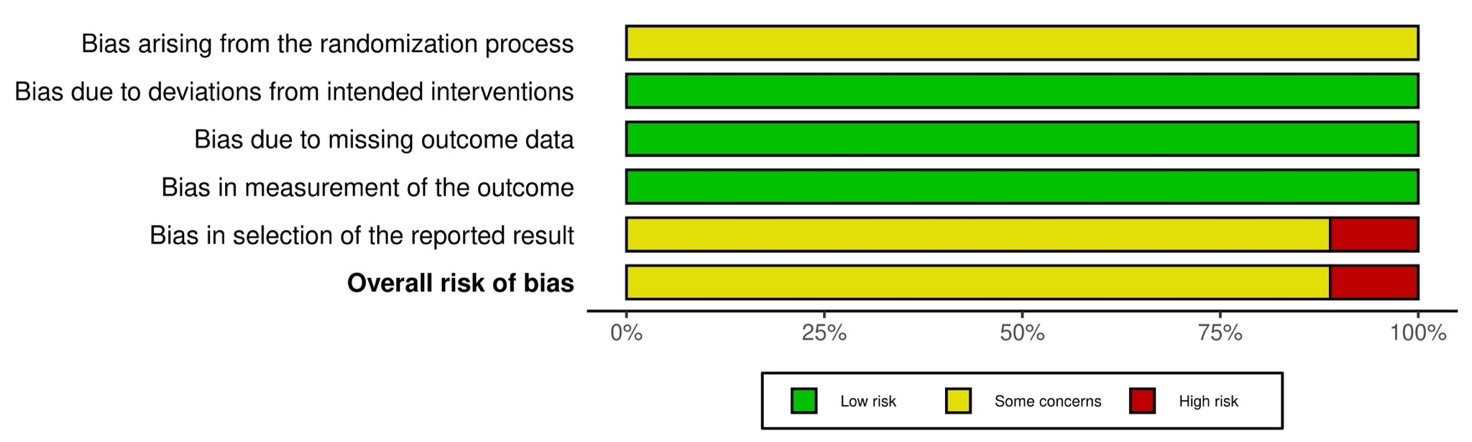
**

# Figure S1. Summary plot of the risk of bias for the included studies, assessed using the Risk of Bias tool 2 (RoB 2).


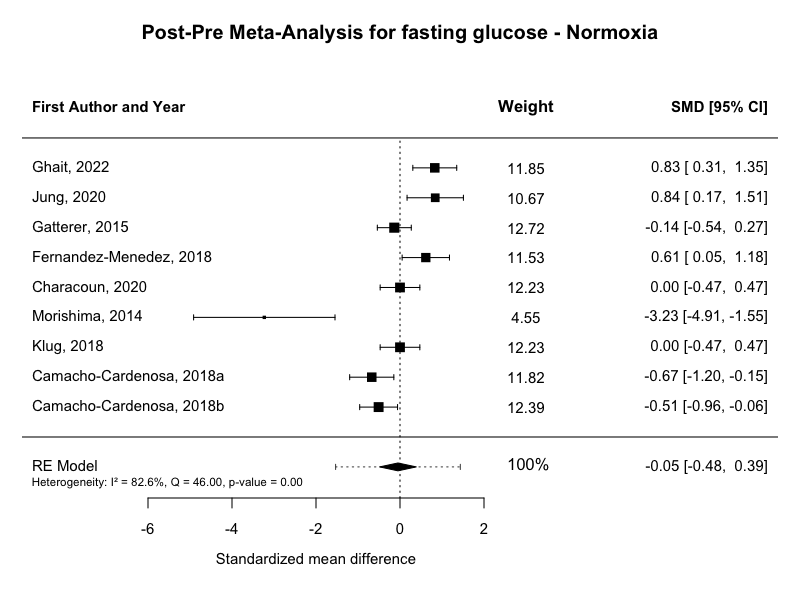

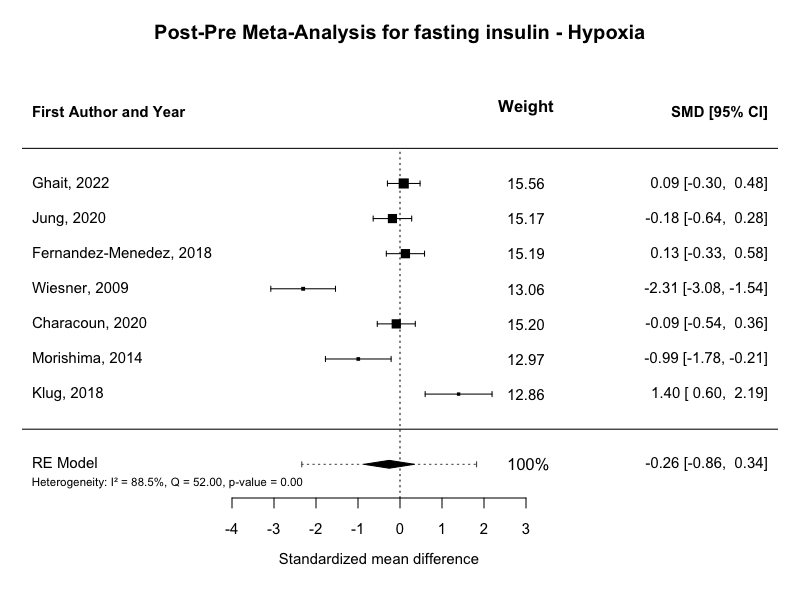

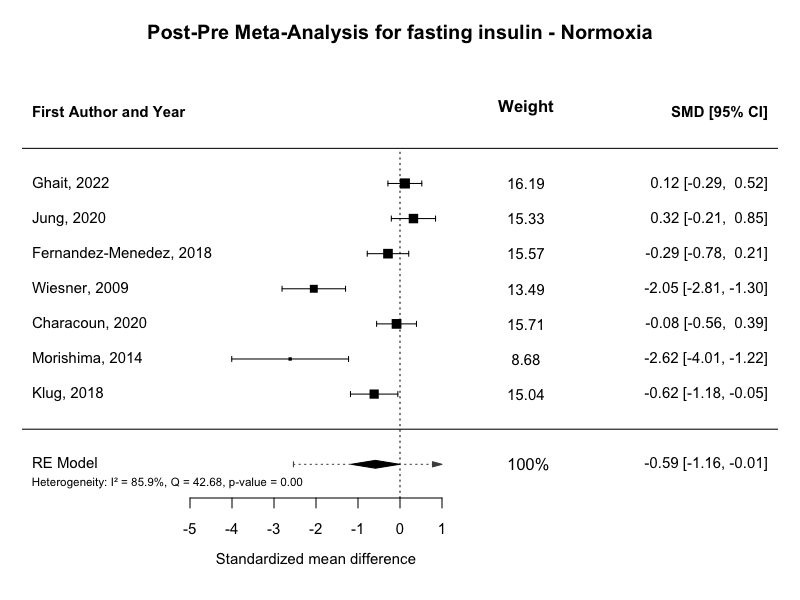

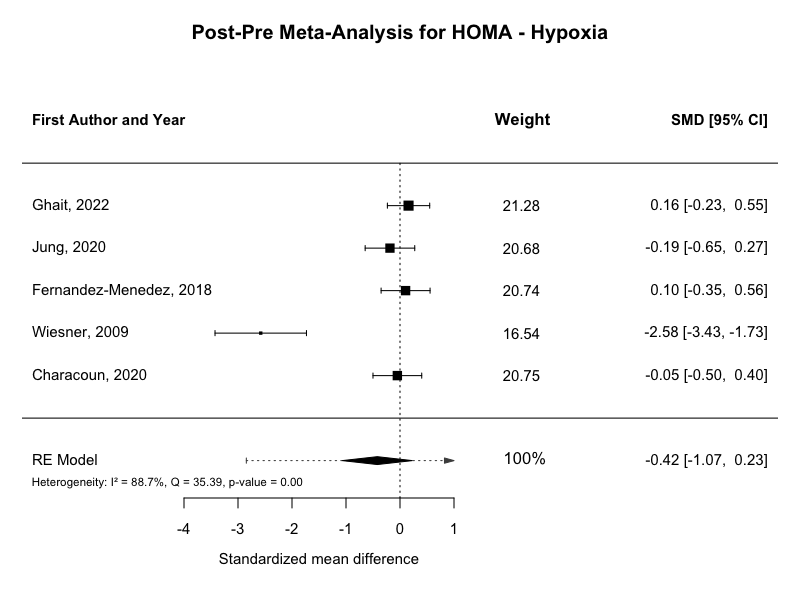

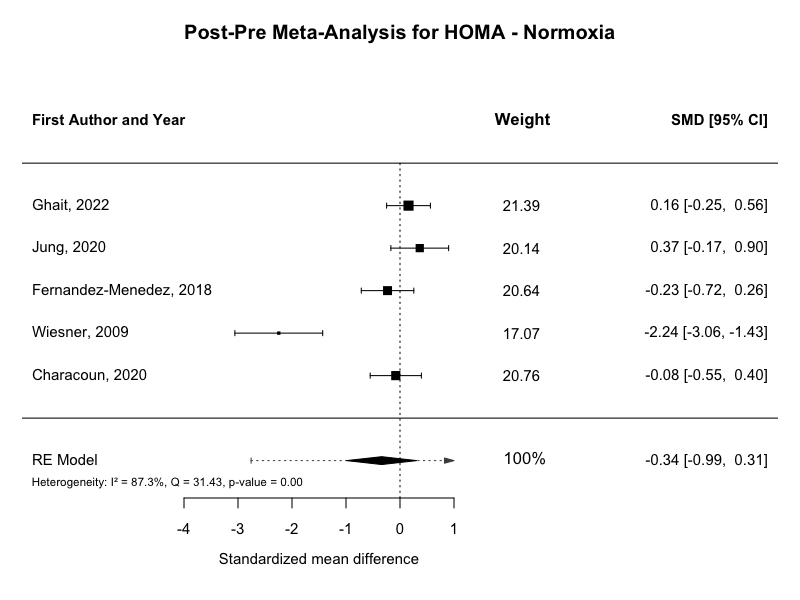

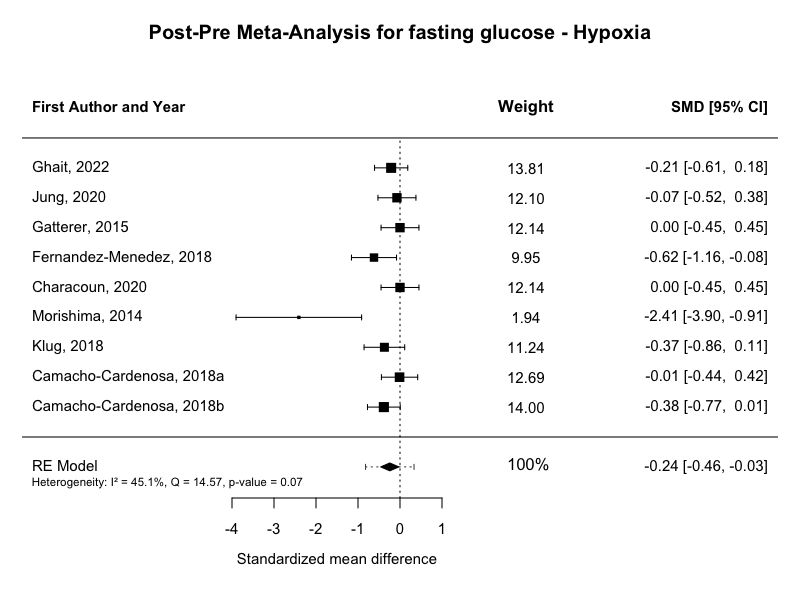


# Figure S2. Forest plot of standardized mean differences within groups (post-pre estimates) for fasting glucose, fasting insulin and homeostatic model assessment for insulin resistance (HOMA-IR). A negative value indicates a reduction in outcome after the intervention, whereas a positive value indicates an improvement in outcome after the intervention. Abbreviations: CI, confidence interval; SMD, standardized mean difference.


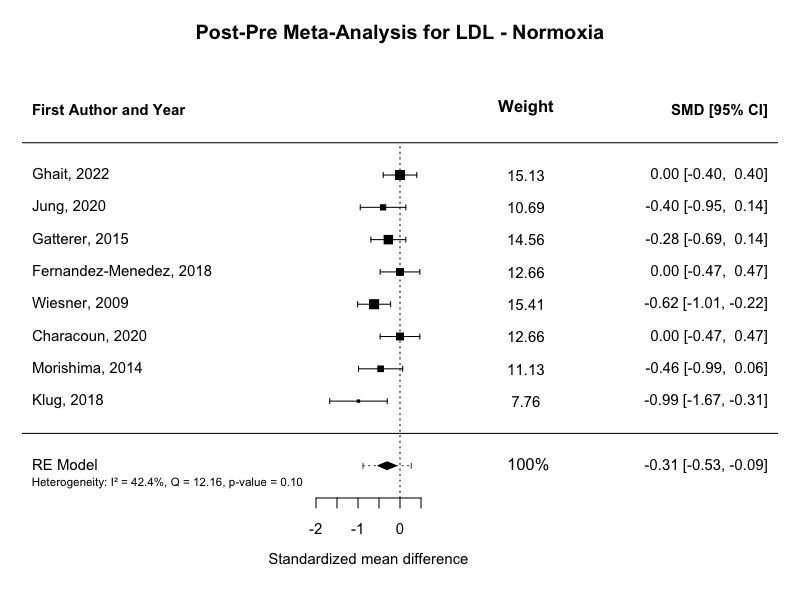

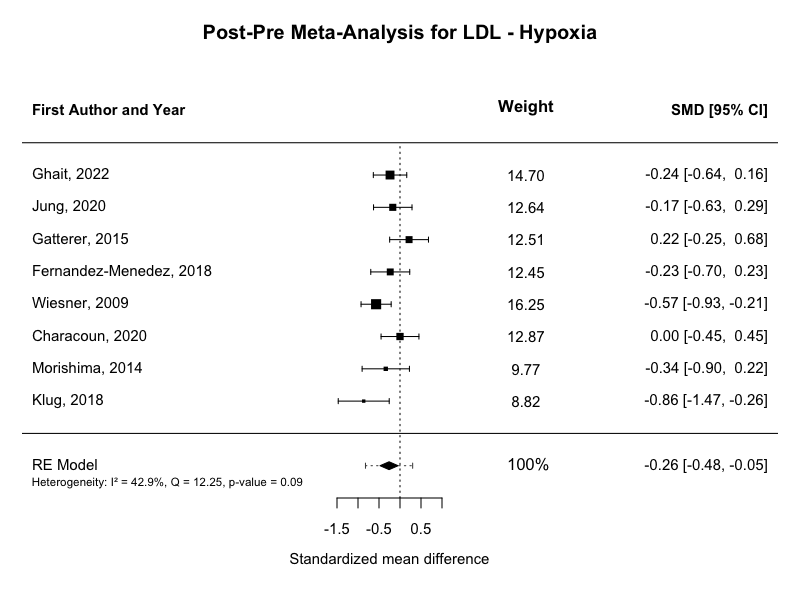

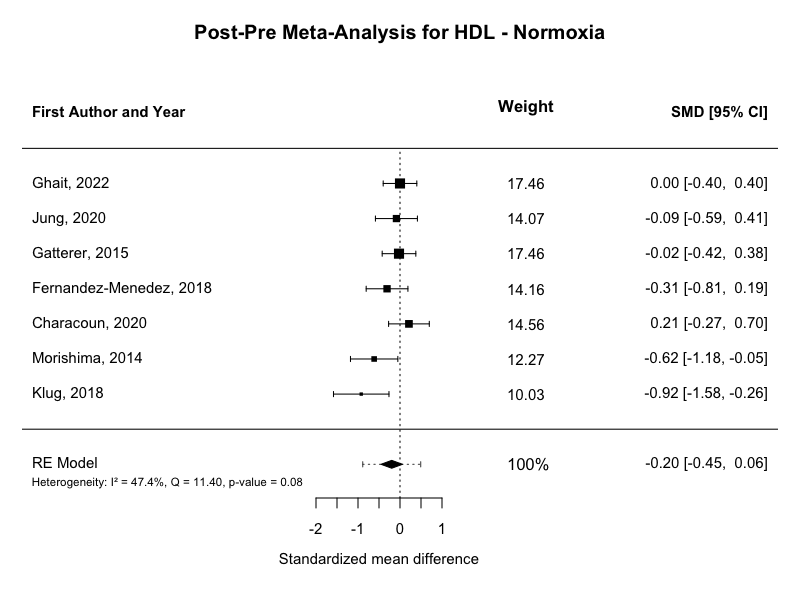

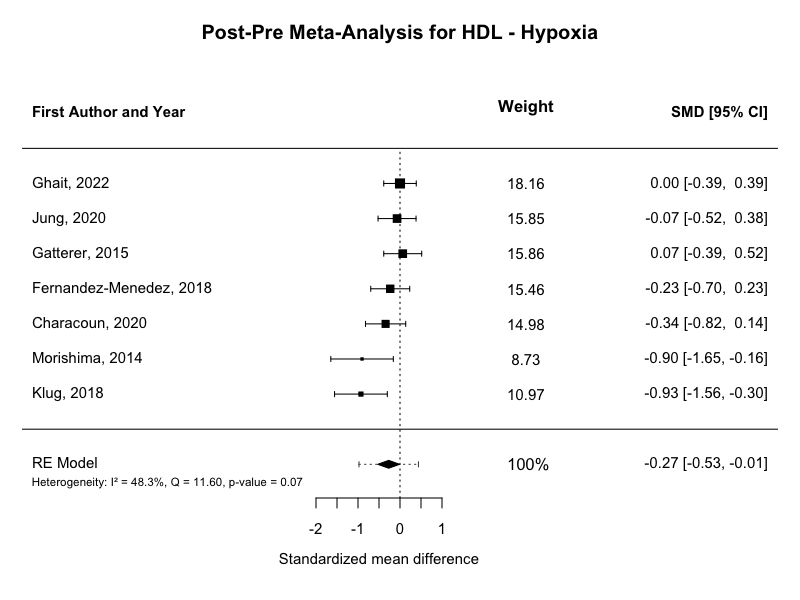


# Figure S3. Forest plot of standardized mean differences within groups (post-pre estimates) for low-density and high-density lipoprotein (LDL and HDL). A negative value indicates a reduction in outcome after the intervention, whereas a positive value indicates an improvement in outcome after the intervention. Abbreviations: CI, confidence interval; SMD, standardized mean difference.


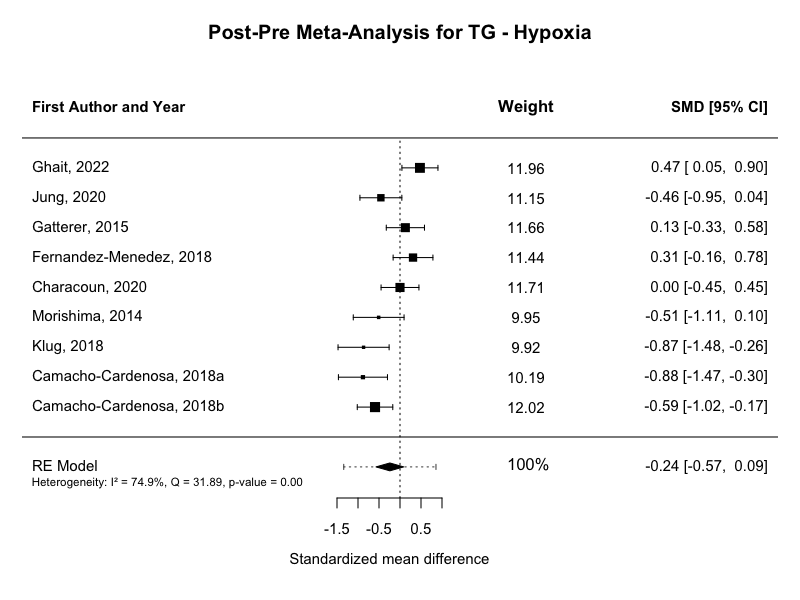

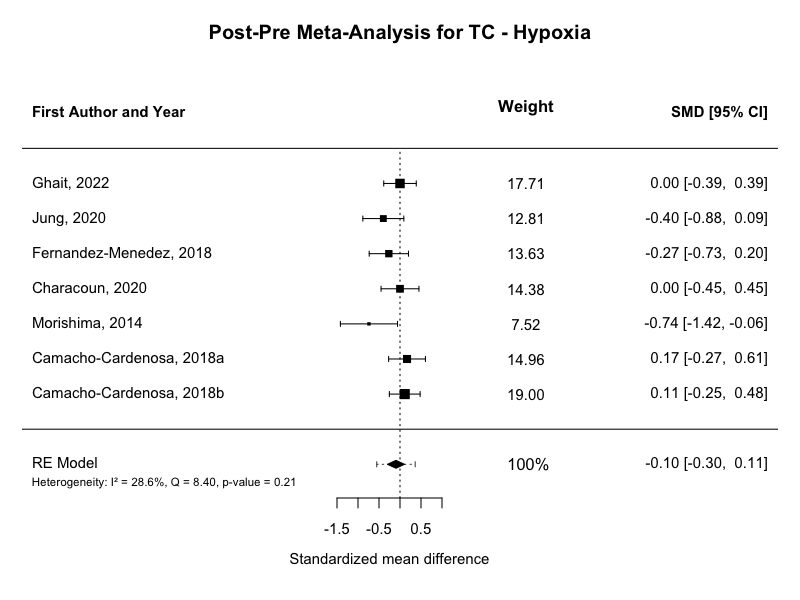

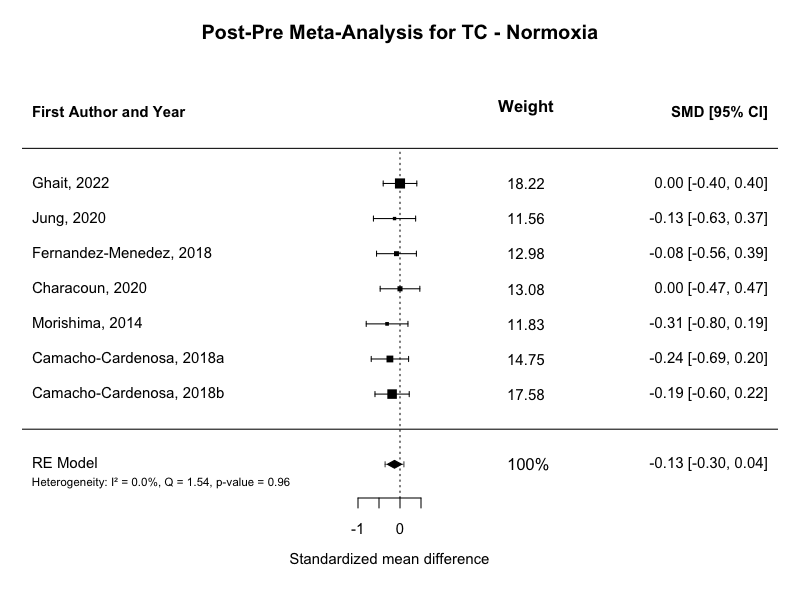

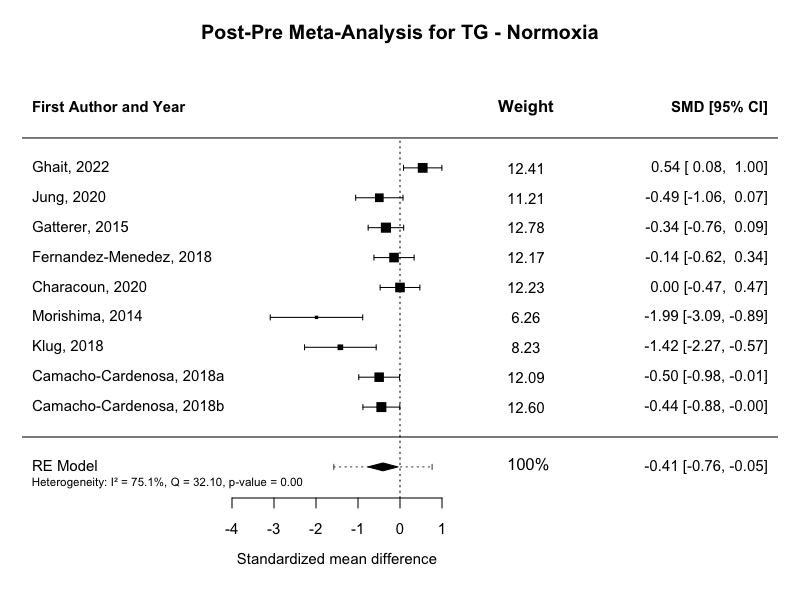


# Figure S4. Leave-one-out sensitivity analysis on the pooled allometric exponent of height and cardiorespiratory fitness (CRF) in boys.


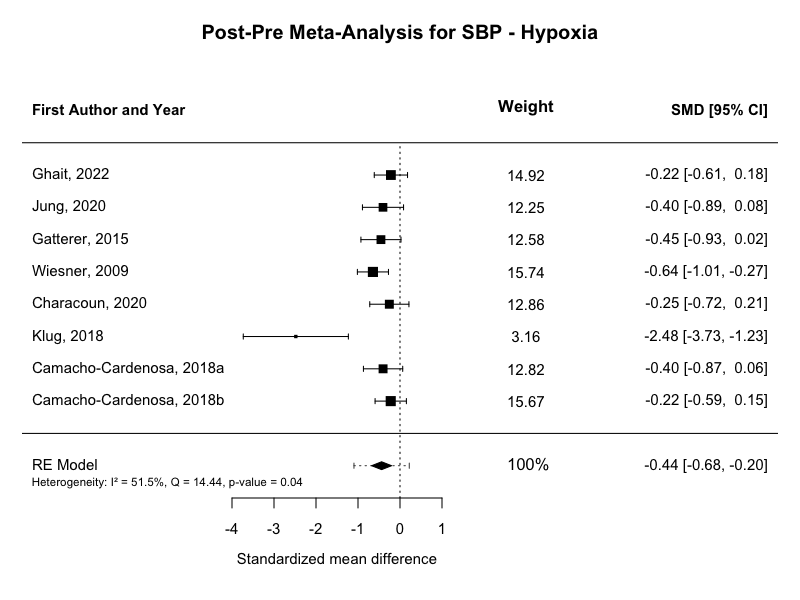

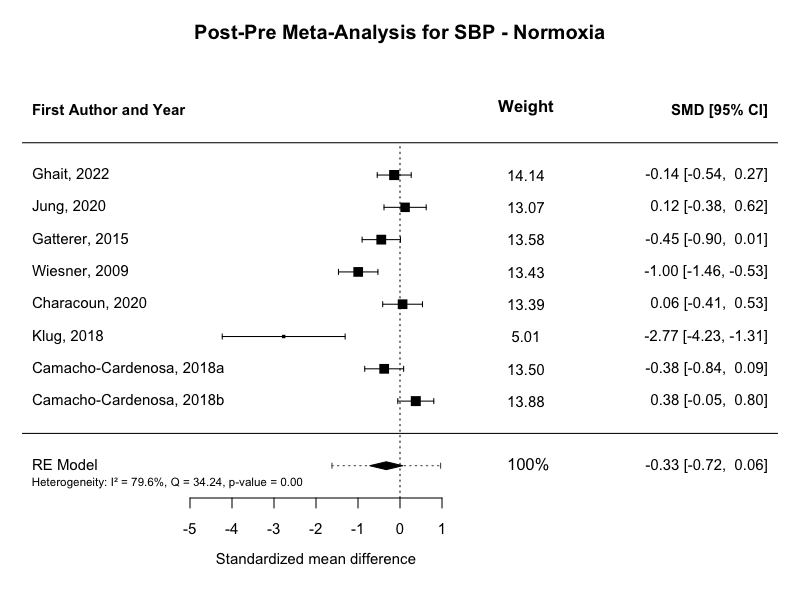

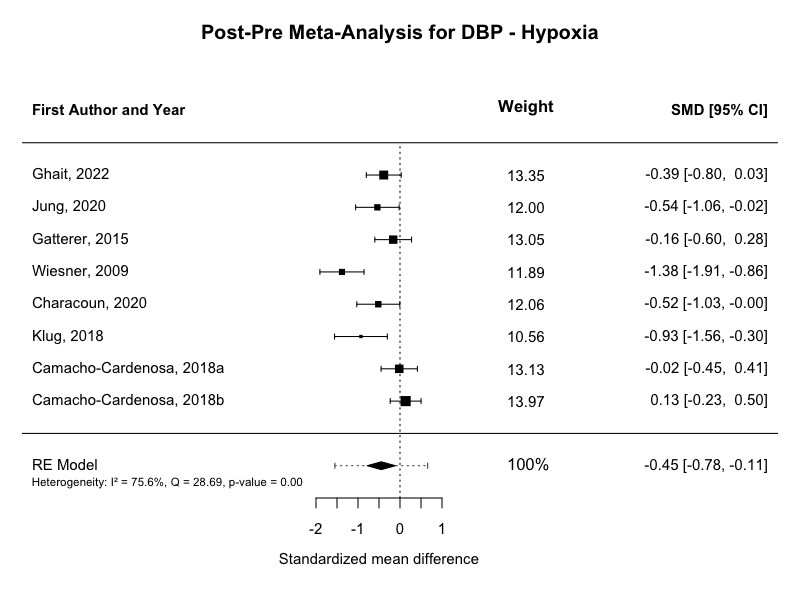

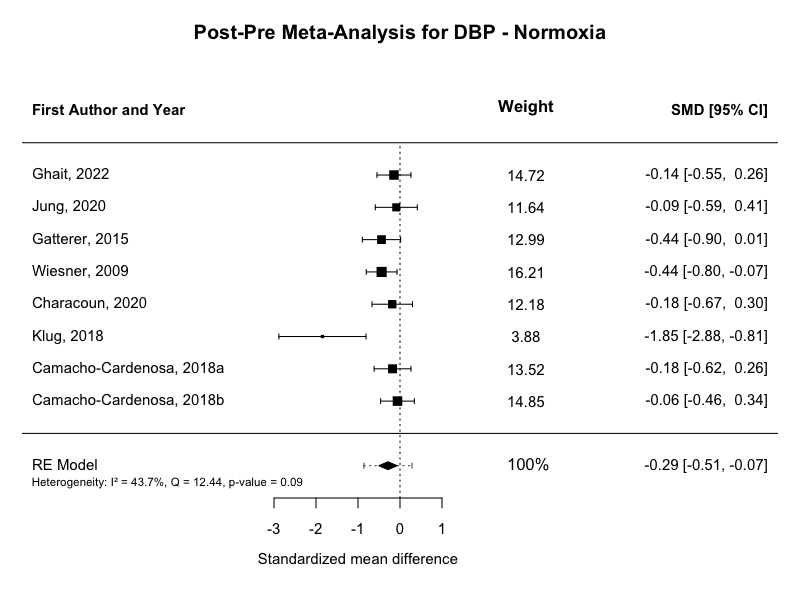


# Figure S5. Forest plot of standardized mean differences within groups (post-pre estimates) for systolic and diastolic blood pressure (SBP and DBP). A negative value indicates a reduction in outcome after the intervention, whereas a positive value indicates an improvement in outcome after the intervention. Abbreviations: CI, confidence interval; SMD, standardized mean difference.

**
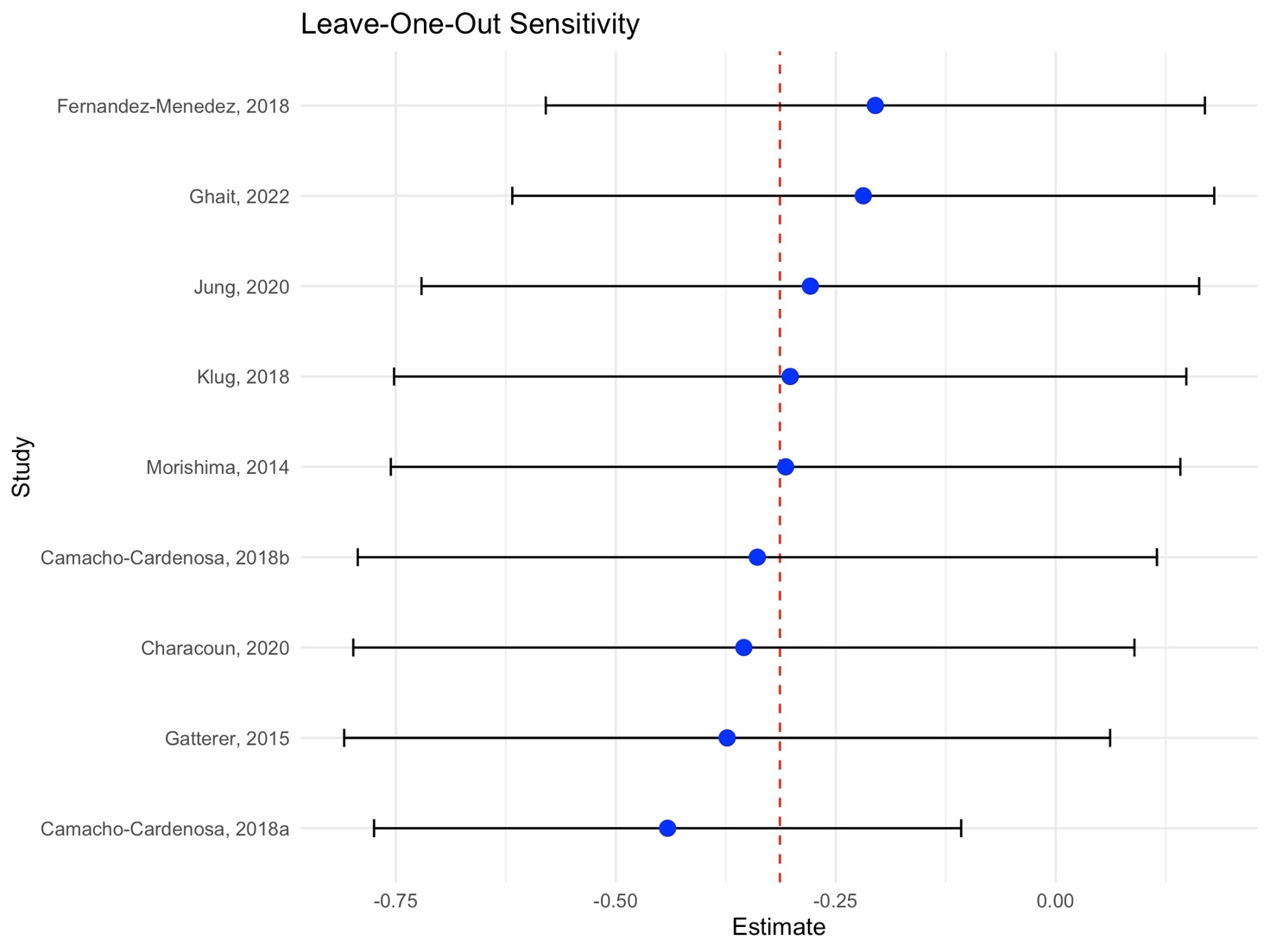
**

# Figure S6. Leave-one-out sensitivity analysis on the post-pre changes for fasting glucose.

**
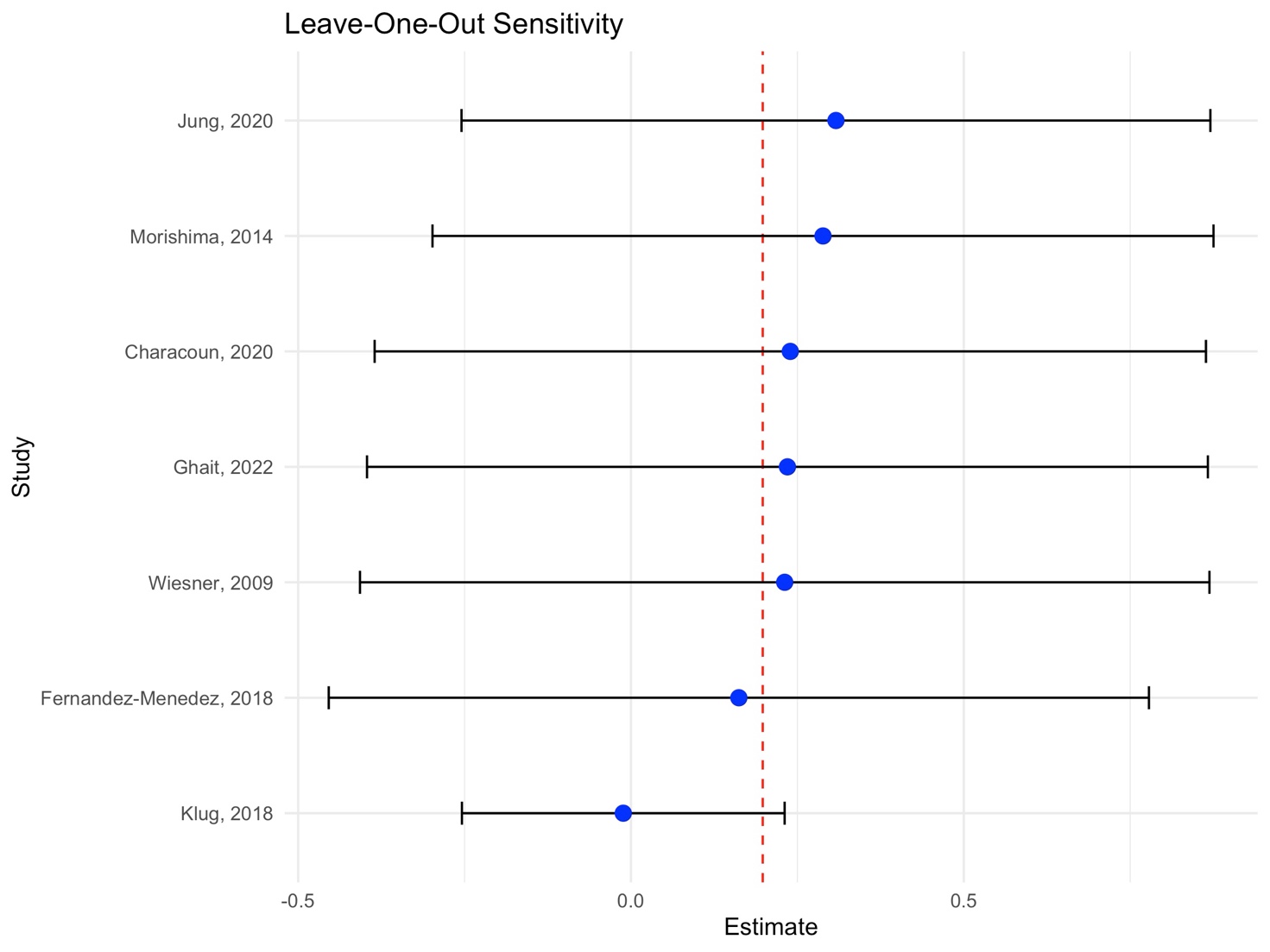
**

# Figure S7. Leave-one-out sensitivity analysis on the post-pre changes for fasting insulin.

**
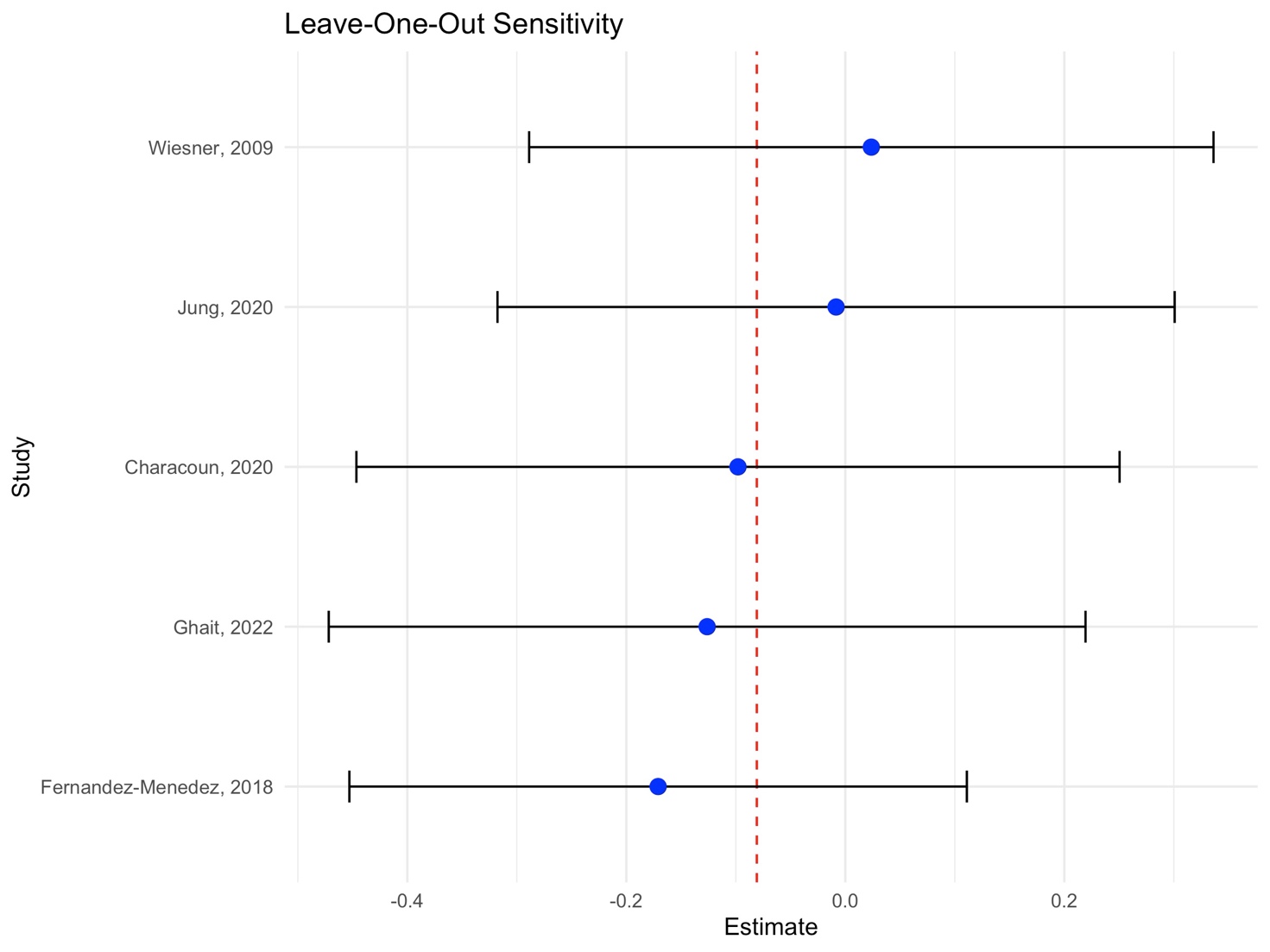
**

# Figure S8. Leave-one-out sensitivity analysis on the post-pre changes for homeostatic model assessment of insulin resistance (HOMA-IR).

**
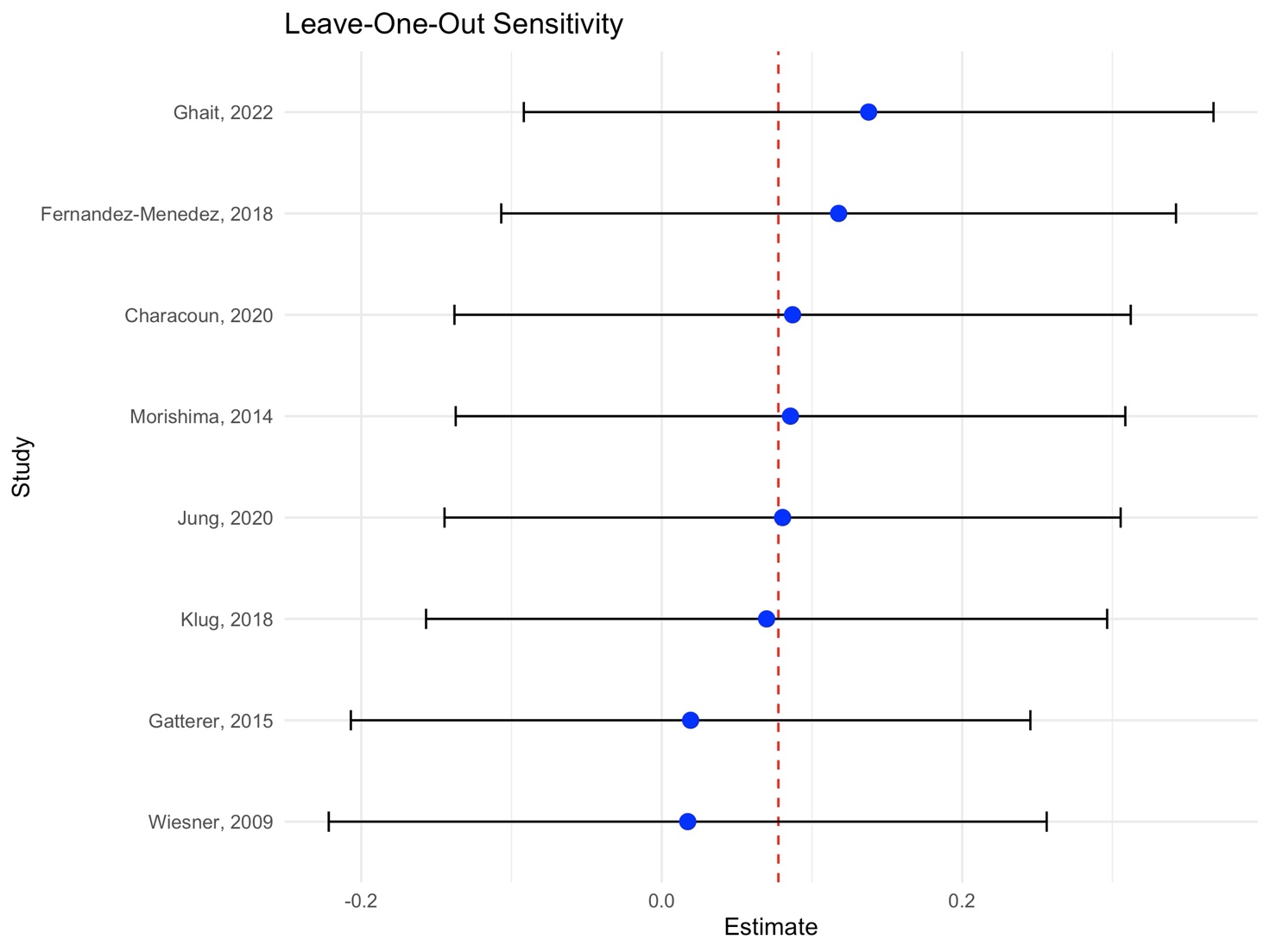
**

# Figure S9. Leave-one-out sensitivity analysis on the post-pre changes for low-density lipoprotein (LDL-C).

**
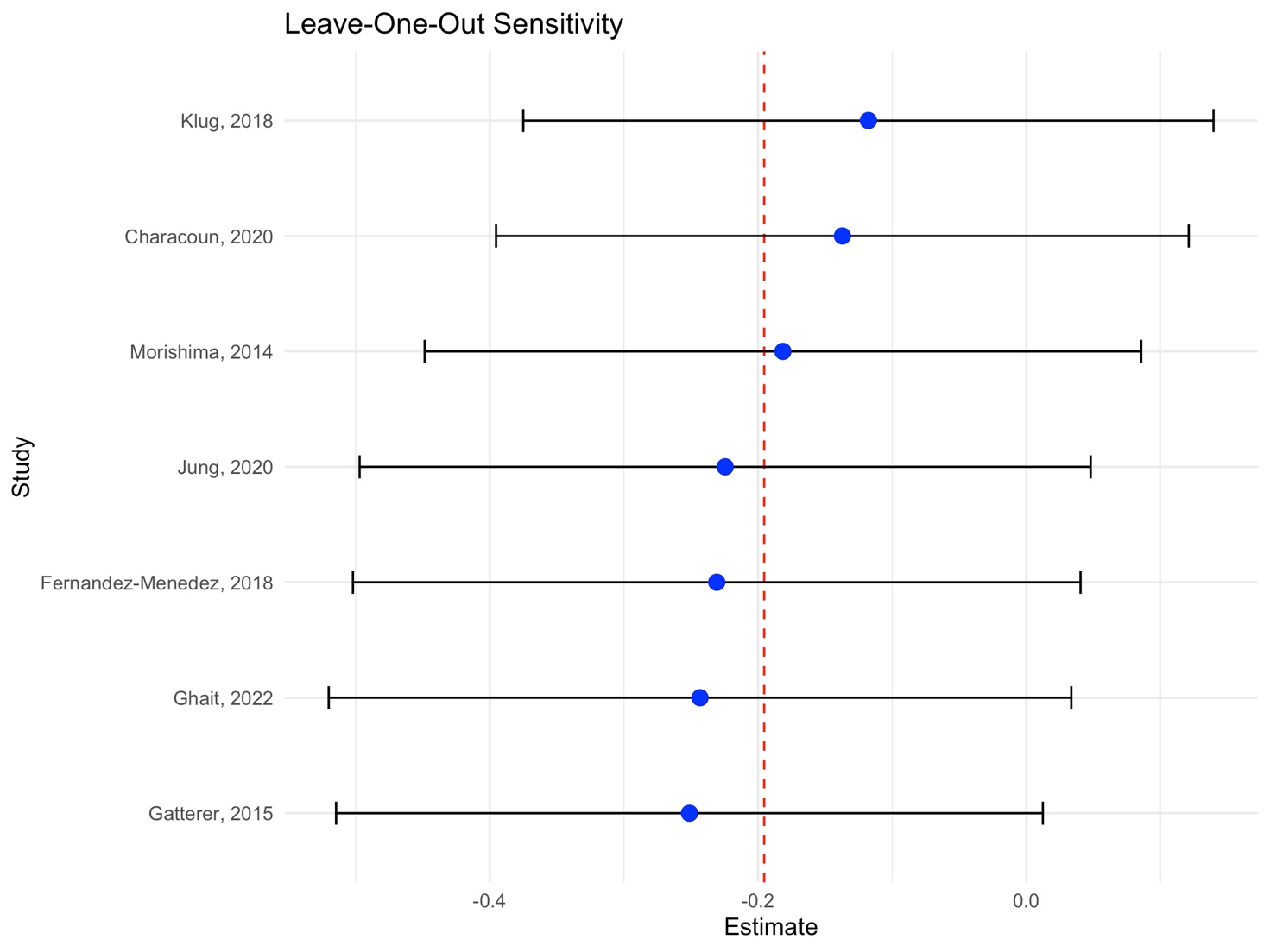
**

# Figure S10. Leave-one-out sensitivity analysis on the post-pre changes for high-density lipoprotein (HDL-C).

**
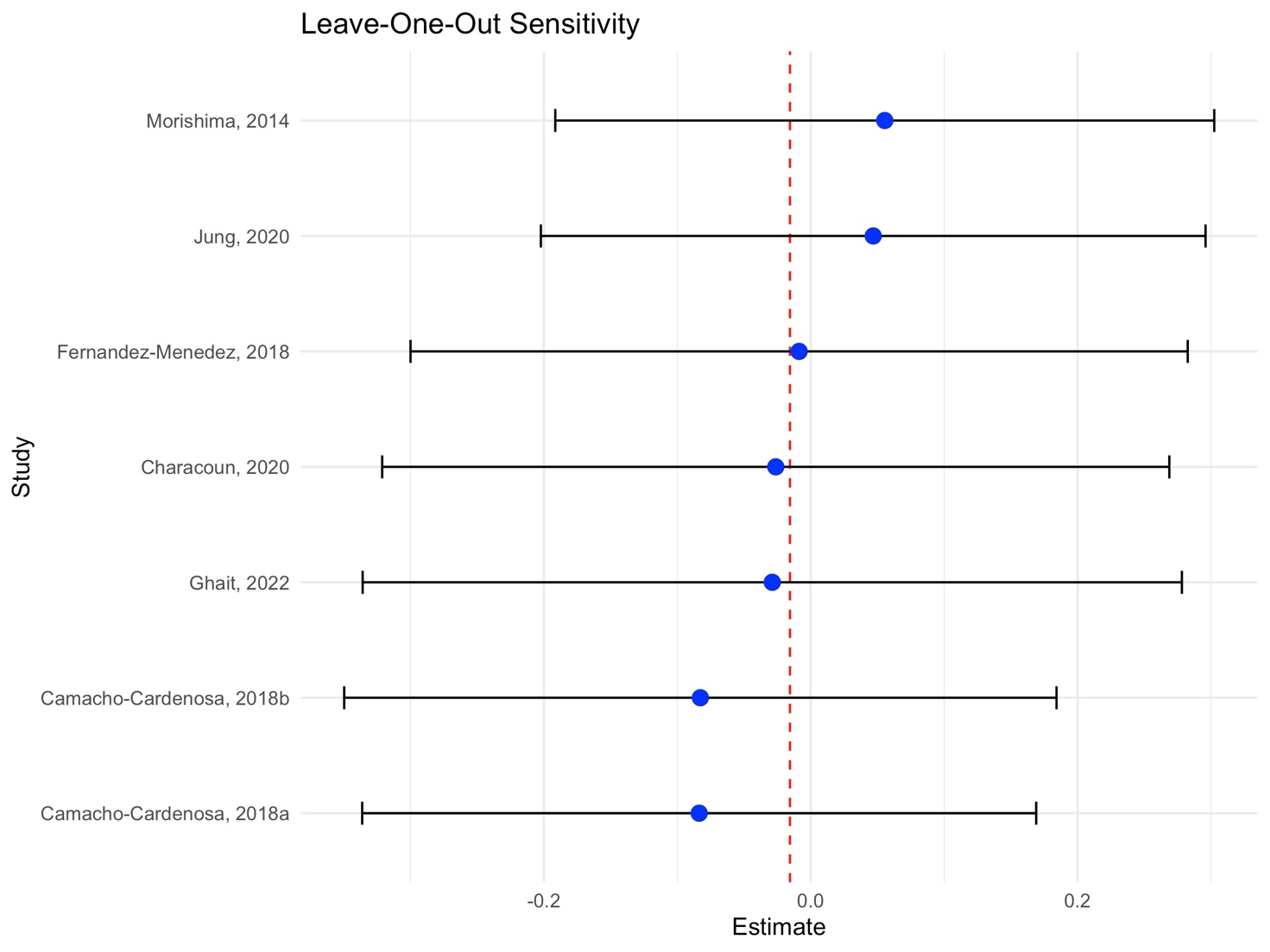
**

# Figure S11. Leave-one-out sensitivity analysis on the post-pre changes for total cholesterol.

**
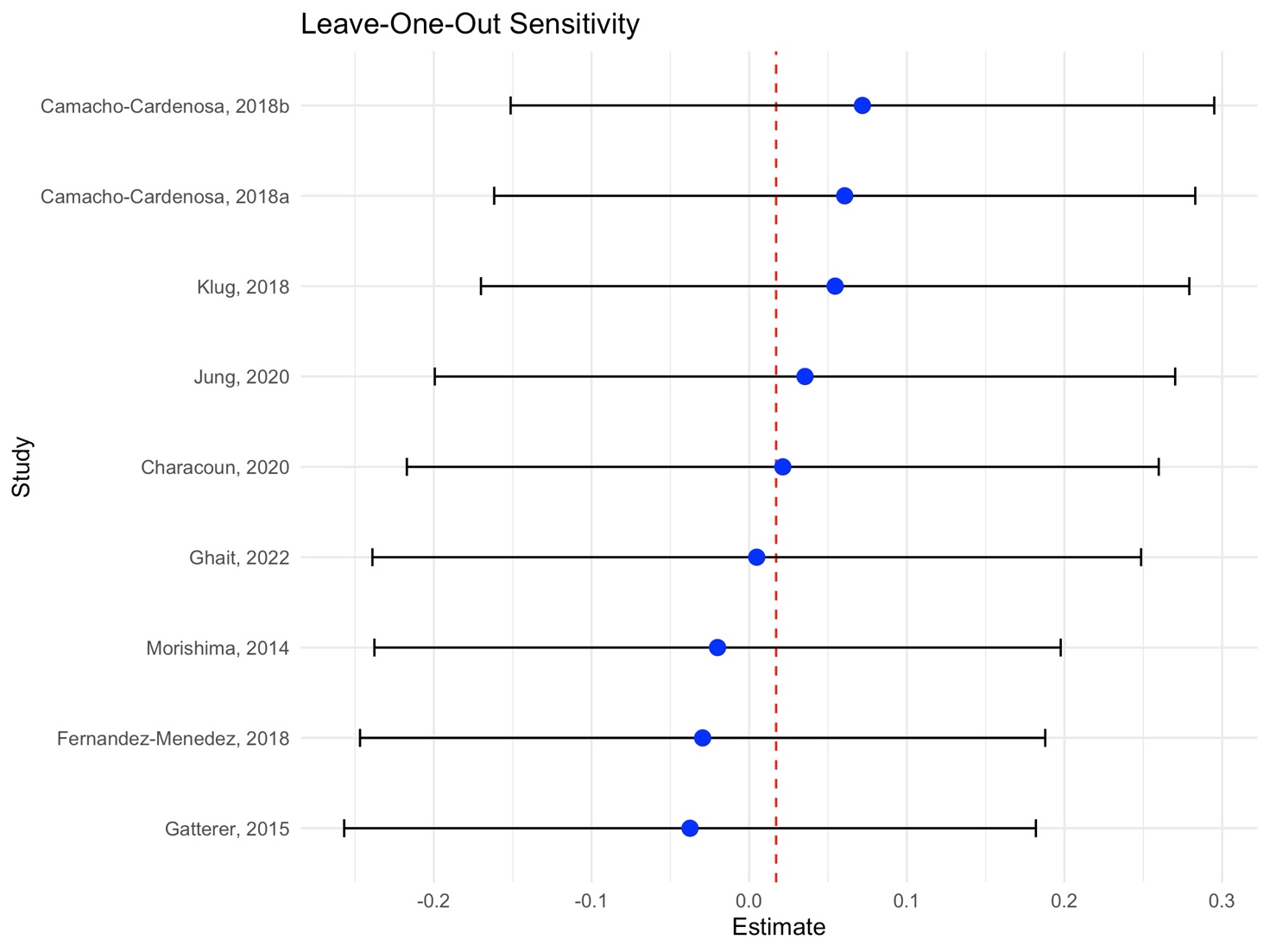
**

# Figure S12. Leave-one-out sensitivity analysis on the post-pre changes for triglycerides.

**
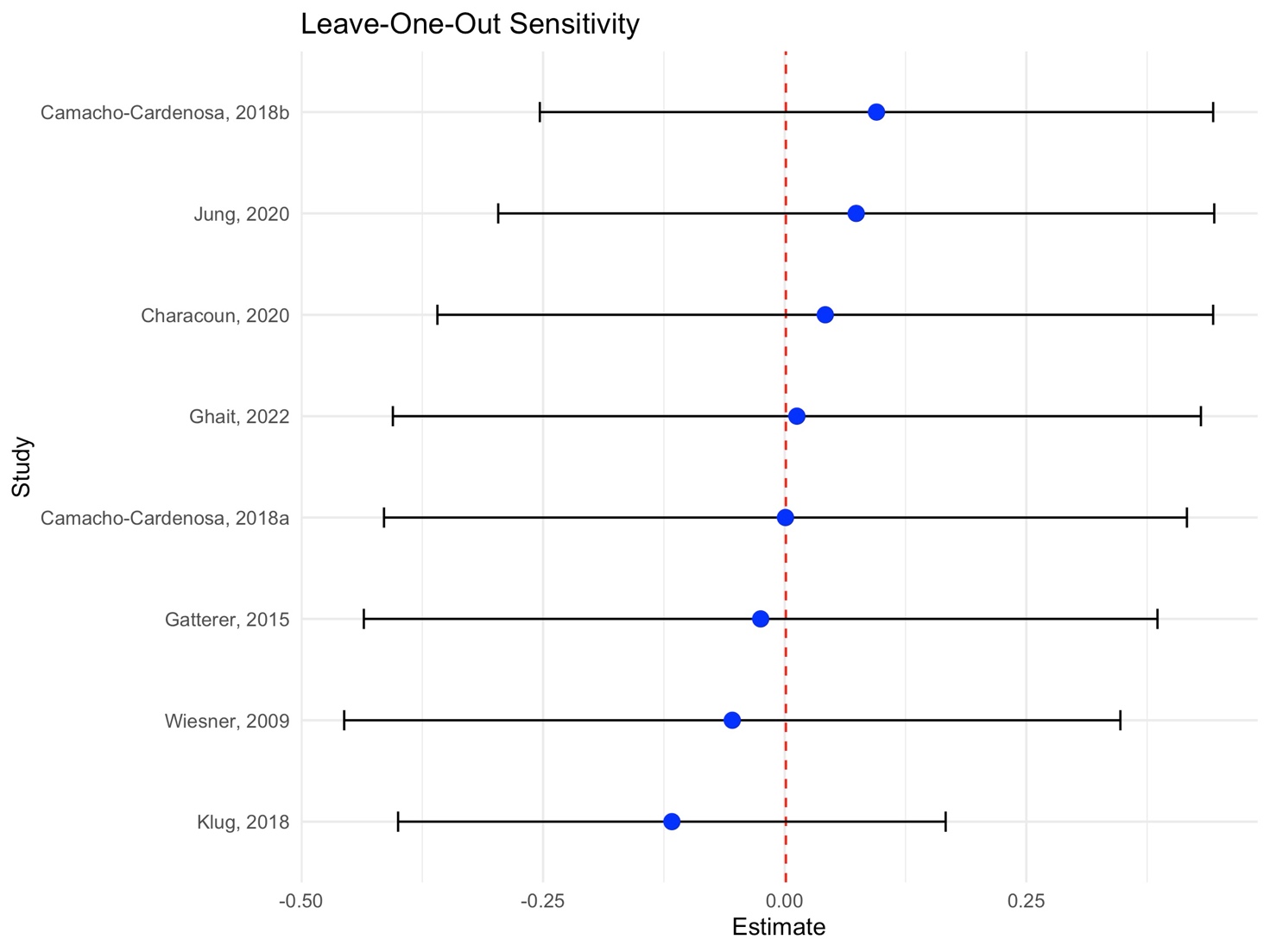
**

# Figure S13. Leave-one-out sensitivity analysis on the post-pre changes for systolic blood pressure (SBP).

**
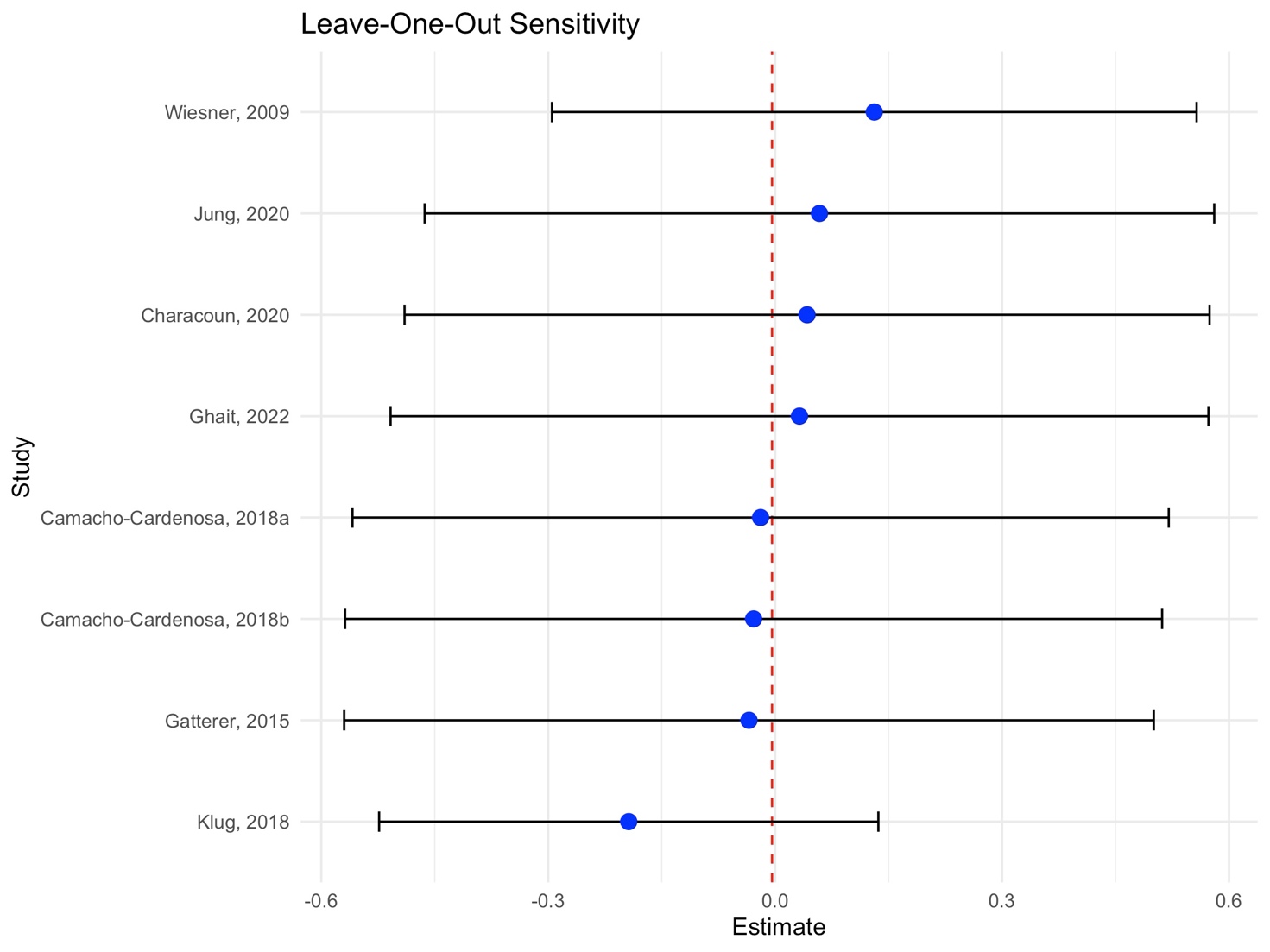
**

# Figure S14. Leave-one-out sensitivity analysis on the post-pre changes for diastolic blood pressure (DBP).

**
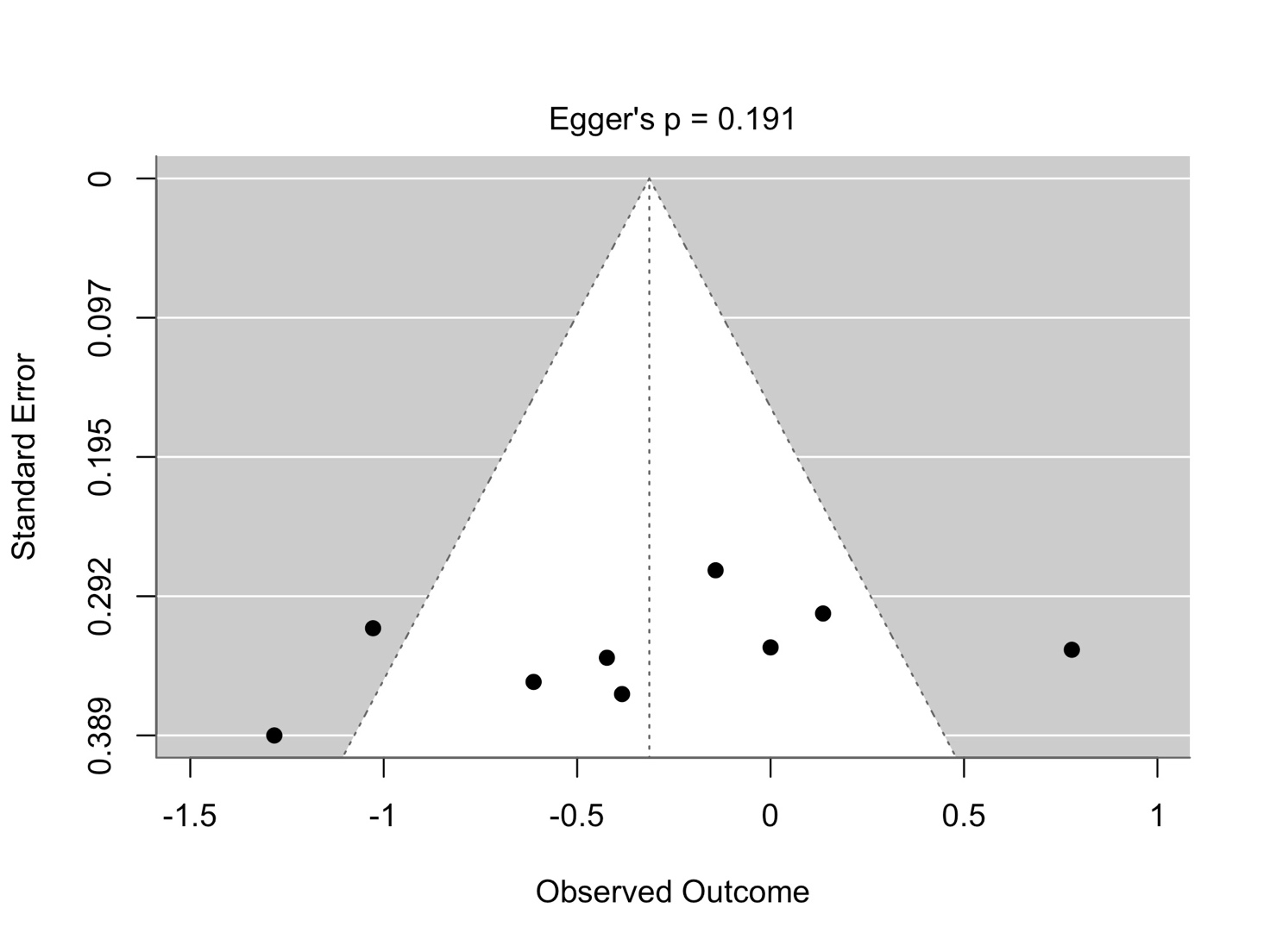
**

# Figure S15. Funnel plot of the meta-analysis showing observed change versus standard error across studies of fasting glucose for post-pre changes between the two groups.

**
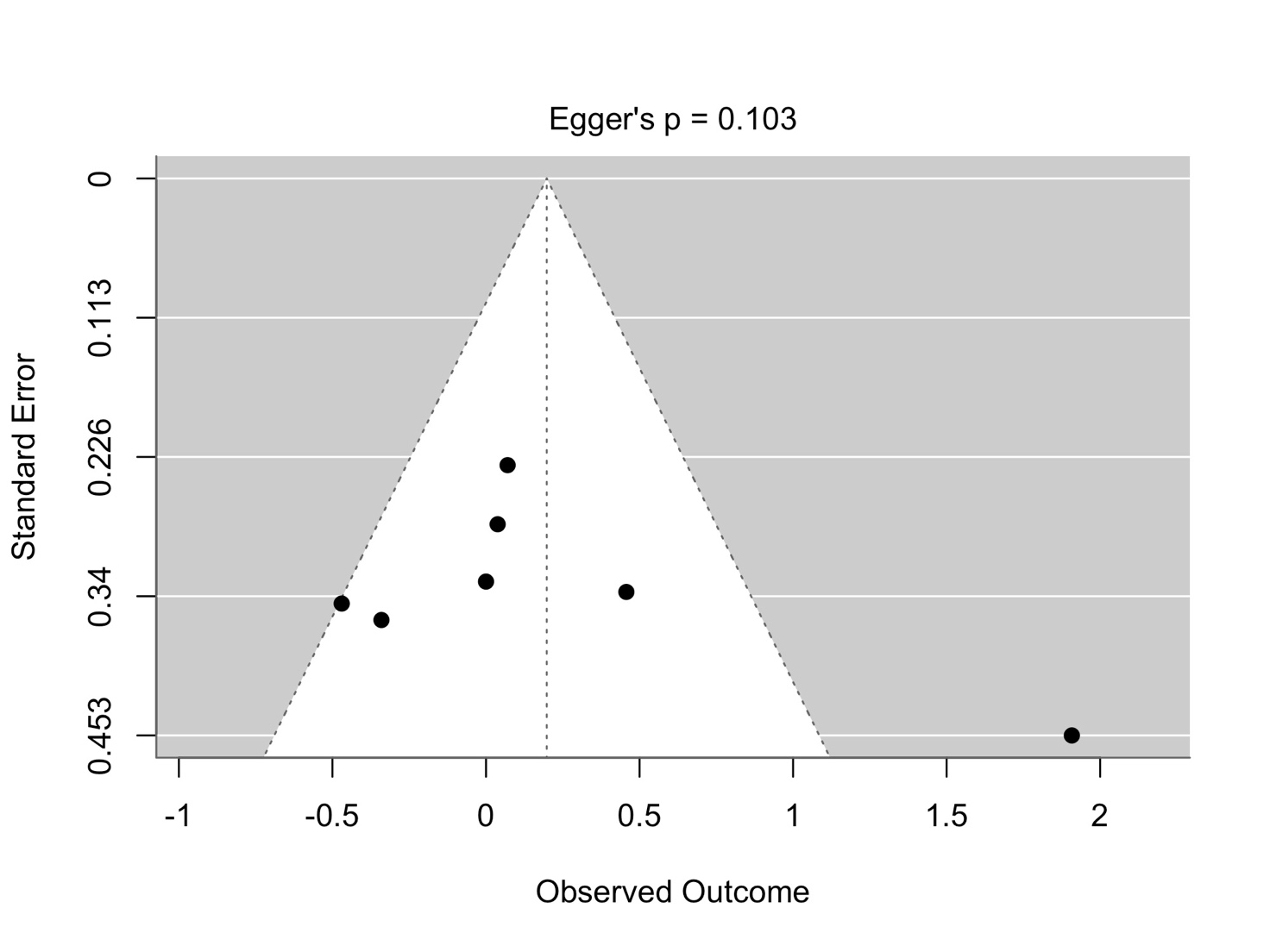
**

# Figure S16. Funnel plot of the meta-analysis showing observed change versus standard error across studies of fasting insulin for post-pre changes between the two groups.

**
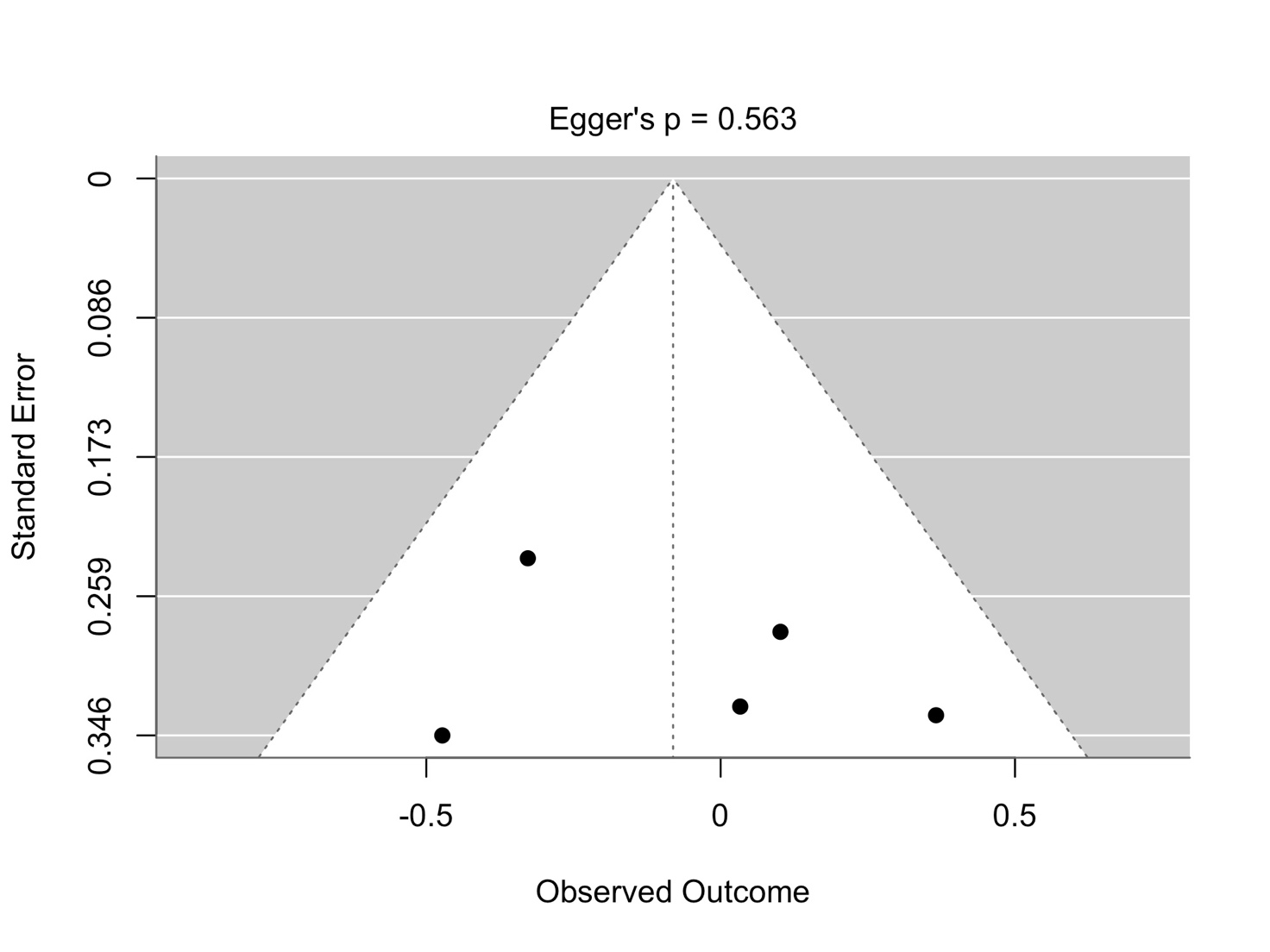
**

# Figure S17. Funnel plot of the meta-analysis showing observed change versus standard error across studies of homeostatic model assessment of insulin resistance (HOMA-IR) for post-pre changes between the two groups.

**
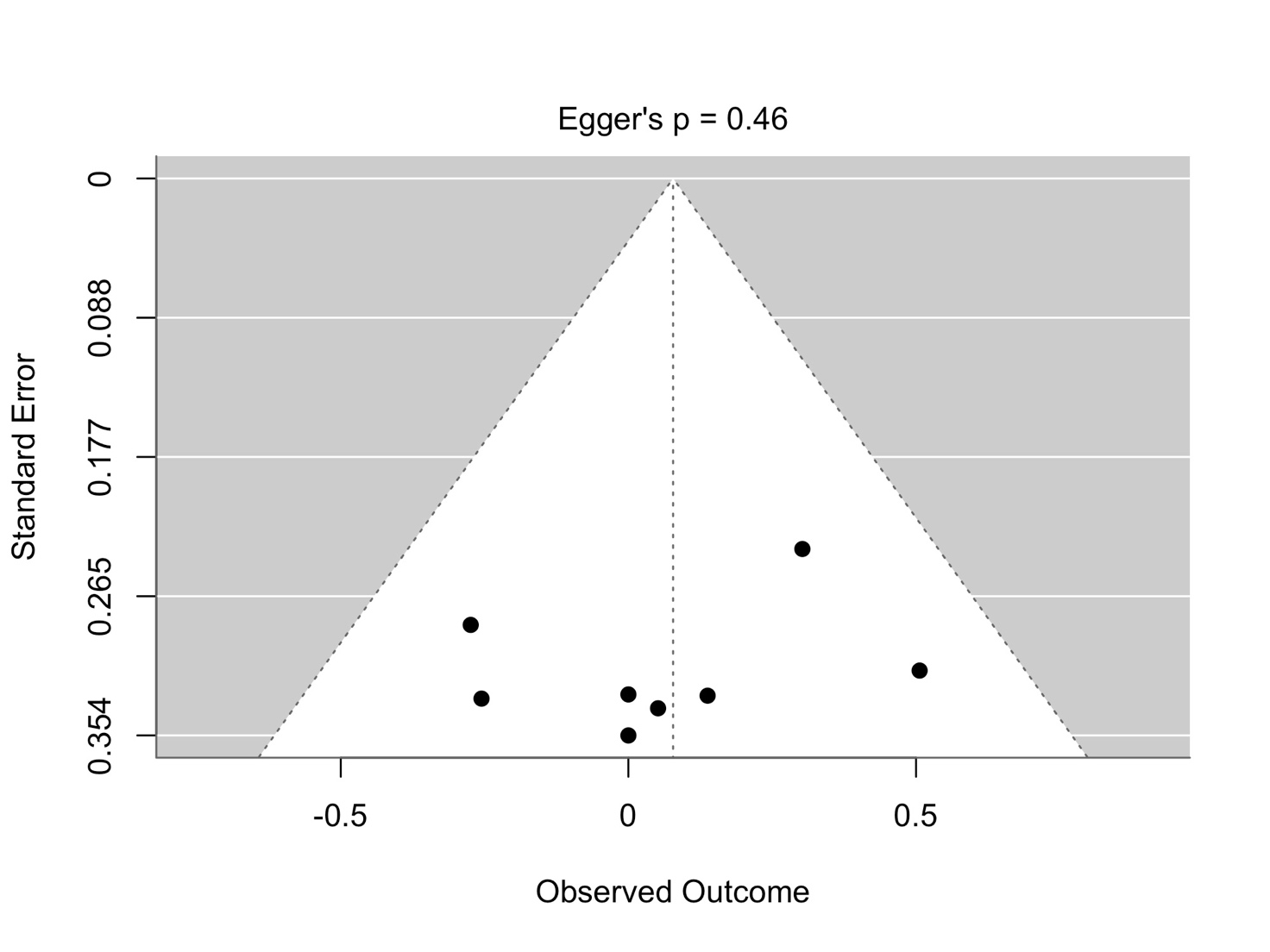
**

# Figure S18. Funnel plot of the meta-analysis showing observed change versus standard error across studies of low-density lipoprotein (LDL-C) for post-pre changes between the two groups.

**
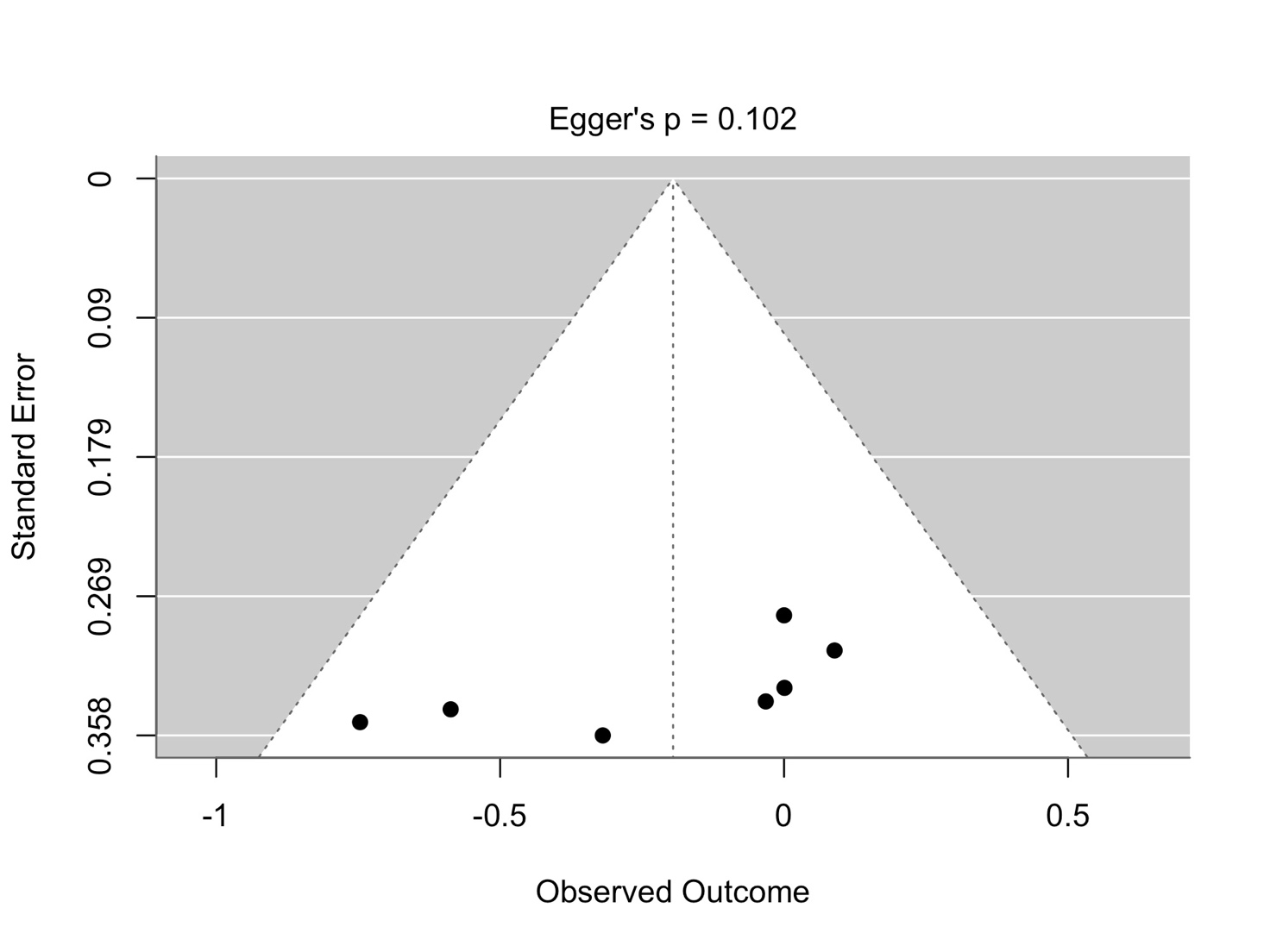
**

# Figure S19. Funnel plot of the meta-analysis showing observed change versus standard error across studies of high-density lipoprotein (HDL-C) for post-pre changes between the two groups.

**
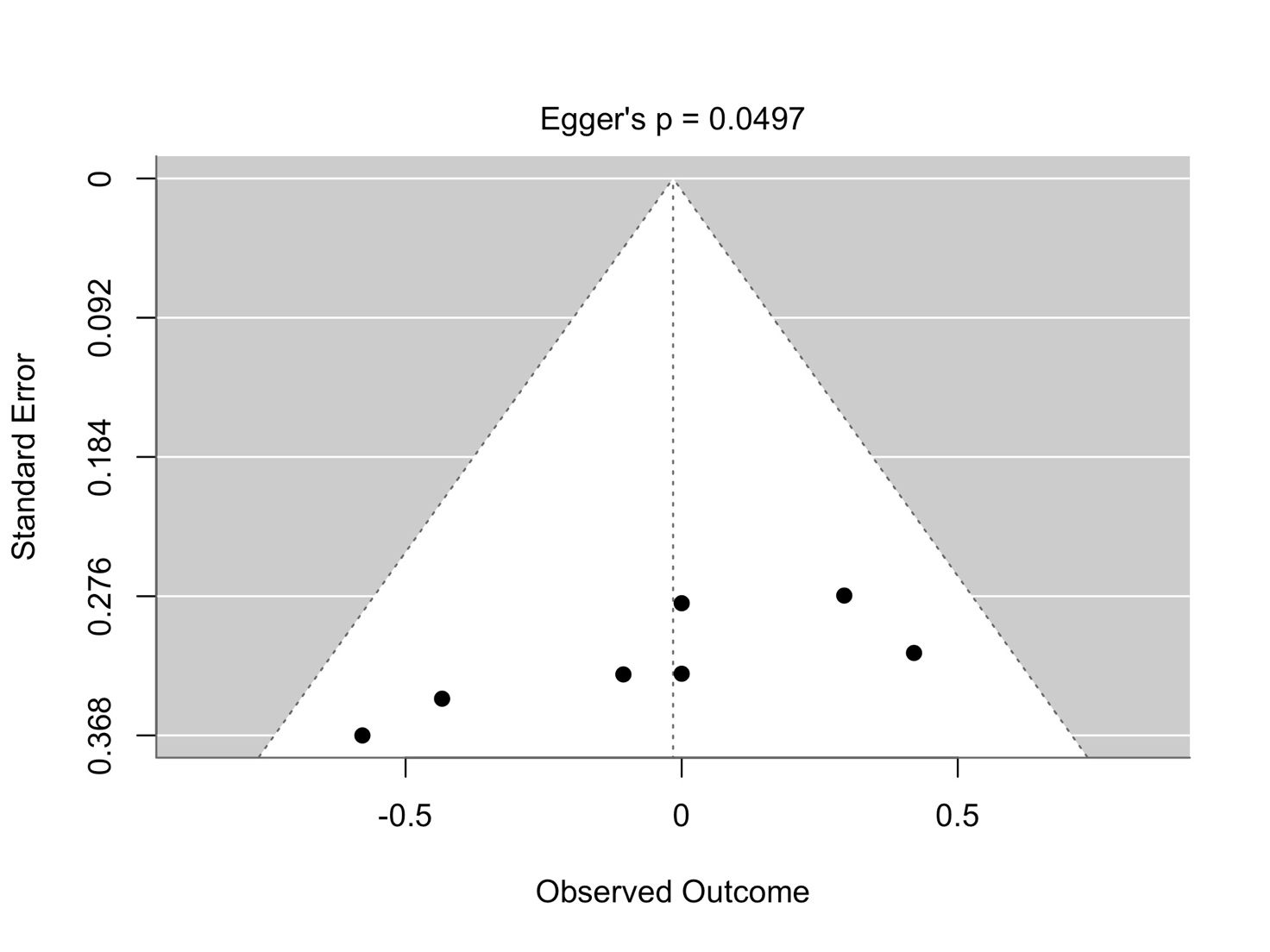
**

# Figure S20. Funnel plot of the meta-analysis showing observed change versus standard error across studies of total cholesterol for post-pre changes between the two groups.

**
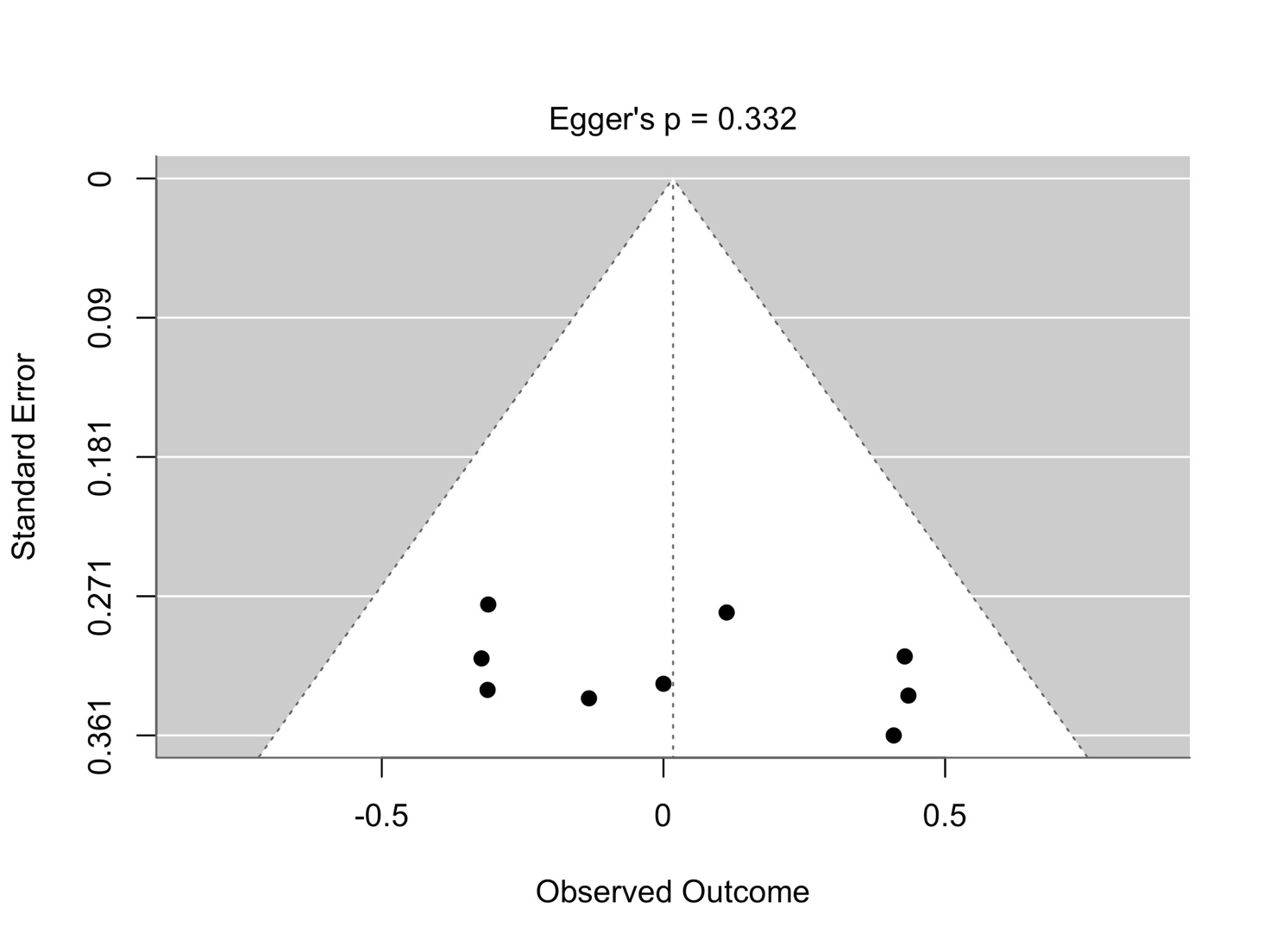
**

# Figure S21. Funnel plot of the meta-analysis showing observed change versus standard error across studies of triglycerides for post-pre changes between the two groups.

**
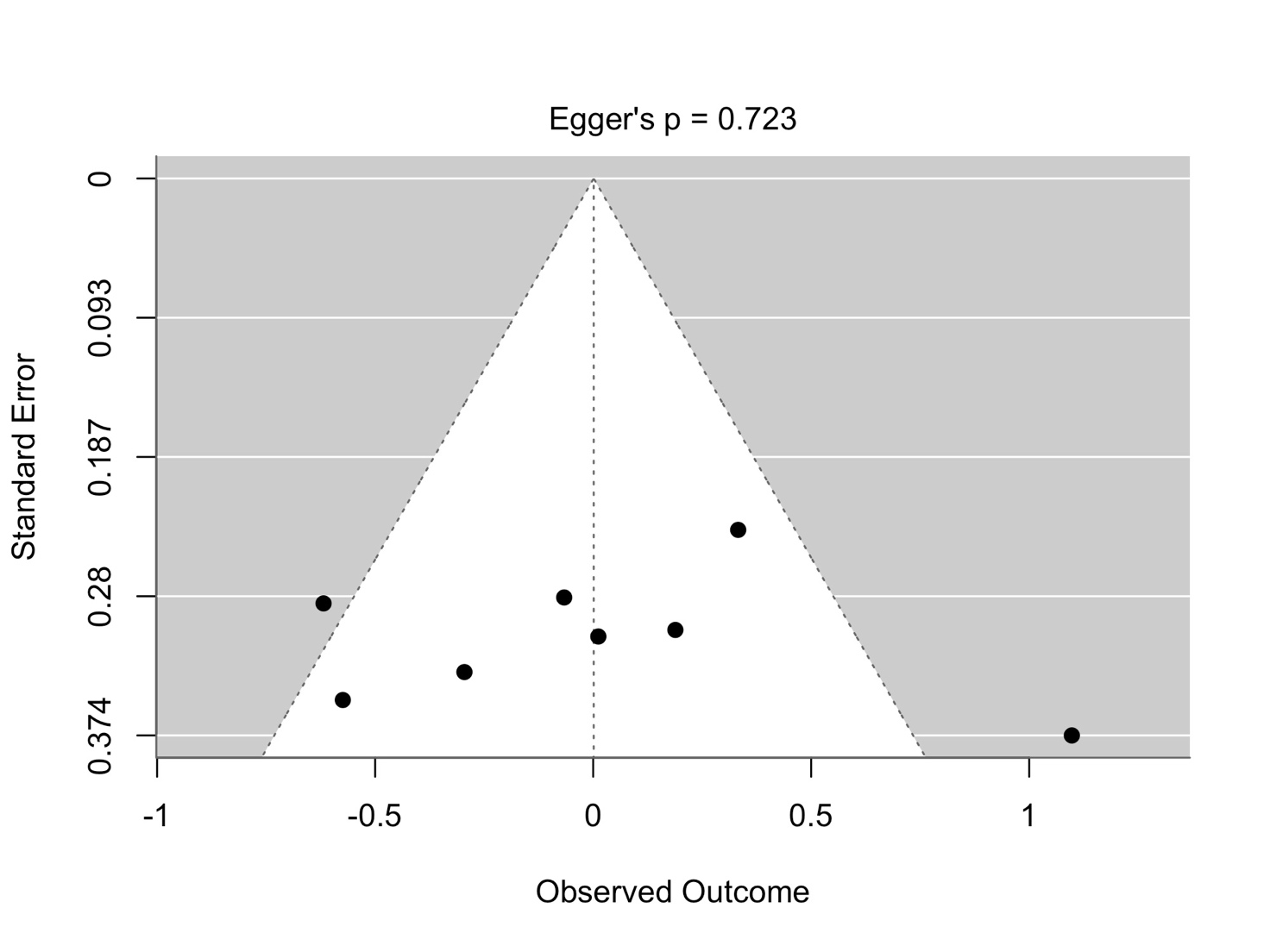
**

# Figure S22. Funnel plot of the meta-analysis showing observed change versus standard error across studies of systolic blood pressure for post-pre changes between the two groups.

**
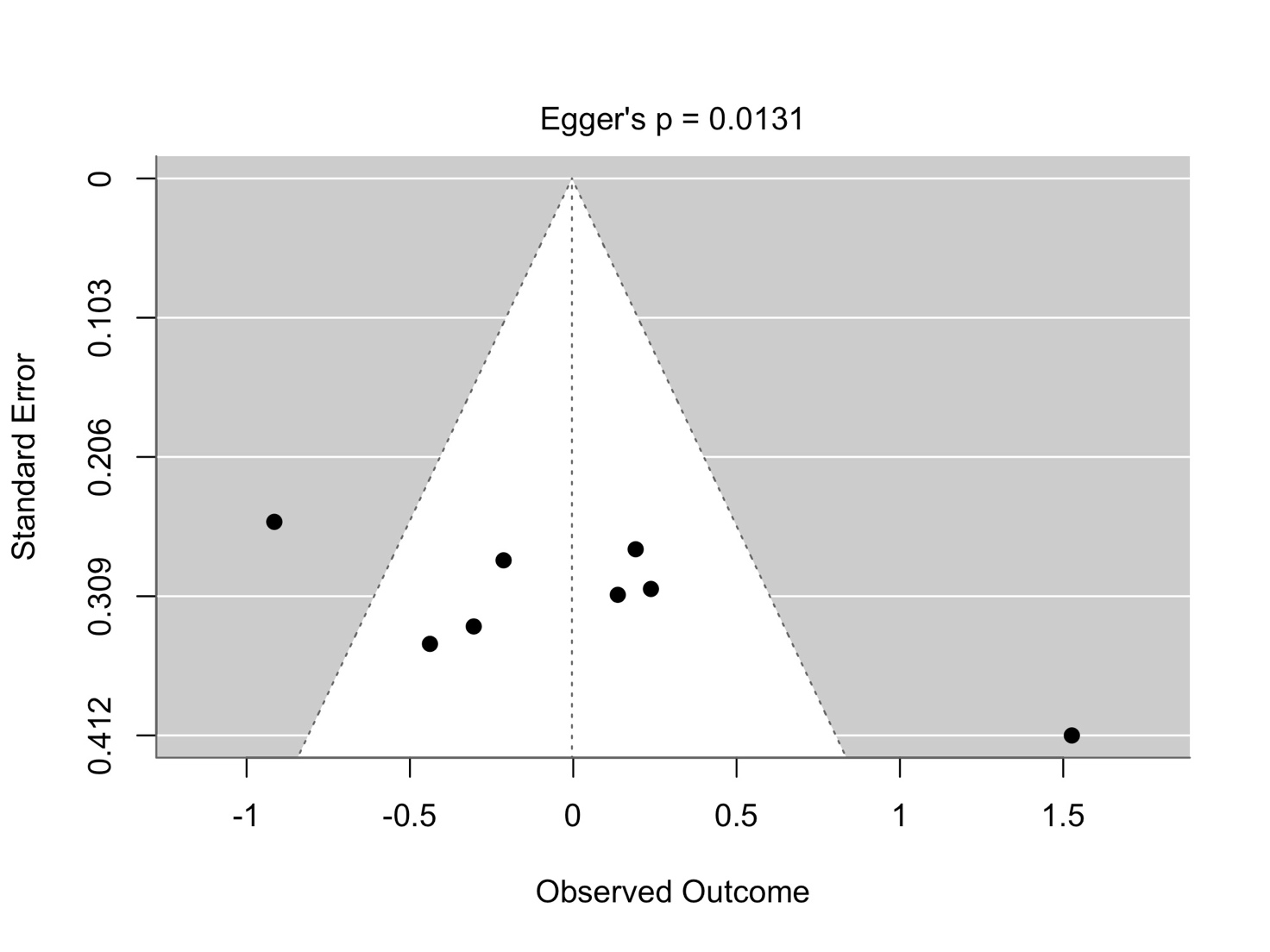
**

# Figure S23. Funnel plot of the meta-analysis showing observed change versus standard error across studies of diastolic blood pressure for post-pre changes between the two groups.

**
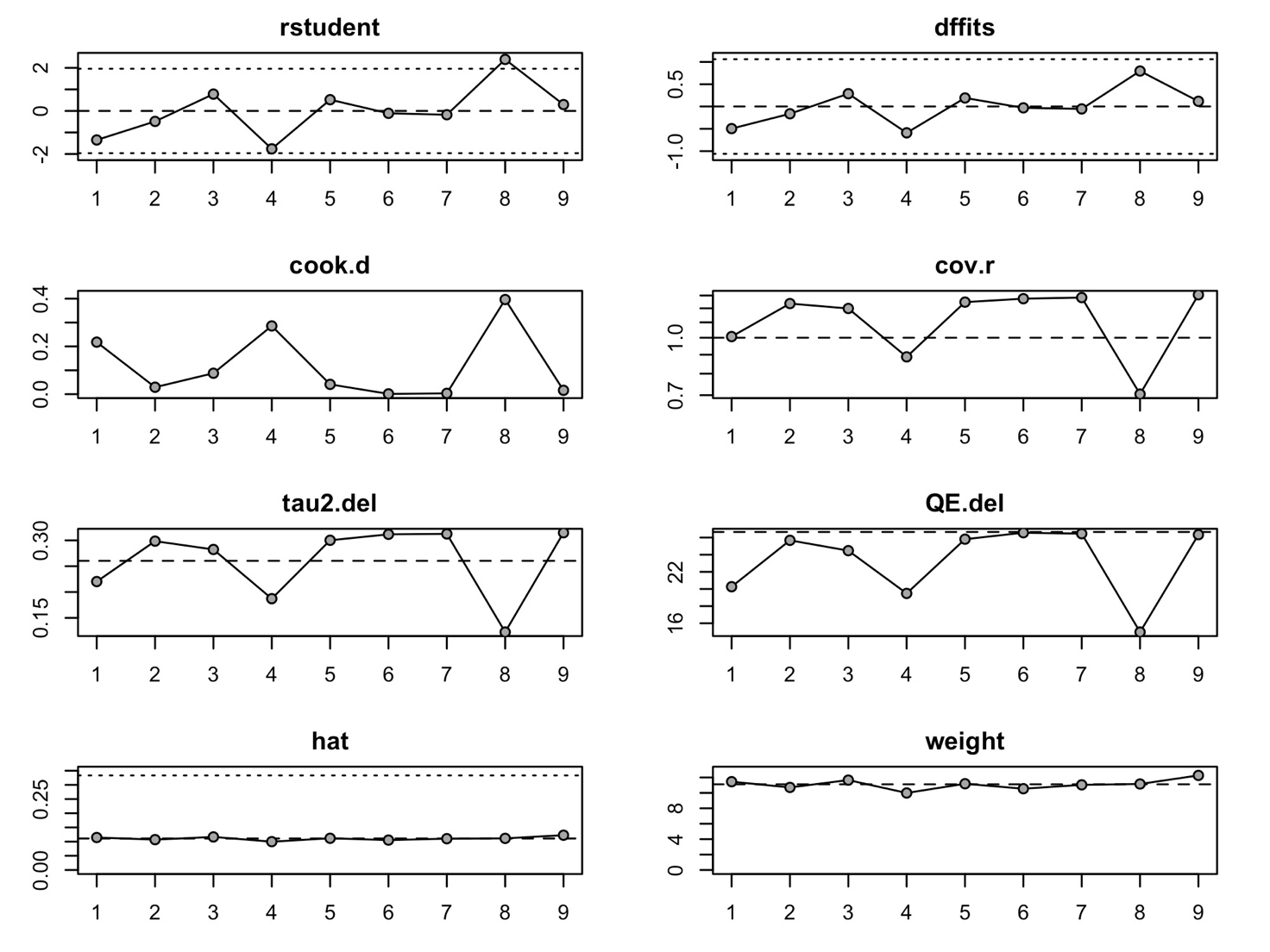
**

# Figure S24. Influence diagnostics for the meta-analysis of post-pre changes between groups for fasting glucose.

This figure summarizes influence statistics across eight diagnostic plots, each assessing how individual studies affect the overall model estimates and heterogeneity. Abbreviations: rstudent = standardized residuals; cook.d = Cook’s distance; dffits = difference in fits; cov.r = covariance ratio; tau2.del = change in between-study variance (τ²); QE.del = change in heterogeneity statistic (Q); hat = leverage; weight = study weight in the model.

**
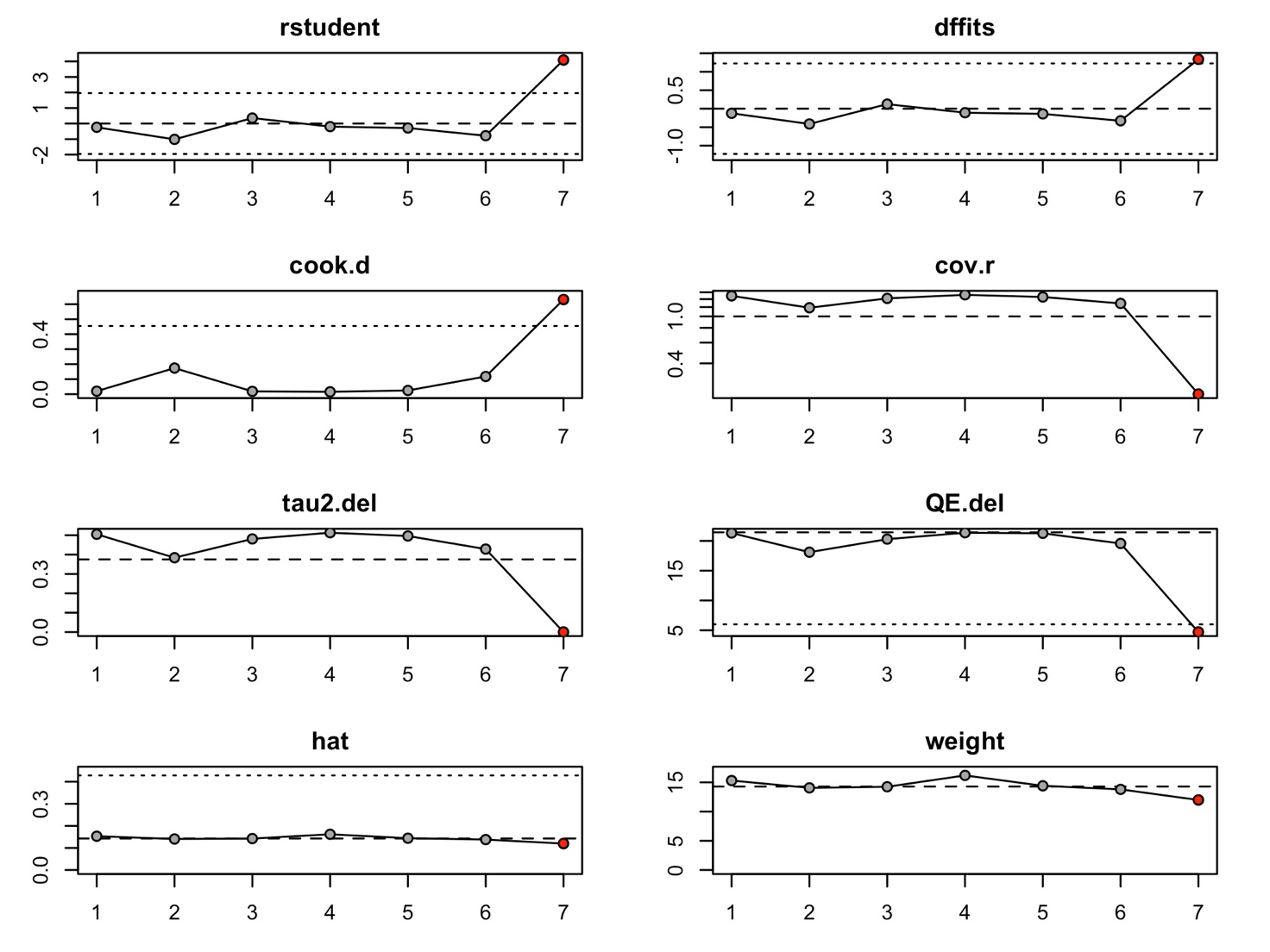
**

# Figure S25. Influence diagnostics for the meta-analysis of post-pre changes between groups for fasting insulin.

This figure summarizes influence statistics across eight diagnostic plots, each assessing how individual studies affect the overall model estimates and heterogeneity. Abbreviations: rstudent = standardized residuals; cook.d = Cook’s distance; dffits = difference in fits; cov.r = covariance ratio; tau2.del = change in between-study variance (τ²); QE.del = change in heterogeneity statistic (Q); hat = leverage; weight = study weight in the model.

**
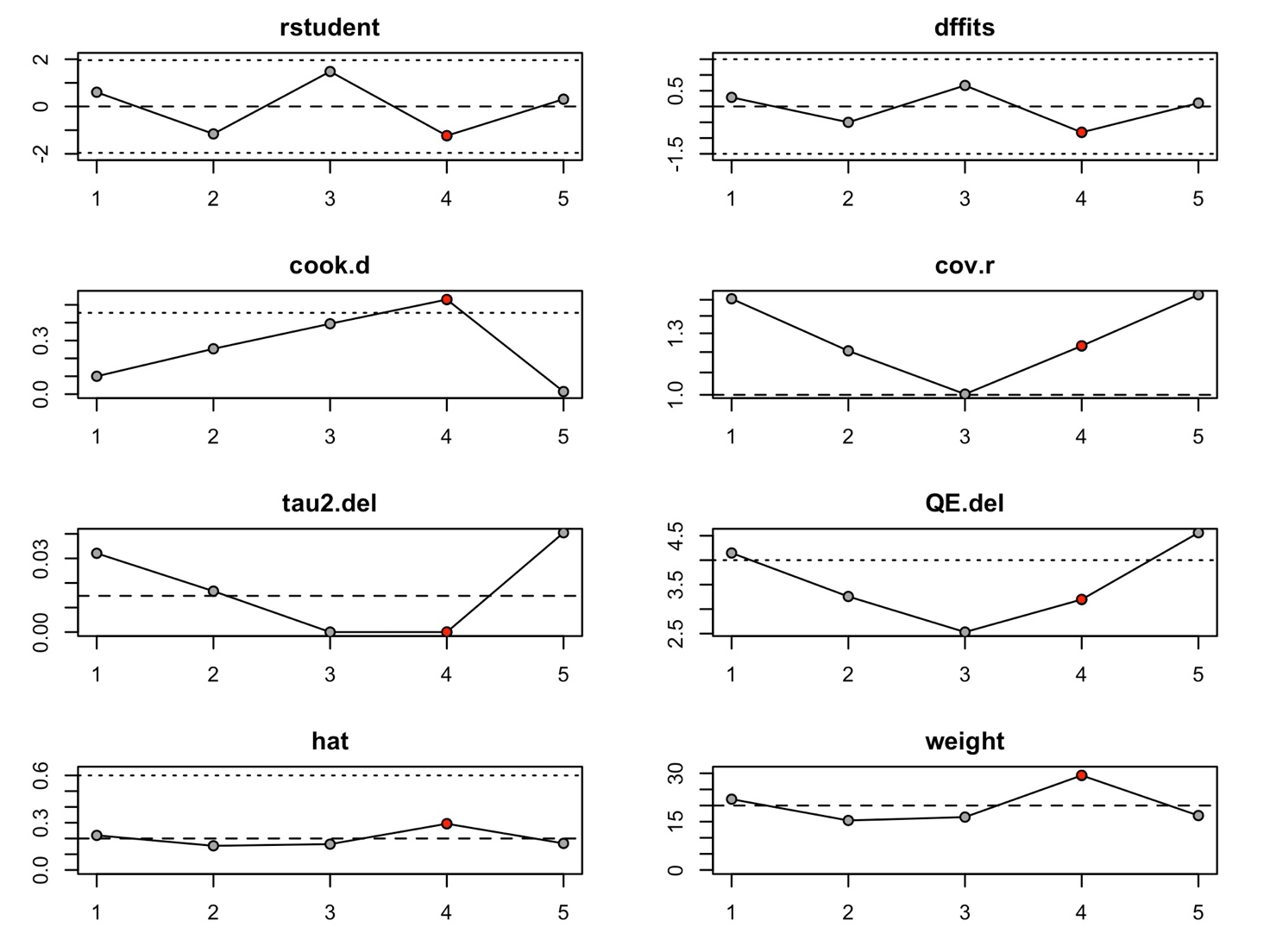
**

# Figure S26. Influence diagnostics for the meta-analysis of post-pre changes between groups for homeostatic model assessment of insulin resistance.

This figure summarizes influence statistics across eight diagnostic plots, each assessing how individual studies affect the overall model estimates and heterogeneity. Abbreviations: rstudent = standardized residuals; cook.d = Cook’s distance; dffits = difference in fits; cov.r = covariance ratio; tau2.del = change in between-study variance (τ²); QE.del = change in heterogeneity statistic (Q); hat = leverage; weight = study weight in the model.

**
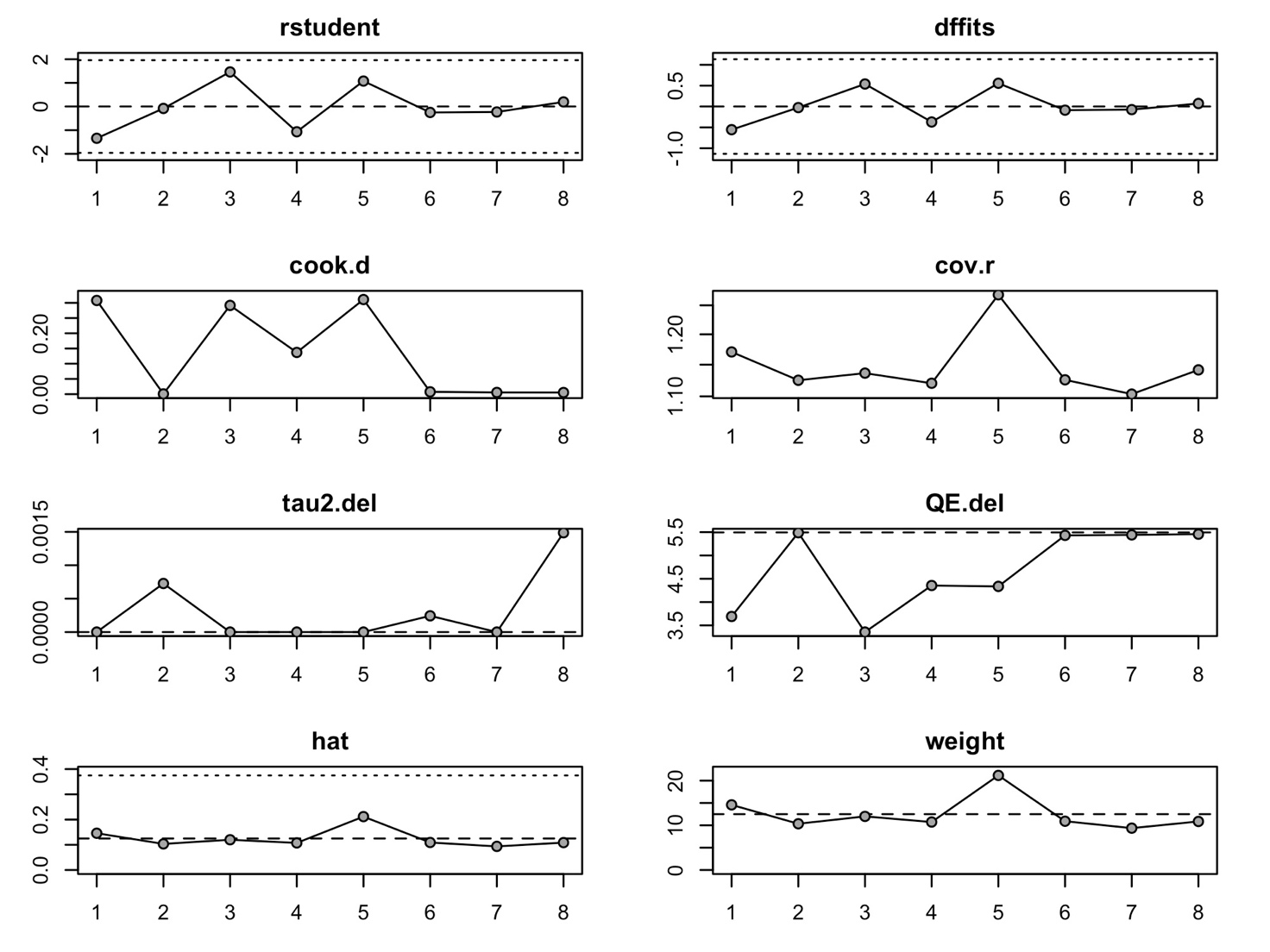
**

# Figure S27. Influence diagnostics for the meta-analysis of post-pre changes between groups for low-density lipoprotein (LDL-C).

This figure summarizes influence statistics across eight diagnostic plots, each assessing how individual studies affect the overall model estimates and heterogeneity. Abbreviations: rstudent = standardized residuals; cook.d = Cook’s distance; dffits = difference in fits; cov.r = covariance ratio; tau2.del = change in between-study variance (τ²); QE.del = change in heterogeneity statistic (Q); hat = leverage; weight = study weight in the model.

**
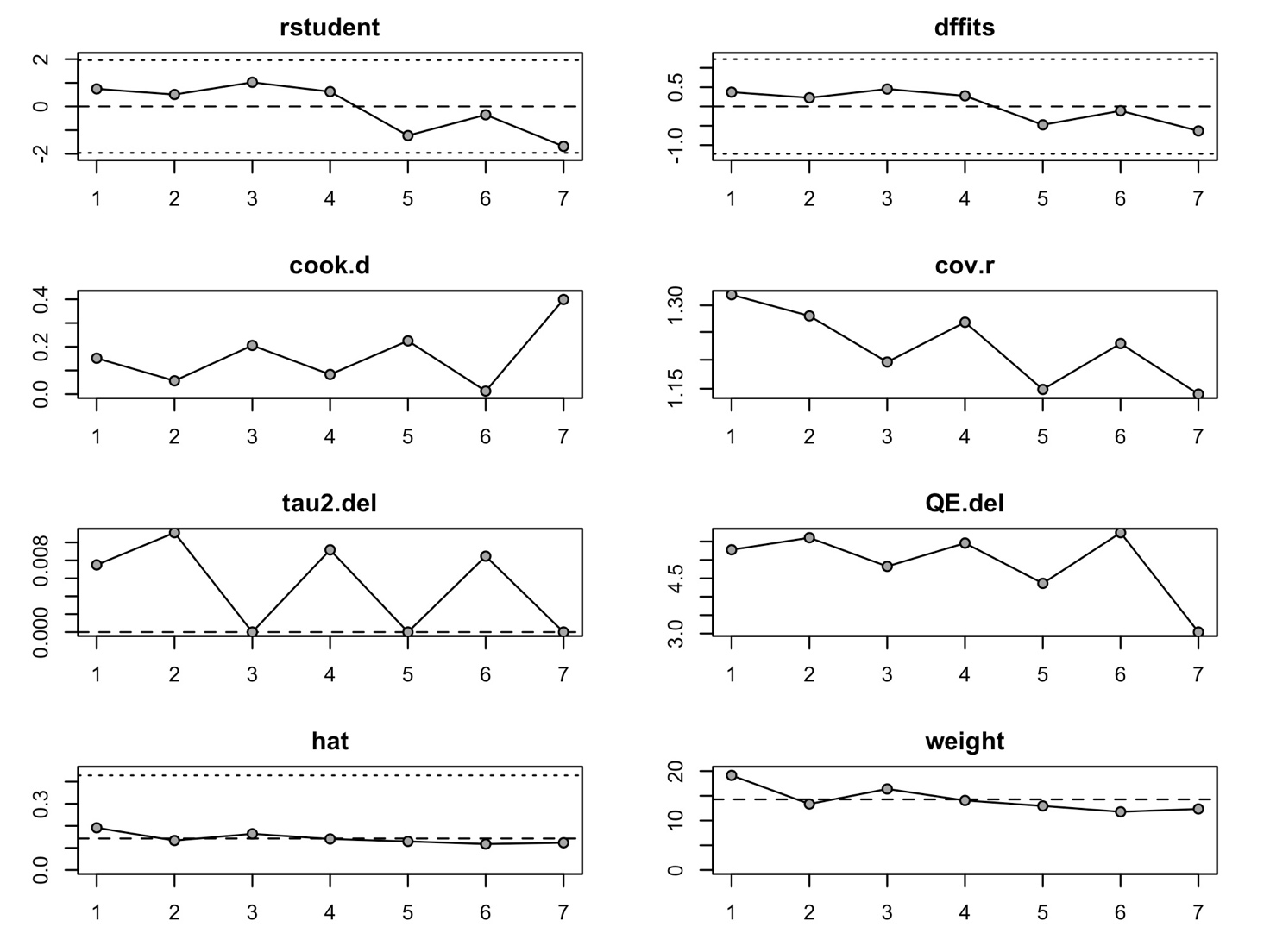
**

# Figure S28. Influence diagnostics for the meta-analysis of post-pre changes between groups for high-density lipoprotein (HDL-C).

This figure summarizes influence statistics across eight diagnostic plots, each assessing how individual studies affect the overall model estimates and heterogeneity. Abbreviations: rstudent = standardized residuals; cook.d = Cook’s distance; dffits = difference in fits; cov.r = covariance ratio; tau2.del = change in between-study variance (τ²); QE.del = change in heterogeneity statistic (Q); hat = leverage; weight = study weight in the model.

**
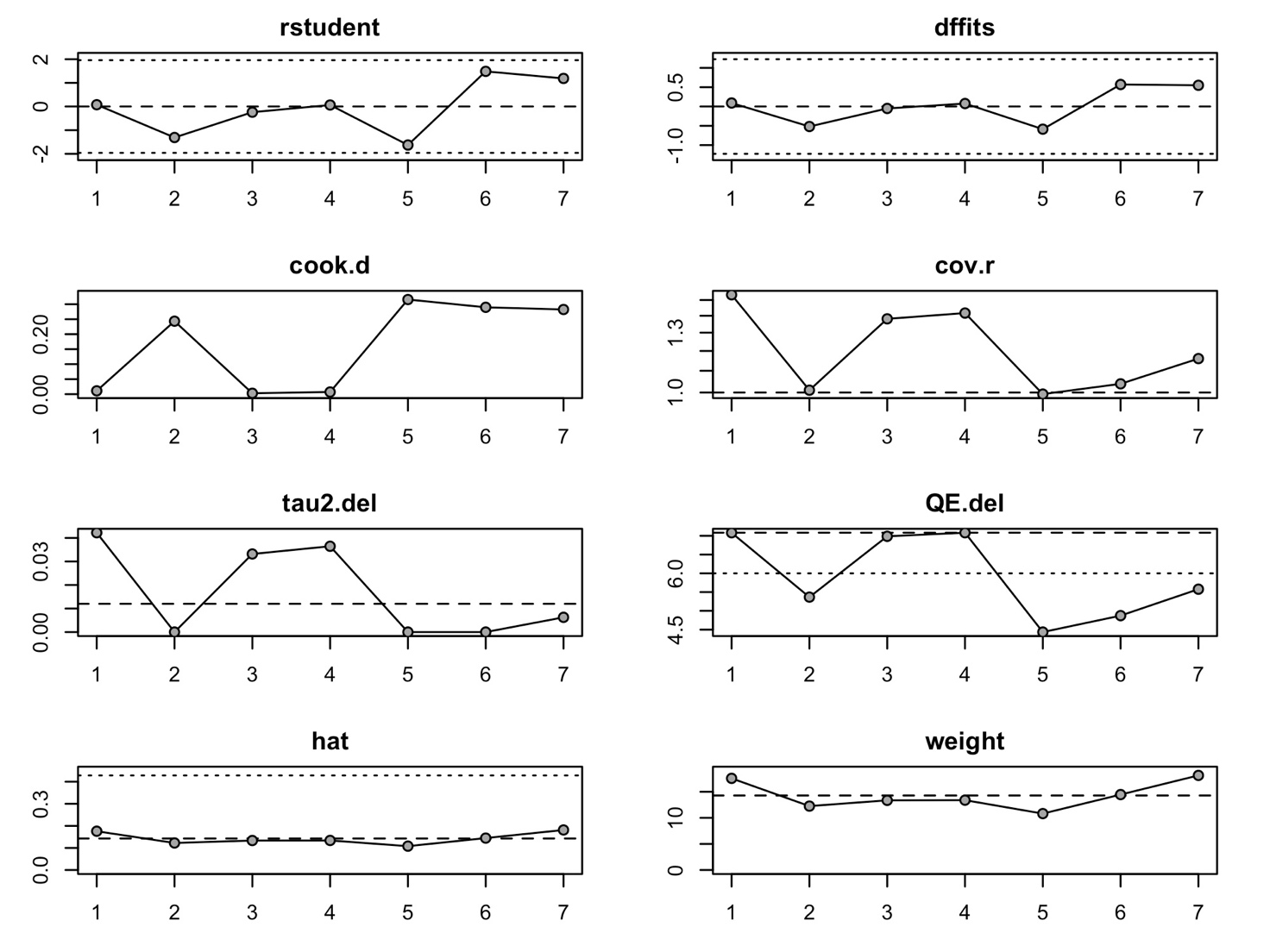
**

# Figure S29. Influence diagnostics for the meta-analysis of post-pre changes between groups for total cholesterol.

This figure summarizes influence statistics across eight diagnostic plots, each assessing how individual studies affect the overall model estimates and heterogeneity. Abbreviations: rstudent = standardized residuals; cook.d = Cook’s distance; dffits = difference in fits; cov.r = covariance ratio; tau2.del = change in between-study variance (τ²); QE.del = change in heterogeneity statistic (Q); hat = leverage; weight = study weight in the model.

**
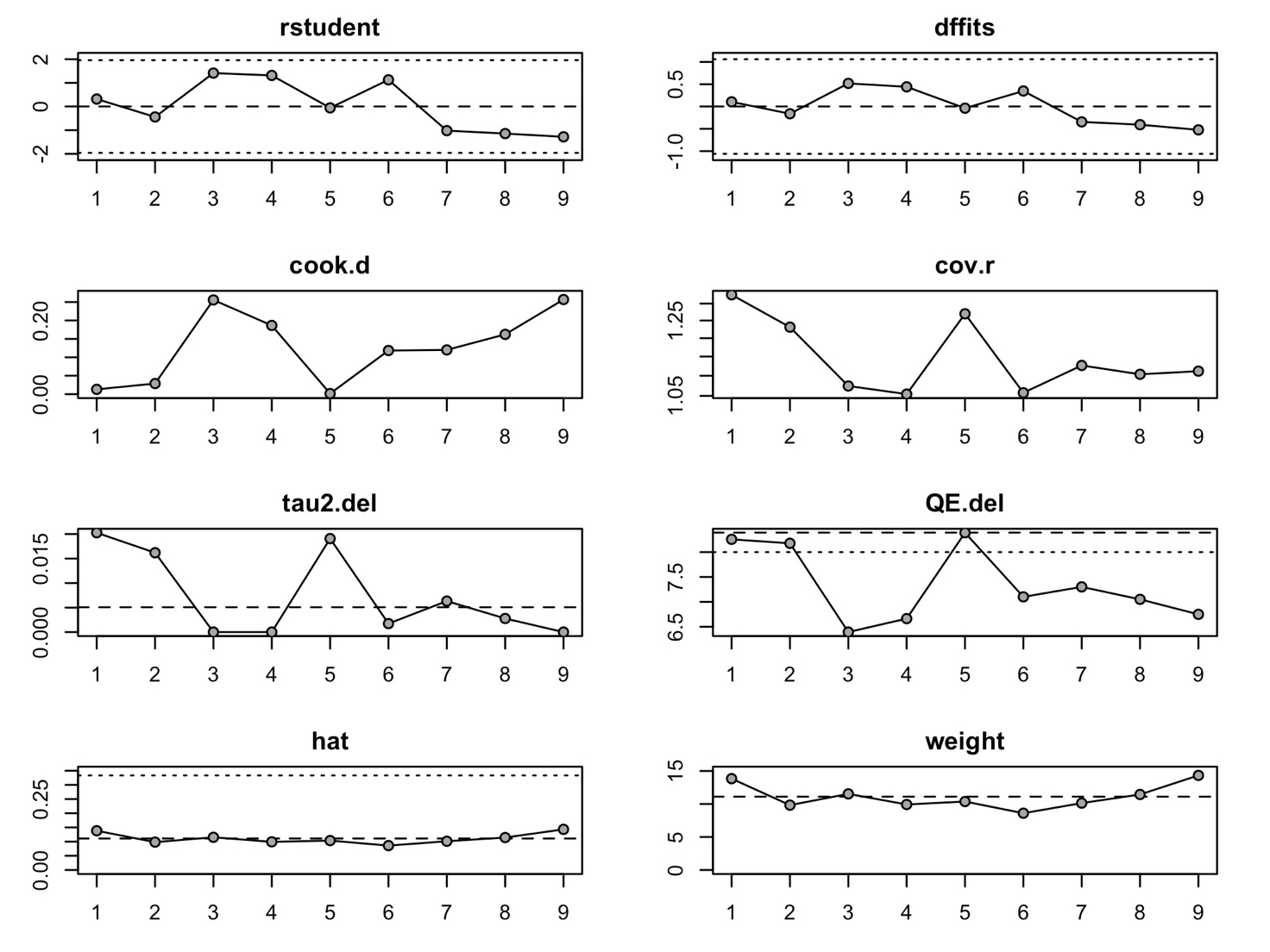
**

# Figure S30 Influence diagnostics for the meta-analysis of post-pre changes between groups for triglycerides.

This figure summarizes influence statistics across eight diagnostic plots, each assessing how individual studies affect the overall model estimates and heterogeneity. Abbreviations: rstudent = standardized residuals; cook.d = Cook’s distance; dffits = difference in fits; cov.r = covariance ratio; tau2.del = change in between-study variance (τ²); QE.del = change in heterogeneity statistic (Q); hat = leverage; weight = study weight in the model.

**
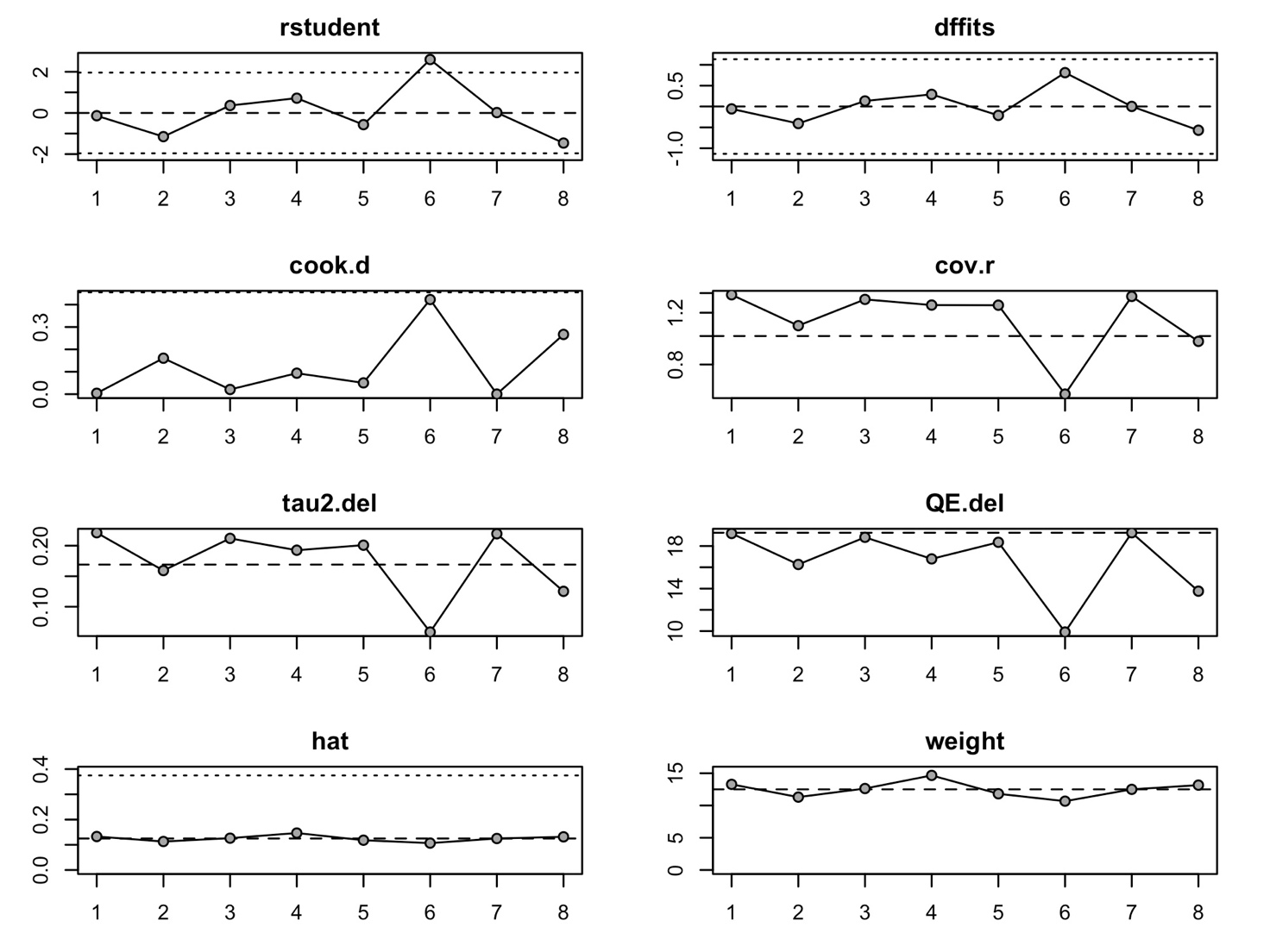
**

# Figure S31. Influence diagnostics for the meta-analysis of weight exponents for post-pre changes between groups for systolic blood pressure (SBP).

This figure summarizes influence statistics across eight diagnostic plots, each assessing how individual studies affect the overall model estimates and heterogeneity. Abbreviations: rstudent = standardized residuals; cook.d = Cook’s distance; dffits = difference in fits; cov.r = covariance ratio; tau2.del = change in between-study variance (τ²); QE.del = change in heterogeneity statistic (Q); hat = leverage; weight = study weight in the model.

**
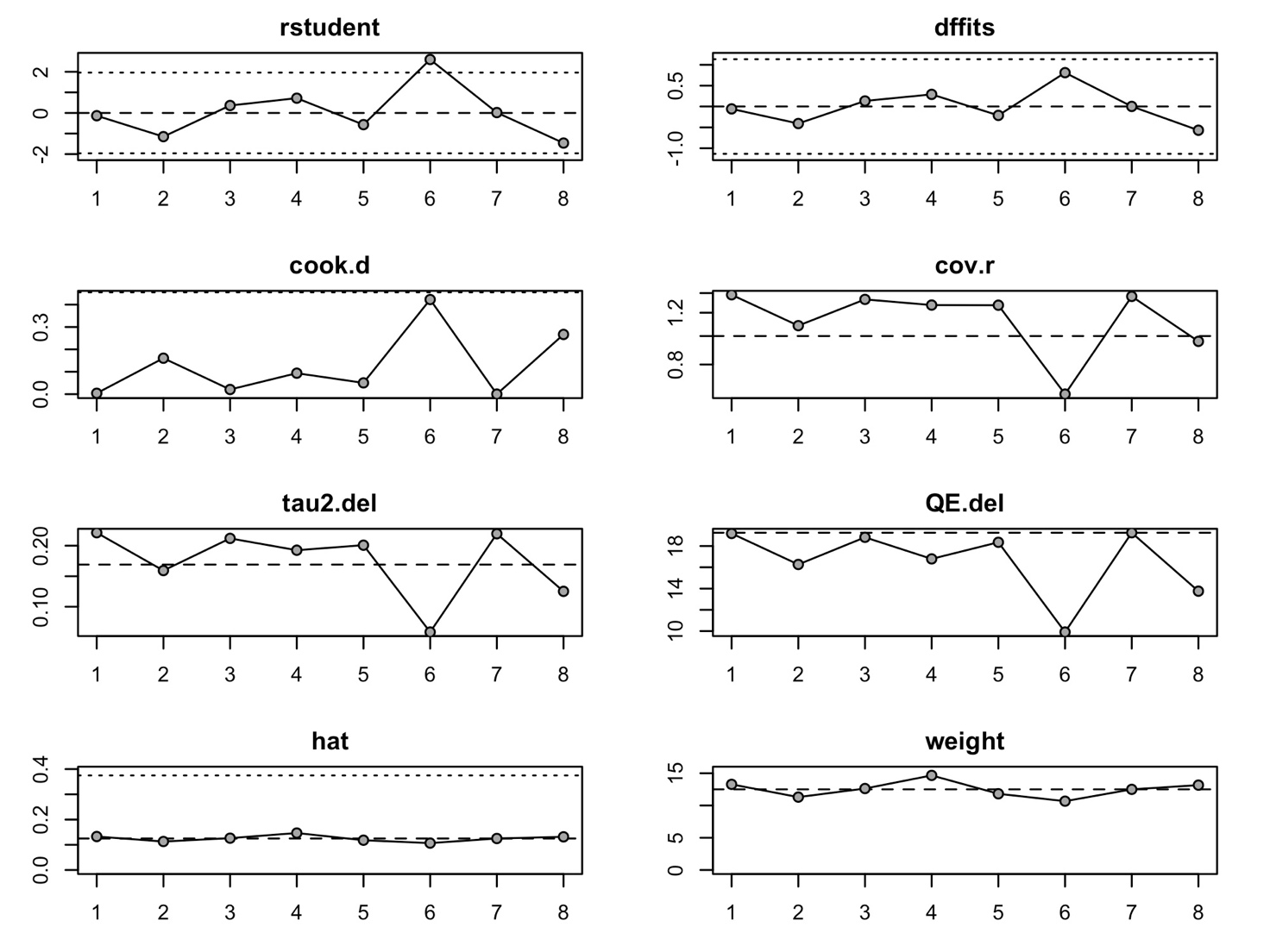
**

# Figure S32. Influence diagnostics for the meta-analysis of weight exponents for post-pre changes between groups for diastolic blood pressure (DBP).

This figure summarizes influence statistics across eight diagnostic plots, each assessing how individual studies affect the overall model estimates and heterogeneity. Abbreviations: rstudent = standardized residuals; cook.d = Cook’s distance; dffits = difference in fits; cov.r = covariance ratio; tau2.del = change in between-study variance (τ²); QE.del = change in heterogeneity statistic (Q); hat = leverage; weight = study weight in the model.

# Table S1. PRISMA checklist.

| **Section/topic** | **#** | **Checklist item** | **Reported on page #** |
| --- | --- | --- | --- |
| **TITLE** | | |  |
| Title | 1 | Identify the report as a systematic review, meta-analysis, or both. | 1 |
| **ABSTRACT** | | |  |
| Structured summary | 2 | Provide a structured summary including, as applicable: background; objectives; data sources; study eligibility criteria, participants, and interventions; study appraisal and synthesis methods; results; limitations; conclusions and implications of key findings; systematic review registration number. | 2 |
| **INTRODUCTION** | | |  |
| Rationale | 3 | Describe the rationale for the review in the context of what is already known. | 3 |
| Objectives | 4 | Provide an explicit statement of questions being addressed with reference to participants, interventions, comparisons, outcomes, and study design (PICOS). | 3-4 |
| **METHODS** | | |  |
| Protocol and registration | 5 | Indicate if a review protocol exists, if and where it can be accessed (e.g., Web address), and, if available, provide registration information including registration number. | 5 |
| Eligibility criteria | 6 | Specify study characteristics (e.g., PICOS, length of follow-up) and report characteristics (e.g., years considered, language, publication status) used as criteria for eligibility, giving rationale. | 5 |
| Information sources | 7 | Describe all information sources (e.g., databases with dates of coverage, contact with study authors to identify additional studies) in the search and date last searched. | 5 |
| Search | 8 | Present full electronic search strategy for at least one database, including any limits used, such that it could be repeated. | S3 |
| Study selection | 9 | State the process for selecting studies (i.e., screening, eligibility, included in systematic review, and, if applicable, included in the meta-analysis). | 5-6 |
| Data collection process | 10 | Describe method of data extraction from reports (e.g., piloted forms, independently, in duplicate) and any processes for obtaining and confirming data from investigators. | 6 |
| Data items | 11 | List and define all variables for which data were sought (e.g., PICOS, funding sources) and any assumptions and simplifications made. | 5 |
| Risk of bias in individual studies | 12 | Describe methods used for assessing risk of bias of individual studies (including specification of whether this was done at the study or outcome level), and how this information is to be used in any data synthesis. | 6 |
| Summary measures | 13 | State the principal summary measures (e.g., risk ratio, difference in means). | 6-7 |
| Synthesis of results | 14 | Describe the methods of handling data and combining results of studies, if done, including measures of consistency (e.g., I^2^) for each meta-analysis. | 6-7 |
| Risk of bias across studies | 15 | Specify any assessment of risk of bias that may affect the cumulative evidence (e.g., publication bias, selective reporting within studies). | 6 |
| Additional analyses | 16 | Describe methods of additional analyses (e.g., sensitivity or subgroup analyses, meta-regression), if done, indicating which were pre-specified. | 7 |
| **RESULTS** | | |  |
| Study selection | 17 | Give numbers of studies screened, assessed for eligibility, and included in the review, with reasons for exclusions at each stage, ideally with a flow diagram. | 8 |
| Study characteristics | 18 | For each study, present characteristics for which data were extracted (e.g., study size, PICOS, follow-up period) and provide the citations. | 11-15 |
| Risk of bias within studies | 19 | Present data on risk of bias of each study and, if available, any outcome level assessment (see item 12). | 10 |
| Results of individual studies | 20 | For all outcomes considered (benefits or harms), present, for each study: (a) simple summary data for each intervention group (b) effect estimates and confidence intervals, ideally with a forest plot. | 17-21 |
| Synthesis of results | 21 | Present results of each meta-analysis done, including confidence intervals and measures of consistency. | 17-21 |
| Risk of bias across studies | 22 | Present results of any assessment of risk of bias across studies (see Item 15). | 10 |
| Additional analysis | 23 | Give results of additional analyses, if done (e.g., sensitivity or subgroup analyses, meta-regression [see Item 16]). | S7-S9 |
| **DISCUSSION** | | |  |
| Summary of evidence | 24 | Summarize the main findings including the strength of evidence for each main outcome; consider their relevance to key groups (e.g., healthcare providers, users, and policy makers). | 22-23 |
| Limitations | 25 | Discuss limitations at study and outcome level (e.g., risk of bias), and at review-level (e.g., incomplete retrieval of identified research, reporting bias). | 22-24 |
| Conclusions | 26 | Provide a general interpretation of the results in the context of other evidence, and implications for future research. | 24-25 |
| **FUNDING** | | |  |
| Funding | 27 | Describe sources of funding for the systematic review and other support (e.g., supply of data); role of funders for the systematic review. | n/a |

# Table S2. Search strategy employed for each database.

| Database | Search Terms | Date Range |
| --- | --- | --- |
| Pubmed/Medline | ("adult*" OR "young adult*" OR "mature" OR "early adulthood")  AND ("hypoxi*" OR "hypoxic training" OR "hypoxic exercise" OR "intermittent hypoxia"  OR "altitude training" OR "low oxygen" OR "oxygen deficiency" OR "reduced oxygen"  OR "normobaric hypoxia" OR "hypobaric hypoxia" OR "high altitude training"  OR "simulated altitude" OR "hypoxic conditioning")  AND ("cardiovascular" OR "heart" OR "circulatory" OR "metabolic" OR "metabolism"  OR "insulin resistance" OR "blood pressure" OR "glucose" OR "glucose metabolism"  OR "lipid profile" OR "cholesterol" OR "triglycerides" OR "hypertension" OR "hyperlipidemia"  OR "blood sugar" OR "lipid metabolism" OR "dyslipidemia" OR "HOMA" OR "fasting insulin"  OR "fasting glucose" OR "glycemic profile" OR "glycemic homeostasis" OR "glucose homeostasis")  AND ("overweight" OR "obesity" OR "BMI" OR "body mass index" OR "weight gain"  OR "adiposity" OR "excess weight" OR "high BMI" OR "fat" OR "fat mass" OR "body weight"  OR "body fat" OR "body composition" OR "weight control" OR "weight management"  OR "weight loss" OR "fat percentage" OR "waist circumference" OR "lean mass"  OR "waist-to-hip ratio") | 1990-01-01 to 2025-06-23 |
| Web of Science | TS=("adult*" OR "young adult*" OR "mature" OR "early adulthood")  AND TS=("hypoxi*" OR "hypoxic training" OR "hypoxic exercise" OR "intermittent hypoxia"  OR "altitude training" OR "low oxygen" OR "oxygen deficiency" OR "reduced oxygen"  OR "normobaric hypoxia" OR "hypobaric hypoxia" OR "high altitude training"  OR "simulated altitude" OR "hypoxic conditioning")  AND TS=("cardiovascular" OR "heart" OR "circulatory" OR "metabolic" OR "metabolism"  OR "insulin resistance" OR "blood pressure" OR "glucose" OR "glucose metabolism"  OR "lipid profile" OR "cholesterol" OR "triglycerides" OR "hypertension" OR "hyperlipidemia"  OR "blood sugar" OR "lipid metabolism" OR "dyslipidemia" OR "HOMA" OR "fasting insulin"  OR "fasting glucose" OR "glycemic profile" OR "glycemic homeostasis" OR "glucose homeostasis")  AND TS=("overweight" OR "obesity" OR "BMI" OR "body mass index" OR "weight gain"  OR "adiposity" OR "excess weight" OR "high BMI" OR "fat" OR "fat mass" OR "body weight"  OR "body fat" OR "body composition" OR "weight control" OR "weight management"  OR "weight loss" OR "fat percentage" OR "waist circumference" OR "lean mass"  OR "waist-to-hip ratio") | 1990-01-01 to 2025-06-23 |
| Cochrane | ("adult" OR "young adult" OR "mature" OR "early adulthood")  AND ("hypoxia" OR "hypoxic training" OR "hypoxic exercise" OR "intermittent hypoxia"  OR "altitude training" OR "low oxygen" OR "oxygen deficiency" OR "reduced oxygen"  OR "normobaric hypoxia" OR "hypobaric hypoxia" OR "high altitude training"  OR "simulated altitude" OR "hypoxic conditioning")  AND ("cardiovascular" OR "heart" OR "circulatory" OR "metabolic" OR "metabolism"  OR "insulin resistance" OR "blood pressure" OR "glucose" OR "glucose metabolism"  OR "lipid profile" OR "cholesterol" OR "triglycerides" OR "hypertension" OR "hyperlipidemia"  OR "blood sugar" OR "lipid metabolism" OR "dyslipidemia" OR "HOMA" OR "fasting insulin"  OR "fasting glucose" OR "glycemic profile" OR "glycemic homeostasis" OR "glucose homeostasis")  AND ("overweight" OR "obesity" OR "BMI" OR "body mass index" OR "weight gain"  OR "adiposity" OR "excess weight" OR "high BMI" OR "fat" OR "fat mass" OR "body weight"  OR "body fat" OR "body composition" OR "weight control" OR "weight management"  OR "weight loss" OR "fat percentage" OR "waist circumference" OR "lean mass"  OR "waist-to-hip ratio") | 1990-01-01 to 2025-06-23 |

# Table S3. Equations used for the calculation of effect sizes.

|  | | **Between groups differences** | | **Within groups differences** | |
| --- | --- | --- | --- | --- | --- |
| **Standardized mean difference** | $SMD=c({df}_{E,C})\cdot\left[ \frac{\left( \bar{X}_{pos,E} - \bar{X}_{pre,E} \right)-\left( \bar{X}_{pos,C}-\bar{X}_{pre,C} \right)}{S_{pool}} \right]$ | | $d=c(df)\cdot\left[ \frac{\left( \bar{X}_{pos,E} - \bar{X}_{pre,E} \right)}{S_{pre}} \right]$ | |  |
| **Pooled standard deviation** | $\bar{S}_{pool}=\sqrt{\frac{(n_{c} - 1)\cdot S_{pre,c}^{2} + (n_{e} - 1)\cdot S_{pre,e}^{2}}{n_{c} + n_{e} - 2}}$ | |  | |  |
| **Correction factor** | $c\left( {df}_{E,C} \right)=1-\frac{3}{4\left( n_{c} + n_{e} - 2 \right) - 1}$ | | $c\left( df \right)=1-\frac{3}{4\left( n - 1 \right) - 1}$ | |  |
| **Variance^a^** | $S^{2}(d)=\left[ c\left( {df}_{E,C} \right) \right]^{2}\cdot2\left( 1-r \right)\cdot\left( \frac{n_{c} + n_{e}}{n_{c}n_{e}} \right)\cdot\left( \frac{n_{c}+n_{e}-2}{n_{c}{+ n}_{e}-4} \right)\times$  $\times\left[ 1+\frac{n_{c}n_{e} d^{2}}{{2(1-r)(n}_{c}{+ n}_{e})} \right]-d^{2}$ | | $S^{2}(d)=\left[ c\left( df \right) \right]^{2}\cdot\left[ \frac{2\cdot\left( 1-r \right)}{n} \right]\cdot\left( \frac{n-1}{n-3} \right)\cdot\left[ 1+\frac{n\cdot d^{2}}{2\cdot\left( 1-r \right)} \right]-d^{2}$ | |  |

_C_ = control group; _E_ = experimental group.

^a^ *r* = 0.7, according to the standard *r* value recommended by Rosenthal [1].

1. Rosenthal R. Meta-analytic procedures for social research. Newbury Park, CA: Sage 1991.

# Table S4. Pre and post interventions values of glucose homeostasis of the studies included in the meta-analysis.

| Study | Fasting glucose (mg/dL) | | | | Fasting Insulin (mU/L) | | | | HOMA-IR | | | |
| --- | --- | --- | --- | --- | --- | --- | --- | --- | --- | --- | --- | --- |
|  | **Hypoxia** | | **Normoxia** | | **Hypoxia** | | **Normoxia** | | **Hypoxia** | | **Normoxia** | |
|  | Pre | Post | Pre | Post | Pre | Post | Pre | Post | Pre | Post | Pre | Post |
| Ghait et al., 2020 | 104.5 + 14.41 | 100.9 + 16.22 | 104.5 + 16.22 | 117.12 + 34.23 | 11.6 + 9.6 | 12.5 + 8.7 | 10.5 + 4.9 | 11.1 + 3.8 | 1.5 + 1.2 | 1.7 + 1.1 | 1.4 + 0.6 | 1.5 + 0.5 |
| Jung et al., 2020 | 86.8 + 8.5 | 101.8 + 17.3 | 103.2 + 17.9 | 94.6 + 8.6 | 8.0 + 3.1 | 7.4 + 2.9 | 5.4 + 2.0 | 6.1 + 2.6 | 2.1 + 1.0 | 1.9 + 0.9 | 1.2 + 0.5 | 1.4 + 0.7 |
| Gatterer et al., 2015 | 117.3 + 39.0 | 123.0 + 41.3 | 123.0 + 41.3 | 111.7 + 16.8 | **-** | **-** | **-** | **-** | **-** | **-** | **-** | **-** |
| Fernandez-Menendez et al. 2018 | 90.09 + 5.41 | 88.29 + 9.01 | 91.89 + 5.41 | 93.69 + 5.41 | 13.8 + 7.2 | 14.8 + 8.5 | 16.1 + 10.0 | 13.0 + 5.6 | 1.8 + 0.9 | 1.9 + 1.1 | 2.0 + 1.2 | 1.7 + 0.7 |
| Wiesner et al., 2009 | **-** | **-** | **-** | **-** | 8.4 + 1.3 | 5.3 + 0.7 | 9.7 + 1.5 | 6.5 + 0.7 | 1.9 + 0.3 | 1.1 + 0.2 | 2.1 + 0.3 | 1.4 + 0.2 |
| Chacaroun et al., 2020 | 104.5 + 18.02 | 104.5 + 12.61 | 104.5 + 14.41 | 104.5 + 14.41 | 9.7 + 4.2 | 9.3 + 4.1 | 11.7 + 4.5 | 11.3 + 6.9 | 1.30 + 0.57 | 1.27 + 0.49 | 1.55 + 0.59 | 1.5 + 0.92 |
| Morishima et al., 2020 | 100.0 + 2.0 | 92.0 + 3 | 100.0 + 3.0 | 93.0 + 2.0 | 10.0 + 2.0 | 7.8 + 1.6 | 4.7 + 0.6 | 3.0 + 0.2 | **-** | **-** | **-** | **-** |
| Klug et al., 2018 | 106.31 + 7.21 | 106.31 + 5.41 | 109.91 + 9.01 | 106.31 + 7.21 | 15.0 + 2.0 | 18.0 + 3 | 19.0 + 3.0 | 17.0 + 4.0 | **-** | **-** | **-** | **-** |
| Camacho-Cardenosa et al., 2018a | 108.14 + 8.45 | 105.21 + 8.72 | 105.28 + 6.32 | 102.07 + 5.69 | **-** | **-** | **-** | **-** | **-** | **-** | **-** | **-** |
| Camacho-Cardenosa et al., 2018b | 104.47 + 7.56 | 103.64 + 7.91 | 109.43 + 14.39 | 100.4 + 7.46 | **-** | **-** | **-** | **-** | **-** | **-** | **-** | **-** |

**Note:**  All the values are reported as mean + standard deviation. HOMA-IR, homeostatic model assessment for insulin resistance.

# Table S5. Pre and post interventions values of lipids profile of the studies included in the meta-analysis.

| Study | LDL (mg/dL) | | | | HDL (mg/dL) | | | | Total-cholesterol (mg/dL) | | | | Triglycerides (mg/dL) | | | |
| --- | --- | --- | --- | --- | --- | --- | --- | --- | --- | --- | --- | --- | --- | --- | --- | --- |
|  | **Hypoxia** | | **Normoxia** | | **Hypoxia** | | **Normoxia** | | **Hypoxia** | | **Normoxia** | | **Hypoxia** | | **Normoxia** | |
|  | **Pre** | **Post** | **Pre** | **Post** | **Pre** | **Post** | **Pre** | **Post** | **Pre** | **Post** | **Pre** | **Post** | **Pre** | **Post** | **Pre** | **Post** |
| Ghait et al., 2020 | 120 + 40 | 110 + 20 | 120 + 30 | 120 + 30 | 40 + 10 | 40 + 10 | 40 + 10 | 40 + 10 | 200 + 40 | 200 + 40 | 200 + 40 | 200 + 40 | 160 + 100 | 210 + 190 | 150 + 70 | 190 + 30 |
| Jung et al., 2020 | 151.7 + 52.4 | 142.0 + 61.9 | 116.9 + 27.2 | 104.9 + 17.7 | 57.5 + 14.6 | 56.4 + 15.3 | 61.3 + 7.5 | 60.6 + 11.0 | 228.4 + 58.6 | 203.4 + 45.2 | 191.0 + 26.7 | 187.2 + 20.4 | 135.2 + 41.7 | 114.8 + 43.5 | 98.4 + 28.6 | 83 + 18 |
| Gatterer et al., 2015 | 206.9 + 29.7 | 213.8 + 33.0 | 208.7 + 37.5 | 197.7 + 37.7 | 49.4 + 14.3 | 50.4 + 13.0 | 53.1 + 16.0 | 52.7 + 13.3 | **-** | **-** | **-** | **-** | 210.5 + 100.2 | 224.3 + 105 | 176.1 + 62.0 | 154.1 + 51.6 |
| Fernandez-Menendez et al. 2018 | 111.97 + 30.94 | 104.25 + 27.03 | 104.25 + 27.03 | 104.25 + 34.75 | 54.05 + 15.44 | 50.19 + 15.44 | 57.92 + 11.58 | 54.05 + 11.58 | 193.05 + 27.03 | 185.33 + 30.89 | 196.61 + 42.47 | 193.05 + 42.47 | 141.59 + 79.95 | 168.14 + 115.04 | 159.29 + 115.04 | 141.59 + 79.65 |
| Wiesner et al., 2009 | 115.0 + 5.1 | 112.0 + 6.1 | 121.0 + 7.8 | 116.0 + 9.8 | **-** | **-** | **-** | **-** | **-** | **-** | **-** | **-** | **-** | **-** | **-** | **-** |
| Chacaroun et al., 2020 | 142 + 30 | 142 + 30 | 115 + 44 | 115 + 38 | 53 + 19 | 46 + 8 | 50 + 13 | 53 + 17 | 210 + 30 | 210 + 30 | 190 + 40 | 190 + 40 | 130 + 30 | 130 + 30 | 150 + 30 | 150 + 100 |
| Morishima et al., 2020 | 93 + 8 | 90 + 10 | 95 + 6 | 92 + 6 | 52 + 3 | 49 + 4 | 52 + 3 | 50 + 3 | 170 + 11 | 161 + 11 | 162 + 9 | 159 + 9 | 123 + 32 | 105 + 21 | 92 + 13 | 64 + 6 |
| Klug et al., 2018 | 147 + 14 | 134 + 14 | 129 + 14 | 114 + 10 | 52 + 5 | 47 + 4 | 43 + 2 | 41 + 2 | **-** | **-** | **-** | **-** | 218 + 59 | 163 + 25 | 228 + 26 | 188 + 23 |
| Camacho-Cardenosa et al., 2018a | **-** | **-** | **-** | **-** | **-** | **-** | **-** | **-** | 194.21 + 31.59 | 199.86 + 25.24 | 188.21 + 30.87 | 180.29 + 33.58 | 73.78 + 21.52 | 53.5 + 16.79 | 78.84 + 23.94 | 66.15 + 20.77 |
| Camacho-Cardenosa et al., 2018b | **-** | **-** | **-** | **-** | **-** | **-** | **-** | **-** | 191.36 + 66.01 | 199.14 + 46.00 | 200.00 + 52.05 | 189.64 + 25.24 | 74.27 + 29.2 | 56.09 + 22.13 | 73.14 + 21.16 | 63.21 + 22.31 |

**Note:**  All the values are reported as mean + standard deviation. LDL, low-density lipoprotein; HDL, high-density lipoprotein.

# Table S6. Pre and post interventions values of blood pressure of the studies included in the meta-analysis.

| Study | Systolic blood pressure (mmHg) | | | | Diastolic blood pressure (mmHg) | | | |
| --- | --- | --- | --- | --- | --- | --- | --- | --- |
|  | **Hypoxia** | | **Normoxia** | | **Hypoxia** | | **Normoxia** | |
|  | Pre | Post | Pre | Post | Pre | Post | Pre | Post |
| Ghait et al., 2020 | 128.4 + 12.7 | 125.5 + 12.5 | 134.3 + 13.7 | 132.3 + 12.3 | 87.2 + 9.8 | 83.2 + 10.4 | 86.0 + 11.2 | 84.3 + 7.5 |
| Jung et al., 2020 | 122 + 13.1 | 116.3 + 10.5 | 119.1 + 9.8 | 120.4 + 11.1 | 77.3 + 9.5 | 71.8 + 6.3 | 72.2 + 10.3 | 71.2 + 9.5 |
| Gatterer et al., 2015 | 134.2 + 9.7 | 129.5 + 11.5 | 125.8 + 15.2 | 118.6 + 14.3 | 91.7 + 11.0 | 89.8 + 8.2 | 86.8 + 9.4 | 82.4 + 7.8 |
| Fernandez-Menendez et al. 2018 | **-** | **-** | **-** | **-** | **-** | **-** | **-** | **-** |
| Wiesner et al., 2009 | 128 + 3 | 126.0 + 2.6 | 129 + 2.9 | 126.0 + 2.5 | 83.0 + 2.1 | 80.0 + 1.7 | 80.0 + 2.2 | 79.0 + 1.7 |
| Chacaroun et al., 2020 | 131 + 11 | 128 + 13 | 120 + 15 | 121 + 10 | 85 + 9 | 80 + 8 | 75 + 10 | 73 + 7 |
| Morishima et al., 2020 | **-** | **-** | **-** | **-** | **-** | **-** | **-** | **-** |
| Klug et al., 2018 | 145 + 3 | 137 + 3 | 135 + 4 | 123 + 5 | 81 + 2 | 79 + 3 | 83 + 3 | 77 + 3 |
| Camacho-Cardenosa et al., 2018a | 119.86 + 10.8 | 115.21 + 14.01 | 116.36 + 11.91 | 111.57 + 13.54 | 74.86 + 11.34 | 74.64 + 6.38 | 73.07 + 8.66 | 71.43 + 10.40 |
| Camacho-Cardenosa et al., 2018b | 114.31 + 12.34 | 111.46 + 11.51 | 108.27 + 12.89 | 113.4 + 12.54 | 70.08 + 6.54 | 71.00 + 8.02 | 71.13 + 8.34 | 70.6 + 8.95 |

**Note:**  All the values are reported as mean + standard deviation.

# Table S7. Moderation analysis for all the outcomes considering altitude and the baseline level.

|  | Altitude | | Baseline level | |
| --- | --- | --- | --- | --- |
|  | Estimate (95% CI) | *p*-value | Estimate (95% CI) | *p*-value |
| Fasting glucose | 0.000 (-0.001; 0.000) | 0.143 | 0.049 (0.008; 0.090) | **0.020** |
| Fasting insulin | 0.000 (-0.001; 0.000) | 0.193 | 0.167 (0.088; 0.246) | **< 0.001** |
| HOMA - IR | 0.001 (-0.001; 0.002) | 0.322 | 1.040 (-1.901; 3.982) | 0.488 |
| LDL - C | 0.000 (-0.001; 0.000) | 0.197 | 0.006 (-0.001; 0.073) | 0.073 |
| HDL - C | 0.000 (0.000; 0.001) | 0.176 | 0.023 (-0.026; 0.072) | 0.355 |
| Total cholesterol | 0.000 (-0.001; 0.000) | 0.606 | 0.010 (-0.018; 0.038) | 0.355 |
| Triglycerides | 0.000 (0.000; 0.001) | 0.362 | 0.001 (-0.003; 0.006) | 0.540 |
| Systolic blood pressure | -0.000 (-0.001; -0.000) | **0.024** | 0.061 (0.032; 0.090) | **< 0.001** |
| Diastolic blood pressure | -0.001 (-0.002; 0.000) | 0.270 | 0.048 (-0.066; 0.161) | 0.410 |

**Note:**  All the values are reported estimate (95% confidence interval). HOMA - IR, homeostatic model assessment of insulin assessment; LDL - C, low-density lipoprotein; HDL-C, high-density lipoprotein.

# Table S8. Subgroup analysis for all the outcomes considering intervention duration.

|  | < 8 weeks | | > 8 weeks | |
| --- | --- | --- | --- | --- |
|  | Estimate (95% CI) | *p*-value | Estimate (95% CI) | *p*-value |
| Fasting glucose | -0.673 (-1.221; -0.124) | **0.016** | -0.143 (-0.637; 0.350) | 0.569 |
| Fasting insulin | 0.485 (-0.420; 1.390) | 0.294 | -0.114 (-0.470; 0.242) | 0.531 |
| HOMA - IR | -0.021 (-0.696; 0.654) | 0.952 | -0.078 (-0.434; 0.278) | 0.668 |
| LDL - C | 0.099 (-0.195; 0.393) | 0.510 | 0.058 (-0.281; 0.398) | 0.737 |
| HDL - C | -0.343 (-0.778; 0.092) | 0.122 | -0.106 (-0.413; 0.200) | 0.496 |
| Total cholesterol | -0.315 (-0.795; 0.166) | 0.199 | 0.083 (-0.184; 0.351) | 0.541 |
| Triglycerides | 0.168 (-0.322; 0.658) | 0.502 | -0.042 (-0.286; 0.202) | 0.737 |
| Systolic blood pressure | 0.660 (-0.082; 1.402) | 0.081 | -0.215 (-0.481; 0.050) | 0.112 |
| Diastolic blood pressure | 0.284 (-2.108; 2.676) | 0.816 | -0.040 (-0.284; 0.203) | 0.747 |

**Note:**  All the values are reported estimate (95% confidence interval). HOMA - IR, homeostatic model assessment of insulin assessment; LDL - C, low-density lipoprotein; HDL-C, high-density lipoprotein.

# Table S9. Subgroup analysis for all the outcomes considering intensity of the exercise.

|  | Low | | Medium | | High | |
| --- | --- | --- | --- | --- | --- | --- |
|  | Estimate (95% CI) | *p*-value | Estimate (95% CI) | *p*-value | Estimate (95% CI) | *p*-value |
| Fasting glucose | -0.927 (-1.583; -0.271) | **0.006** | 0.036 (-0.510; 0.583) | 0.896 | -0.387 (-1.006; 0.233) | 0.222 |
| Fasting insulin | -0.003 (-0.911; 0.905) | 0.995 | 0.512 (-0.799; 0.182) | 0.444 | 0.022 (-0.396; 0.440) | 0.918 |
| HOMA - IR | -0.048 (-0.870; 0.774) | 0.908 | -0.327 (-0.789; 0.134) | 0.165 | 0.073 (-0.346; 0.491) | 0.908 |
| LDL - C | -0.105 (-0.567; 0.357) | 0.657 | 0.262 (-0.029; 0.552) | 0.077 | -0.157 (-0.577; 0.263) | 0.465 |
| HDL - C | -0.015 (-0.475; 0.445) | 0.948 | -0.302 (-0.791; 0.17) | 0.227 | -0.261 (-0.833; 0.310) | 0.370 |
| Total cholesterol | -0.262 (-0.727; 0.204) | 0.270 | -0.060 (-1.308; 0.918) | 0.904 | 0.110 (-0.221; 0.441) | 0.514 |
| Triglycerides | 0.152 (-0.403; 0.708) | 0.591 | 0.042 (-0.377; 0.461) | 0.843 | -0.076 (-0.407; 0.255) | 0.653 |
| Systolic blood pressure | -0.574 (-1.260; 0.112) | 0.101 | 0.364 (-0.020; 0.748) | 0.064 | -0.327 (-0.671; 0.017) | 0.062 |
| Diastolic blood pressure | -0.439 (-1.114; 0.236) | 0.203 | 0.213 (-0.751; 1.177) | 0.665 | -0.083 (-0.415; 0.248) | 0.622 |

**Note:**  All the values are reported estimate (95% confidence interval). HOMA - IR, homeostatic model assessment of insulin assessment; LDL - C, low-density lipoprotein; HDL-C, high-density lipoprotein.
